# Supplementary material for: Efficacy of combined administration of Baekhogainsam-Tang and low-dose pilocarpine on frequent intractable xerostomia: Study protocol for a randomized controlled trial
Source: PLoS One. 2024 Nov 11;19(11):e0307557. doi: 10.1371/journal.pone.0307557 (PMC11554038; doi:10.1371/journal.pone.0307557)
Supplement: S2 Appendix — (PDF) [file pone.0307557.s002.pdf]

임상시험 계획서

Clinical Study Protocol

다빈도 난치성 구강건조증에 대한 필로카르핀-한약제제(백호가인삼탕) 병용 투여 효과를 검증하기 위한 무작위배정, 공개, 평행군, 다기관 임상시험

Effect of Combined Administration of Pilocarpine and Herbal medicine (Baekhogainsam-Tang Ext. Granule) on frequent intractable dry mouth: A randomized, open-label, parallel, multi-center trial

Protocol No: KHMC-Xerostomia-01

임상시험단계	연구자임상
임상시험책임자	은영규
임상시험실시기관	경희대학교병원
최종 버전/발행일	Version 2.6 / 2023.10.19

Version 2.6

계획서 Version History

No	Version No.	Version Date	변경내용
1	1.0	2020.10.07	해당없음
2	2.0	2021.01.21	선정기준 변경 (노인성 구강건조증 제외)<br>제외기준 추가 (병용금지 약물 사용자, 부작용관련 질환, 남성 피임 관련자)<br>병용금지 약물 추가<br>스크리닝 검사 임상실험실 검사의 항목 추가<br>이상반응 평가에 생화학검사의 검사항목 추가
3	2.1	2021.07.01	Protocol No. 오기 수정<br>OHIP-14 설문지 오기 수정
4	2.2	2021.07.27	임상시험의약품 처방 일수 오기 수정
5	2.3	2021.08.15	임상시험의약품 제형 오기 수정
6	2.5	2022.02.16	임상시험대상자 제외기준 수정<br>강동경희대병원 추가로 대상자 수 조정 및 시험대상자번호 부여 방법 추가
7	2.6	2023.11.06	예상 탈락율 변경<br>이에 따른 시험대상자수 변경<br>환자 모집기간을 1년 연장함

임상시험계획서 개요

제 목	다빈도 난치성 구강건조증에 대한 필로카르핀-한약제제(백호가인삼탕) 병용 투여 효과를 검증하기 위한 무작위배정, 공개, 평행군, 다기관 임상시험<br>Effect of Effect of Combined Administration of Pilocarpine and Herbal medicine (Baekhogainsam-Tang Ext. Granule) on frequent intractable dry mouth: A randomized, open-label, parallel, multi-center trial
임상단계	연구자임상
목 적	한약과 저용량 Pilocarpine의 병용 투여가 상용용량 Pilocarpine 단독 투여와 비교하여 방사선구강건조증, 쇼그렌증후군 환자들의 구강건조 치료에 비열등한 효과를 가지며, Pilocarpine의 부작용을 완화시킴을 검증하고자 함.
임상시험 책임자	은영규 / 경희대병원 이비인후과
공동연구자	이준희 / 경희대한방병원 사상체질과
임상약사	윤경원 / 경희의료원 약제본부, 약사<br>홍혜정 / 경희의료원 약제본부, 약사
공동 임상시험 실시기관	경희대학교병원 / 서울시 동대문구 경희대로 23 (02447)<br>인하대병원 / 인천시 중구 인항로 27 / 김지원<br>명지병원 / 경기도 고양시 덕양구 화수로 14번길 55 / 정수영<br>강동경희대학교병원 / 서울특별시 강동구 동남로 892 / 이영찬
시험대상자	방사선구강건조증, 쇼그렌증후군 등 구강건조를 호소하는 환자
선정/제외 기준	● **선정 기준**<br>1. 다음 중 한 개 이상 해당<br>• 두경부영역에 방사선치료를 받은 병력<br>• 쇼그렌증후군으로 진단 (다음의 두 가지 조건을 충족)<br>- Anti-Ro/SSA 검사 양성<br>- Saliva flow rate (Unstimulated) ≤ 0.1mL/min<br>2. Salivary flow test (SFR) 중 unstimulated test 에서 0.25 ml / min 미만<br>3. 최근 1 개월 구강건조에 대한 Visual Analog Scale 상 4 점 이상<br>4. 만 19 세 이상<br>5. 임상시험 참여에 서면으로 동의한 자<br><br>● **제외 기준**

	1. 아래의 질환이 있거나 과거력이 있는 자<br>• 심부전<br>• 고혈압 약물 치료에도 불구하고 수축기혈압 160 mmHg 이상 또는 이완기혈압 100 mmHg 이상인 환자<br>• 기관지천식 또는 그 병력이 있는 환자<br>• 임상증상(실신, 가슴 두근거림, 호흡곤란, 무력감)을 동반하는 부정맥, 치료가 필요하거나 임상적 문제를 일으킬 수 있는 주요 부정맥 (동기능부전, 방실전도차단, 심방세동, 심방조동, 방실접합부 의존성 빈맥, 심실성 빈맥)<br>• 관상동맥(심장동맥)질환<br>• 천식, 만성기관지염 또는 만성 폐쇄성 폐질환<br>• 선천성 아미노산대사이상<br>• 간성혼수 또는 간성혼수 우려가 있음<br>• 협우각녹내장, 말초성 망막증, 홍채염 및 기타 필로카르핀 사용이 금기인 안과 질환을 가진 자.<br>2. 쿠마딘(Coumadin), 헤파린(Heparin), 와파린(Warfarin), 아스피린(단, 혈전 예방 목적으로 사용되는 저용량 아스피린은 예외로 함), 항콜린제, 항콜린효과가 있는 1 세대 항히스타민제, 항콜린효과가 있는 항우울제, 모노아민산화효소억제제(MAO inhibitor), 이뇨제, 미네랄로코르티코이드제, β-아드레날린 길항제나 디지탈리스제(강심제)를 복용 중인 자(단, β-아드레날린 길항제를 복용중인 자는 심장전문의의 판단에 따라 위험성이 크지 않을 경우 임상시험에 참여할 수 있다.)<br>3. 임상시험약 투여 시작일 전 7 일 이내에 실시한 혈액 검사결과가 다음 기준에 해당하는 자<br>• 헤모글로빈 8.0g/dL 미만<br>• AST (GOT) 및 ALT (GPT)가 시험기관 기준치 상한의 3.0 배 초과<br>• 총빌리루빈이 시험기관 기준치 상한의 2.0 배 초과<br>• 크레아티닌이 시험기관 기준치 상한 1.5 배 초과 또는 크레아티닌 클리어런스 (실측치 또는 Cockcroft/Gault 공식에 의한 추정치)가 60mL/min 이하인 경우<br>4. 임신 또는 모유수유를 하는 자, 적절한 피임법을 사용하지 않은 자(경구, 주사법, 주입 또는 호르몬 피임방법, 자궁 내 장치 및 차단 방법)<br>5. 남성 시험자의 적절한 피임법을 사용하지 않은 경우, 시험약 투여종료 후 4 주간의 피임에 동의하지 않은 자(남성과 여성 모두)

	6. 갈락토오스 불내성(galactose intolerance), Lapp 유당분해효소 결핍증(Lapp lactase deficiency) 또는 포도당-갈락토오스 흡수 장애(glucose-galactose malabsorption)<br>7. 임상시험약의 경구 투여가 어려운 자<br>8. 백호가인삼탕 및 이 약물의 주요 구성성분에 임상적으로 유의한 과민방을 나타낸 자<br>9. 필로카르핀에 임상적으로 유의한 과민반응을 나타낸 자<br>10. 스크리닝 검사결과를 비롯한 기타 사유로 연구자의 판단에 따라 임상시험 참여에 부적합하다고 판단한 자<br>11. 인지기능 저하가 있거나, 읽기 및 쓰기가 불가능한 자.
시험군<br>대조군	1. 시험군(Combined Administration of Pilocarpine and Herbal medicine)<br>: 다음 두 가지 병용<br>• 필로겐정(pilocarpine hydrochloride) 1/2 용량<br>• 백호가인삼탕<br>2. 대조군(Conventional Care, CPC)<br>: 필로카르핀 단독 투여<br>• 필로겐정(pilocarpine hydrochloride) 상용 용량

임상시험약

- **임상시험약 1**

제품명	필로겐정 Pilogen Tab.
제조사	씨티씨바이오
품목허가일	2008년 6월 27일
성상 및 제형	흰색의 원형 필름코팅정
성분/함량	Pilocarpine Hydrochloride 염산필로카르핀 5mg
허가된 효능효과	두부암 또는 경부암에 대한 방사선요법에 의해 일어나는 타액선의 기능저하로 인한 구강건조증의 치료<br>쇼그렌증후군 환자의 구강건조증 또는 안구건조증 치료
복용방법	시험군: 1회 0.5정(2.5mg), 1일 4회, 12주간 경구 투여<br>대조군: 1회 1정(5mg), 1일 4회, 12주간 경구 투여<br>(4회: 아침, 점심, 저녁 식사 후, 취침 전)
저장방법	기밀용기, 실온보관(1-30℃)

- **임상시험약 2**

제품명	한풍백호가인삼탕엑스과립 (일반의약품)<br>Baekhogainsamtang Ext. Granule Hanpoong
제조사	한풍제약
품목허가일	1979년 9월 18일
성상 및 제형	회갈색의 과립제
성분/함량	1회 용량 4g 중

배합목적	성분명	규격	분량 (mg)	활성물질용량
주성분	지모 Anemarrhena Rhizome	KP	2,000	
주성분	석고 Gypsum	KHP	5,330	
주성분	감초 Licorice	KP	660	글리시리진산으로서 13.2mg
주성분	갱미 Oryzae Semen	KHP	3,300	
주성분	인삼 Ginseng	KP	1,000	진세노사이드 Rb1 으로서 2.0mg

	수침연조엑스		830	
부형제	유당수화물	KP	2,260	
첨가제	옥수수전분	KP	적량	
첨가제	히드록시프로필 셀룰로우스	KP	적량	
첨가제	백당지방산 에스테르	KP	적량	
첨가제	스테아르산 마그네슘	KP	적량	
첨가제	경질무수규산	KP	적량	
	합계		4,000	100%

허가된 효능효과	목이 마르고, 화끈거림이 있는 증상
복용방법	시험군 : 1회 1포(4g), 1일 3회, 12주간 경구 투여<br>(3회: 아침, 점심, 저녁 식사 전)<br>대조군 : 미투여
저장방법	기밀용기, 실온보관(1-30℃)

시험대상자 수 및 산정근거

- 시험대상자 수
- 총 120 명

	시험군	대조군
	필로카르핀(1/2용량)+ 백호가인삼탕	필로카르핀 (상용용량)
시험대상자수	60	60

- 임상시험실시기관 별 시험대상자 수

	경희대학교병원	인하대병원	명지병원	강동경희대병원	합계
시험군	32	16	2	10	60
대조군	32	16	2	10	60
합계	64	32	4	20	120

- 기관 별 경쟁적 모집
- 경희대학교병원 시험대상자 수: 총 64명 (시험군 32명, 대조군 32명)
- 산정근거
- 본 임상시험은 방사선구강건조증, 쇼그렌증후군 환자 중 구강건조를 일정 정도 이상 호소하는 환자들을 대상으로 무작위로 시험군 및 대조군에 배정하여 시험군에는 '필로카르핀(1/2 용량)+백호가인삼탕 병용투여', 대조군에는 '필로카르핀(상용용량) 단독투여'를 제공하여 그 유효성(시험군이 대조군 대비 비열등함) 및 안전성(대조군에 비해 이상반응이 적음)을 비교평가하는 목적으로 무작위배정, 공개, 평행, 다기관 임상시험의 형태로 설계함.
- 유효한 시험대상자수를 산정하기 위한 가정은 다음과 같음
- 단측 Z검정 (비열등성 검정)
- 유의수준(Level of significance, α) = 0.05 (연구자임상시험임을 고려하여 0.05로 설정함)
- 제2종 오류(β) = 0.1 (검정력(Power of the test) = 90%)
- 시험군 : 대조군 = 1 : 1 (λ=1)
- 제1차 유효성 평가변수 = 12주 후 Salivary flow test (SFR)
- σ = 0.35 / d(비열등성한계) = 0.21

	- 참고문헌으로부터 필로카르핀 5mg 투여 12주 후 SFR은 0.17±0.13이었고, 위약 투여 12주 후 SFR은 0.38±0.48이었음. 이를 기반으로 산출한 표준편차(within)은 0.35로 도출되었음.<br>- 연구자임상이며 임상시험의 탐색적 측면을 고려하여 참고문헌을 참고하여 필로카르핀 5mg 및 위약 투여 12주 후 μclH - μplH의 점추정치 (0.38-0.17=0.21)를 비열등성 한계로 설정하였음<br>- .참고문헌: Vivino FB, et al. Pilocarpine tablets for the treatment of dry mouth and dry eye symptoms in patients with Sjögren syndrome: a randomized, placebo-controlled, fixed-dose, multicenter trial. P92-01 Study Group. Arch Intern Med. 1999. PMID: 9927101 Clinical Trial.<br>- H01 : μc - μt > 0.21<br>H11 : μc - μt ≤ 0.21<br>μc = 12주간 필로카르핀(상용용량) 단독 투여 후 SFR<br>μt = 12주간 필리카르핀(1/2용량), 백호가인삼탕 병용 투여 후 SFR<br>- Nc=Nt= $\frac{(1+\lambda)\sigma^2(Z_\alpha+Z_\beta)}{\lambda d^2}$ ≒48<br>- 탈락율 20% 고려하면 군 당 60명, 총 120명의 시험대상자가 필요
시험방법	진단 기준에 따라 방사선구강건조증, 쇼그렌증후군을 가진 환자 중 VAS 4 이상의 구강건조를 호소하는 환자를 대상으로 무작위로 시험군(한약+저용량 필로카르핀 병용 투여) 및 대조군(상용용량 필로카르핀 단독투여)에 배정하여 12주간 투약 후, 유효성 및 안전성을 비교 평가하는 무작위 배정(randomization), 공개(open-label), 평행군(parallel), 다기관 연구자 임상시험의 형태로 진행한다.
병용요법	없음
병용가능 약물	'병용금지약물'을 제외한 약물은 허용하되, 스크리닝 검사 시 및 임상시험 진행 중에 임상시험담당자의 검토를 받는다.
병용금지 약물	쿠마딘(Coumadin), 헤파린(Heparin), 와파린(Warfarin), 아스피린(단, 혈전 예방 목적으로 사용되는 저용량 아스피린은 예외로 함), 항콜린제, 항콜린효과가 있는 1세대 항히스타민제, 항콜린효과가 있는 항우울제, 모노아민산화효소억제제(MAO inhibitor), 이뇨제, 미네랄로코르티코이드제, β-아드레날린 길항제나 디지탈리스제(강심제)를 복용 중인 자(단, β-아드레날린 길항제를 복용중인 자는 심장전문의의 판단에 따라 위험성이 크지 않을 경우 임상시험에 참여할 수 있다.)

유효성 평가변수	1. 일차평가변수<br>- 12주 후 Unstimulated Salivary Flow Rate (SFR) test 측정치<br>(비자극, 자극 각각의 상태에서 침을 모으는 방식으로 측정됨.<br>최소 1시간 이상 금식 후 5분 동안 시험관에 자연스럽게 생성된 침을 뱉어서 모인 양을 측정하여 비자극 타액분비량을 계산함.)<br>2. 이차평가변수<br>- 12주 후 Unstimulated SFR의 기저치 대비 변화량 및 변화율<br>- Stimulated Salivary Flow Rate (SFR) test (무설탕 레몬향 캔디를 입에 물고 있는 동안 같은 방식으로 5분간 생성된 침을 시험관에 뱉어서 모인 침의 양을 측정하여 자극 타액분비량을 계산함)<br>- 구강 건강 관련 삶의 질 (OHIP-14: The 14-item Oral Health Impact Profile) 설문지 점수의 기저치 대비 변화량 및 변화율<br>- Visual analogue scale (구강 건조)의 기저치 대비 변화량 및 변화율<br>- Salivary scintigraphy에 의한 양측 이하선과 악하선의 섭취율 (UR: uptake ratio), 최대 축적 (MA: maximum accumulation), 최대 분비 (MS: maximum secretion) 등의 기저치 대비 변화량 및 변화율
안전성 평가항목	이상반응, 신체검사, 임상실험실 검사, 활력징후
자료분석	**1. 일반적 원칙**<br>- 인구학적 정보 평가는 무작위 배정된 모든 시험대상자를 대상으로 한다.<br>- 안전성 평가는 안전성 분석군을 대상으로 하며, 이는 임상시험에 참여하여 최소한 1회 이상 임상시험용의약품을 투여 받은 시험대상자를 대상으로 한다.<br>- 유효성 평가는 원칙적으로 ITT 분석군과 PP 분석군을 모두 실시하며, 분석결과가 다른 경우에는 보수적인 PP 분석군을 주 분석법으로 하고,

ITT 분석군은 보조 분석법으로 분석하여 그 결과를 ITT 분석결과와 비교한다. ITT 분석군 : 임상시험에 참여하여 임상시험용의약품을 최소한 1회 이상 투여 받고 유효성 평가변수 측정이 1회 이상 이루어진 시험대상자를 대상으로 한다. 이때 어떤 시점에서 결측치가 발생되거나 임상시험이 종료되기 전에 시험대상자가 탈락하면 가장 최근에 얻은 자료를 마치 해당시점에서 얻어진 것처럼 자료 분석을 실시한다(Last observation Carried Forward Analysis).

- PP 분석군 : ITT 분석군에 포함되는 시험대상자 중 임상시험 계획서대로 완료한 시험대상자로부터 얻어진 자료를 분석에 포함시킨다. '13.3. 임상시험계획서 위반에 대한 처리' 내용 중 복약순응도 70% 미만을 포함하여 '중대한 임상시험계획서 위반'에 해당하는 경우 PP분석군에서 제외시킨다.

2. 통계분석

- 인구학적 기본자료 분석은 본 임상시험에 포함된 모든 시험대상자의 자료를 각 군 별로 평가하며 연속형 자료는 평균, 표준편차, 최소 & 최대치 등을 구하고 범주형 자료의 경우는 절대 및 상대빈도를 구한다. 무작위배정의 타당성을 확인하기 위해 시험군과 대조군 간의 인구 통계학적 자료와 기저치 자료를 비교 평가한다. 분포검정을 수행한 후에 이에 따라 연속형 변수는 t-test(또는 Mann-Whitney U test)로, 범주형 변수는 Chi-square test(또는 Fisher's exact test) 나 필요시 stratification factor를 고려한 Cochran-Mantel-Haenszel Method를 이용하여 비교한다.
- 1차 유효성 평가변수에 대한 분석으로 신뢰구간을 사용한다. 12주 후 Unstimulated Salivary Flow Rate (SFR) 두 군간 평균차이의 신뢰구간을 구하고, 신뢰구간의 하단이 -비열등성한계 (-0.21)보다 크면 시험군이 대조군에 비해 효과가 열등하지 않다고 결론을 내린다. 추가분석으로 두

	군간 12주 후 Unstimulated Salivary Flow Rate (SFR)에 대해 정규성 검정($p<0.05$)을 수행한 후에 이에 따라 independent t-test 또는 Mann-Whitney U test를 이용하여 비교하며, 기저치에 두 군간에 유의한 차이가 있을 경우에는 기저치를 공변량으로 하는 ANCOVA분석을 실시한다.<br>- 2차 유효성 평가변수에 대한 분석으로 연속형 변수의 경우 자료의 분포에 대한 정규성 검정을 수행하여 비정규 분포를 이루는 자료의 경우 정규분포를 이루도록 log transformation, square root transformation 등의 방법으로 자료를 전환한 후 모수적 방법으로 분석하거나, 또는 비정규 분포 자료를 비모수적 방법으로 분석한다. 군간 비교는 independent t-test(또는 Mann-Whitney U test)를 이용하여 분석한다. 군내 연속형 변수의 비교는 paired t-test(또는 Wilcoxon signed-rank test)를 이용하여 분석한다. 반복측정 자료의 group effect, time effect, group×time effect 등은 repeated-measures ANOVA(또는 repeated-measures ANCOVA)를 통해 분석하고, 자료의 성격에 따라 GEE model test를 통해 분석한다. p-value가 0.05보다 작으면 두 군 간에 유의한 차이가 있다고 판단한다.

❖ 임상시험 일정요약

일정 / 항목	Screening Period	Treatment period											
	-1w – 0w	1w	2w	3w	4w	5w	6w	7w	8w	9w	10w	11w	12w
	V1<br>(-1w~0d)	V2<br>(0w)			V3<br>(4w±2d)				V4<br>(8w±2d)				V5<br>(12w±2d)
서면동의	●												
인구학적 정보	●												
병력조사	●												
신체검사	●	●			●				●				●
활력징후	●	●			●				●				●
임상실험실검사	●				●				●				●
12 lead ECG	●												
무작위배정	●												
필로카르핀 투여[1]		●	●	●	●	●	●	●	●	●	●	●	●
백호가인삼탕 투여[2]		●	●	●	●	●	●	●	●	●	●	●	●
OHIP-14 평가		●			●				●				●
EQ-5D-5L평가		●			●				●				●
VAS 평가	●				●				●				●
Salivary flow test (SFR)	●				●				●				●
Salivary scintigraphy		●											●
이상반응 모니터링		●			●				●				●
병용약물 확인		●			●				●				●

1) 필로카르핀 : 시험군 = 1회 0.5정(필로겐정 2.5mg), 1일 4회 / 대조군 = 1회 1정(필로겐정 5.0mg), 1일 4회

2) 백호가인삼탕 : 시험군 = 1회 1포(백호가인삼탕 4g), 1일 3회 / 대조군 = 미투여

※ 1주에 진행하는 기저치(Baseline) 평가는 대상자의 일정이 가능하다면 Screening일(0주)에 서면동의를 받은 후 진행이 가능함.

※ Screening 검사에 포함된 동일한 검사항목를 동일 병원에서 이미 시행한 경우 해당 검사의 결과를 screening 검사로 인정하고 그 유효기간은 검사 후 30일까지이다.

목차

❖ 임상시험 일정요약 13
1. 임상시험 제목 및 단계 19
1.1. 임상시험 제목 19
1.2. 임상시험 단계 19
2. 임상시험실시기관의 명칭 및 주소 19
2.1. 임상시험실시기관의 명칭 및 주소 19
2.2. 공동연구기관 및 연구책임자 19
3. 임상시험 책임자, 공동연구자 및 담당자, 임상약사 19
3.1. 임상시험책임자 19
3.2. 공동연구자 19
3.3. 임상시험담당자 20
3.4. 임상시험용 의약품 등을 관리하는 약사의 성명 및 직명 20
4. 임상시험의 목적 및 배경 20
4.1. 임상시험의 목적 20
4.2. 임상시험의 배경 20
4.2.1. 질환에 대한 배경 20
4.2.2. 기존 치료법에 대한 배경 21
4.3. 임상시험용의약품에 대한 배경 22
4.4. 임상시험의 필요성 25
5. 임상시험용 의약품 정보 26
5.1. 임상시험용 의약품의 개요 26
5.1.1. 필로카르핀 27
5.1.2. 백호가인삼탕 27
5.2. 임상시험용 의약품의 포장 및 라벨링 28
5.3. 임상시험용 의약품의 관리 및 보관방법 29
6. 임상시험의 기간 29
7. 대상질환 29
8. 시험대상자의 선정기준, 제외기준, 목표한 시험대상자의 수 및 산정근거 29
8.1. 선정기준 29

8.2. 제외기준 **30**
8.3. 목표한 시험대상자의 수 **31**
8.4. 시험대상자 산정 근거 **32**
9. 임상시험의 방법 **33**
9.1. 임상시험의 설계 및 계획 **33**
9.2. 연구일정표 **34**
9.3. 임상시험용 의약품의 투여량, 투여방법, 투여기간 **35**
9.4. 병용요법 **35**
9.5. 시험대상자 무작위배정 **35**
9.6. 시험대상자 등록 및 무작위배정 방법 **35**
9.7. 무작위배정의 운영 및 보관 **35**
9.8. 시험대상자 번호 **36**
9.9. 임상시험일정 **36**
9.9.1. 스크리닝 방문 36
9.9.2. 방문 2~5 (Visit 2~5, Week 0~12) 37
9.10. 구급약 **38**
9.11. 병용금지약물 **38**
9.12. 병용허용약물 **38**
10. 관찰항목, 임상검사항목 및 관찰검사항목 **38**
10.1. 스크리닝 평가항목 **38**
10.1.1. 일반적 사항 평가 38
10.1.2. 활력징후 및 신체검진 39
10.1.3. 12-lead ECG (심전도) 39
10.1.4. 임상실험실검사 39
10.1.5. Salivary flow rate (SFR) test 40
10.1.6. 구강건조증에 대한 Visual Analog Scale 평가 40
10.2. 유효성 평가항목 **40**
10.2.1. Salivary flow rate (SFR) test 40
10.2.2. 구강건조증에 대한 Visual Analog Scale 평가 40
10.2.3. Salivary scintigraphy 검사 41
10.2.4. 구강 건강 관련 삶의 질 (OHIP-14: The 14-item Oral Health Impact Profile) 설문지 41

10.3. 안전성 평가항목 ... 42
10.3.1. 이상반응 평가 ... 42
10.3.2. 활력징후 ... 43
10.3.3. 혈액검사 및 소변검사 ... 43
10.4. 경제성 평가 항목 ... 43
11. 예측 부작용 및 사용상의 주의사항 ... 44
11.1. 필로겐정 ... 44
11.1.1. 예측부작용 ... 44
11.1.2. 사용상의 주의사항 ... 46
11.1.3. 과량투여시의 처치 ... 49
11.1.4. 기타 ... 50
11.1.5. 보관 및 취급상의 주의사항 ... 51
11.2. 한풍백호가인삼탕 ... 51
11.2.1. 예측부작용 ... 51
11.2.2. 사용상의 주의사항 ... 51
12. 시험대상자의 임상시험 참여 중지 및 탈락 기준 ... 52
12.1. 참여 중지 및 탈락 기준 ... 53
12.2. 중지 및 탈락 시의 처치 ... 53
12.3. 임상시험계획서 위반에 대한 처리 ... 53
13. 효과 평가기준, 평가방법 및 해석방법(통계분석방법 등) ... 54
13.1. 통계분석 집단 및 일반적인 원칙 ... 54
13.1.1. 인구학적 정보 평가 집단 ... 54
13.1.2. 안전성 평가 집단 ... 54
13.1.3. 유효성 평가 집단 ... 54
13.2. 인구학적 기본자료 ... 55
13.3. 유효성 평가 변수에 대한 분석 ... 55
13.3.1. 1차 유효성 평가 ... 55
13.3.2. 2차 유효성 평가 ... 56
13.4. 안전성 평가 변수에 대한 분석 ... 57
14. 부작용을 포함한 안전성의 평가기준, 평가방법 및 보고방법 ... 58
14.1. 안전성 관련 용어의 정의 ... 58
14.1.1. 이상반응(adverse events) ... 58
14.1.2. 이상약물반응(Adverse Drug Reaction, ADR) ... 59

14.1.3. 중대한 이상반응(Serious adverse events) 59
14.2. 이상반응의 평가 59
14.2.1. 이상반응의 중증도 평가기준 59
14.2.2. 이상반응의 인과관계 평가 60
14.3. 이상반응 및 중대한 이상반응에 대한 기록 61
14.4. 이상반응의 보고 61
14.4.1. 이상반응의 보고 61
14.4.2. 중대한 이상약물반응의 보고 62
14.5. 이상반응/이상약물반응의 추적관찰 62
14.6. 임상시험 후 시험대상자의 진료 및 치료기준 63
15. 연구윤리 63
15.1. 임상시험심사위원회(IRB) 63
15.2. 시험대상자 동의 63
15.3. 비밀보장 64
15.4. 시험대상자 안전보호에 관한 대책 64
16. 기타 임상시험을 안전하고 과학적으로 실시하기 위하여 필요한 사항 65
16.1. 증례기록서 (CRFs) 65
16.2. 전산시스템의 사용 66
16.3. 자료의 질 보장 및 자료보안 66
16.4. 기록의 관리 및 보관 67
16.5. 임상시험 실시기관의 모니터링 67
17. 참고문헌 68

1. 임상시험 제목 및 단계

1.1. 임상시험 제목

- 다빈도 난치성 구강건조증에 대한 필로카르핀-한약제제(백호가인삼탕) 병용 투여 효과를 검증하기 위한 무작위배정, 공개, 평행군, 다기관 임상시험

1.2. 임상시험 단계

- 연구자임상

2. 임상시험실시기관의 명칭 및 주소

2.1. 임상시험실시기관의 명칭 및 주소

- 경희대학교병원 / 서울시 동대문구 경희대로 23

2.2. 공동연구 임상시험실시기관 및 책임자

- 명지병원 / 경기도 고양시 덕양구 화수로 14번길 55 / 정수영
- 인하대학교부속병원 / 인천시 중구 인항로 27 / 김지원
- 강동경희대병원 / 서울특별시 강동구 동남로 892 / 이영찬

3. 임상시험 책임자, 공동연구자 및 담당자, 임상약사

3.1. 임상시험책임자

- 경희대학교병원 이비인후과 은영규

3.2. 공동연구자

- 경희대학교한방병원 사상체질과 이준희

3.3. 임상시험담당자

- 경희대학교병원 이비인후과 박정민
- 경희대학교병원 임상약리학과 이민정

3.4. 임상시험용 의약품 등을 관리하는 약사의 성명 및 직명

윤경원 / 경희의료원 약제본부, 약사
홍혜정 / 경희의료원 약제본부, 약사

4. 임상시험의 목적 및 배경

4.1. 임상시험의 목적

- 한약(백호가인삼탕)과 저용량 Pilocarpine의 병용 투여가 상용용량 Pilocarpine 단독 투여와 비교하여 방사선구강건조증, 쇼그렌증후군 환자들의 구강건조 치료에 비열등한 효과를 가지며, Pilocarpine의 부작용을 완화시킴을 검증하고자 한다.

4.2. 임상시험의 배경

4.2.1. 질환에 대한 배경

- 구강건조증 (xerostomia, dry mouth, oral dryness)은 다양한 원인으로 구강이 마른다고 느끼는 주관적인 증상을 호소하는 질환을 지칭한다. 기전에 따라 침샘 기능은 정상이지만 환자가 주관적으로 구강 건조를 호소하는 가성 구강건조증과 실제로 침샘 기능의 저하로 인하여 발생하는 진성 구강건조증이 있다. 통상적으로 침샘에서 침 분비를 자극 시 분당 0.5~0.7ml 이하, 혹은 비자극 시 분당 0.1ml 이하의 침이 분비되는 경우 타액분비저하증으로 정의하며, 이러한 타액분비저하증의 유무로 가성 및 진성 구강건조증을 구별한다.
- 가성 및 진성 구강건조증을 유발하는 원인에 대한 치료 및 생활환경 개선, 타액선 자극

등을 시도한 후에도 구강건조증이 지속되는 경우를 난치성 구강건조증이라고 한다. 쇼그렌증후군이나 두경부 종양으로 방사선 치료를 받은 환자들의 구강건조증 유병률은 거의 100%에 이른다고 알려져 있으며 (Fox PC. 2007; Shiboski, et al. 2007), 이들은 난치성 구강건조증의 대표적 원인이다.

4.2.2. 기존 치료법에 대한 배경

- 구강건조증의 치료는 근본적으로는 원인이 되는 전신질환에 대한 치료가 필요하며 더불어 복용 약물 및 생활습관과 같은 유발요인이 있다면 이에 대해서 우선적으로 치료가 가능한지를 살펴보아야 한다. 이와 더불어 증상 완화를 위해서 보조적으로 국소적 분무 약제들을 우선적으로 사용해볼 수 있다. 국소적 분무 약제에는 껌 및 사탕을 포함한 타액선 분비자극제, 타액 대체제로 크게 나누어볼 수 있다. 타액선 분비자극제에는 말산을 포함한 자극제, 무설탕 껌, 입안 헹굼제, 젤, 치약 등이 있어서 타액 분비를 자극함. 타액 대체제는 올리브 오일, 베타인, 글리세롤 등을 함유하고 있어 자연 타액과 유사하여 타액 점도를 증가시킴으로써 작용한다.
- 하지만, 난치성 구강건조증을 일으키는 주요 원인인 쇼그렌증후군이나 두경부 종양으로 방사선 치료를 받은 환자들에서는 이러한 치료로는 한계가 있다. 이러한 경우 미국 FDA에서 승인된 전신 타액 분비제인 필로카르핀이나 세비멜린을 복용하여 볼 수 있다. 하지만 쇼그렌증후군에서 이미 자가면역 반응이 많이 진행되었거나 지속적인 방사선 치료 및 노화현상의 결과 타액선 잔여조직이 대부분 소실되어 있는 상태인 경우에는 타액 분비제의 효과에 한계가 있다. 게다가 심한 천식환자나 만성 폐질환, 베타차단제를 복용하고 있는 경우에는 이들 약제의 사용이 금기로 되어있으며 녹내장이나 홍채염이 있는 경우에도 필로카르핀을 사용할 수 없다. 이 외에도 오심, 구토, 설사, 기관지 축소, 빈맥, 저혈압, 시야 이상, 땀 분비 증가 등 많은 부작용이 발생할 수 있다. 즉, 현재 난치성 구강건조증의 치료는 근본적인 치료라기보다는 보조적인 수준에 머물고 있으며, 증상이 진행되어 타액선 잔여조직이 소실될수록 효과는 더 떨어지게 된다. 또한, 전신 타액 분비제는 많은 부작용을 동반하므로 이에 대한 철저한 확인 및 주의가 필요하다.

따라서 보다 근본적인 치료로써 분비 기능이 가능한 침샘의 재생을 돕고 침샘의 염증 반응을 억제하는 융합치료기술의 발견 및 개발, 도입이 요구되는 상황이다.

4.3. 임상시험용의약품에 대한 배경

4.3.1.1. 필로카르핀

- 필로카르핀(Pilocarpine)의 경우 필로겐정 5mg을 사용할 계획이다. 필로카르핀은 남미의 관목인Pilocarpus jaborandi에서 추출한 천연화합물이다. 이 식물성 알칼로이드는 콜린성 부교감 자극제로 muscarinic-M3 수용체에 결합하여 인간의 평활근의 수축을 유발하고 다양한 외분비선 (땀샘, 침샘)을 자극한다. 필로카르핀은 방사선치료 후 발생한 구강건조증을 가진 환자들에서 유효한 효과가 여러 임상연구들에서 입증되었다. 특히, 인공타액, 사탕 그리고 물섭취 등과 비교하였을 때 구강내 건조감과 불편감을 유의하게 호전시켰고, 실제 침의 분비도 증가시켰다. 또한, 쇼그렌증후군을 가진 환자에서도 필로카르핀의 사용은 구강건조와 안구건조에 유의한 증상의 호전이 임상연구에서 입증되었다. 필로카르핀은 국내에서 유일하게 타액분비를 증진시키는 용도로 사용할 수 있는 약제로 허가되었다. 그러나 필로카르핀의 사용은 가슴두근거림, 다한 등의 부작용이 동반되는 경우 있어서 환자들에게 사용에 제한이 있다. 또한 약물의 작용시간이 짧고 누적효과가 적어 자주 그리고 지속적으로 복용하여야 한다는 단점이 있다. 본 연구에서는 대조군은 필로카르핀 상용용량을 시험군은 필로카르핀 상용용량의 1/2을 백호가인삼탕과 함께 투여하는 것으로 설정하였다.

4.3.1.2. 백호가인삼탕

- 백호가인삼탕의 구강건조증에 대한 기존의 대표적인 연구결과는 다음과 같다.
  - 노인 환자의 구강건조 증상에 대하여 백호가인삼탕을 투여한 결과, 30명의 환자 중 60%에서 증상의 개선을 보고되었음. (Masahiro UMINO, et al. Effect of Byakko-ka-Ninjin-to on Xerostomia in elderly patients -Analysis of the relationship between improvement of

subjective symptom and Kampo diagnosis-. Kampo Medicine. 1994;45(1):107-113.)

- Disopyramide phosphate 복용으로 구강건조 및 갈증을 호소하는 환자 11명을 대상으로 12주간 백호가인삼탕을 투여한 결과 백호가인삼탕이 Disopyramide phosphate의 일반적인 부작용인 갈증의 개선에 유효함이 보고되었다. (Shuji YAKUBO, et al. The Effects of Byakko-ka-ninjin-to on Patients in whom Thirst has been induced by Disopyramide Phosphate. Kampo Medicine. 1995;46(3):433-438.)
- 동물실험을 통해 백호가인삼탕이 항콜린제로 유발된 구강건조에 유의한 효과가 있음이 보고되었다.(Masaru Sakaguchi, et al. Effects of Byakko-ka-ninjin-to on salivary secretion and bladder function in rats. J Ethnopharmacol. 2005 Nov 14;102(2):164-9.)

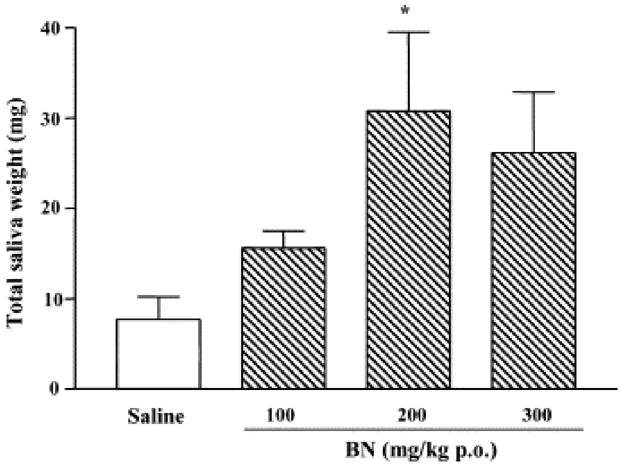

-

Figure 1. Effect of BN on salivary secretion in anesthetized rats. Byakko-ka-ninjin-to was administered p.o. Each column and vertical bar represent the mean ± S.E. of six animals. *P < 0.05 compared to control (saline 500 μL/animal, p.o.) group.

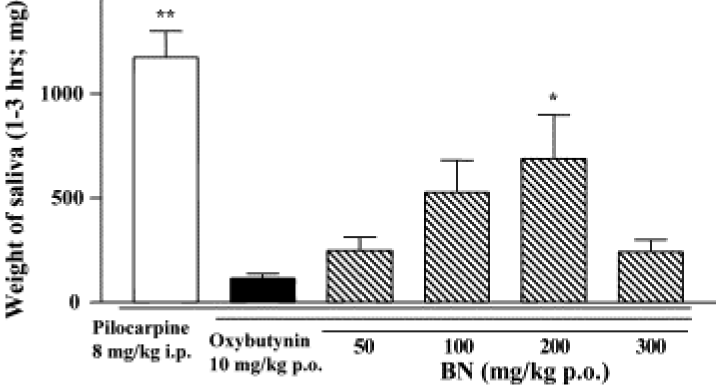

Figure 2. Effect of Byakko-ka-ninjin-to on salivary secretions (1–3 h) in anesthetized rats treated with oxybutynin and pilocarpine. BN was administered p.o. just before administration of oxybutynin. After 10 min, pilocarpine was injected i.p. Each column and vertical bar represent the mean ± S.E. of six animals. **P* < 0.05 and ***P* < 0.01 compared to oxybutynin (10 mg/kg, p.o.) + pilocarpine-treated group (8 mg/kg, i.p.).

- 백호가인삼탕의 투여가 설하침샘으로 작용하는 자율신경에 미치는 영향을 평가하는 동물실험에서 백호가인삼탕이 타액분비를 촉진하는 것으로 보고되었다. (A Niijima, et al. Effect of byakko-ka-ninjin-to on the efferent activity of the autonomic nerve fibers innervating the sublingual gland of the rat. J Auton Nerv Syst. 1997 Mar 19;63(1-2):46-50.)
- 백호가인삼탕은 한풍제약의 한풍백호가인삼탕을 사용할 계획이다. 백호가인삼탕은 일본에서는 Byakko-ka-ninjin-to로 불리는 등 한, 중, 일 한의임상에서 다빈도로 사용되는 처방이다. 한풍백호가인삼탕의 식약처 허가사항에는 '목이 마르고, 화끈거림이 있는 증상'에 효능효과가 있다고 기술되어 있다. 이에 방사선치료로 발생한 구강건조증, 쇼그렌증후군으로 인한 구강건조증에 도움이 되어, 필로카르핀의 투여용량을 줄이면서도 유사한 효과를 보일 것으로 기대된다. 본 임상시험에서는 한풍백호가인삼탕의 허가 용량용법을 참조하여 1일 3회, 1회 4g을 투여하는 것으로 계획하였다.
- 백호가인삼탕의 구강건조증에 대한 장기 치료에 대한 안정성에 대한 기존의 대표적인 연구결과는 다음과 같다.
  - Sprague-Dawley (SD) Rat을 실험동물로 하여 백호가인삼탕(쯔무라제약, TJ-34)에 대해 단회투여독성시험 및 13주 반복투여독성시험을 실시하였다. 단회투여독성시험에서는 SD rat 각 군 10마리(암수 각각 5마리)를 대상으로 2g/kg과 8 g/kg을 투여하였다. 결과로 죽거나 투약 관련한 이상반응은 관찰되지 않았고, 이후 부검에서도 이상소견은 없었다. 치사량은 8g/kg으로 추정된다. 반복투여독성시험에서는 SD rat을 대상으로 13주간 대조군(n=40, 20/sex, 0mg/kg)과 실험군1(n=20, 10/sex, 125mg/kg), 실험군2(n=20, 10/sex, 500mg/kg), 실험군3(n=40, 20/sex, 2000mg/kg)으로 나누어 진행하였으며, 대조군 중 일부(n=20, 10/sex, 0mg/kg)와 실험군3(n=20, 10/sex, 2000mg/kg) 중 일부는 투약 종료

후 4주간 추가적으로 관찰하였다. 결과로 실험 과정 중에 죽거나 투약 관련한 이상반응은 관찰되지 않았고, 체중과 물 및 사료 섭취량은 투약에 영향을 받지 않았다. 투약 종료 후 소변검사 및 안과적 검사에도 투약관련한 변화는 관찰되지 않았고, 혈액학검사와 생화학검사 상에서도 투약관련한 변화는 보이지 않았다. 실험 후 부검에서도 장기무게, 육안적, 조직학적 검사에서도 투약관련한 이상소견은 관찰되지 않았다. 결론적으로 백호가인삼탕의 무독성 용량은 실험실 환경에서 2000mg/kg/day로 추정된다. (Minematsu S., Yoshinaga K., Takei H., et al. A single oral dose toxicity study and a 13-week repeated dose study with a 4-week recovery period of TSUMURA Byakko-ka-ninjin-to (TJ-34) in rats. Japanese Pharmacology and Therapeutics 1995 23:SUPPL. 7 (169-189))

4.4. 임상시험의 필요성

- 필로카르핀과 백호가인삼탕을 병용투여하여 작용시간이 짧고 누적효과가 없는 필로카르핀을 복용 용량을 낮추어 비열등한 효과를 보인다면 필로카르핀의 부작용을 줄일 수 있으면서도 복약순응도를 높이고, 구강건조증의 치료효과 및 환자만족도를 제고시킬 수 있을 것이다. 이에 필로카르핀 1/2용량과 백호가인삼탕을 병용 투여하는 시험군과 필로카르핀 상용용량을 단독 투여하는 대조군을 설정하고, 12주간 투여하여 1차 유효성평가변수인 Unstimulated Salivary Flow Rate를 비교하여 이상을 평가하고자 한다.

4.5. 병용투여 시험설계에 대한 근거

- 필로겐정(필로카르핀)은 muscarinic agonist로 침샘분비를 증가시키는 작용이 우수함.
- 그러나 반감기가 3시간 이내로 짧고 누적효과가 없기 때문에 하루에도 여러 번(2-4회) 복용하여야 한다. 한편 필로카르핀의 부작용은 부교감신경 유사효과의 과다로 특징지어짐. 이에는 두통, 시야 장애, 눈물, 발한, 호흡곤란, 위장관 연축, 구역, 구토, 설사, 방실 차단, 빈맥, 서맥, 저혈압, 고혈압, 쇽, 정신혼동, 부정맥, 진전 등이 있음. 또한 이로 인해 실제 임상에서 구강건조증으로 고통을 받는 환자들이 적지않게 발생하는 약의

부작용으로 사용할 수 있는 약제가 없어 어려움이 많음.

- 이에 침샘분비를 증가시키는 필로카르핀을 상용용량의 1/2로 투여(기존 임상시험에서 필로카르핀 1/2 용량은 상용용량보다 효과가 유의하게 적음이 보고됨)하고, 침샘분비를 증가시키며 필로카르핀의 침샘분비 증가 작용을 향상시키는 백호가인삼탕을 병용 투여하여, 필로카르핀의 투여용량을 줄이면서도 동일한 효과를 보이고 부작용을 감소시키는 것을 검증하고자 하는 것이 본 임상시험의 설계 근거이자 목표가 됨.
- 병용투여의 근거논문

  muscarinic agonist인 필로카르핀은 침샘분비를 유의하게 증가시키나 oxybutynin이나 propiverine같은 항콜린제에 의해 그 작용이 억제된다. 동물실험에서 Oxybutynin에 의해 억제된 필로카르핀의 침샘분비 증가 작용이 백호가인삼탕을 병용투여 했을 때 유의하게 회복되었다.

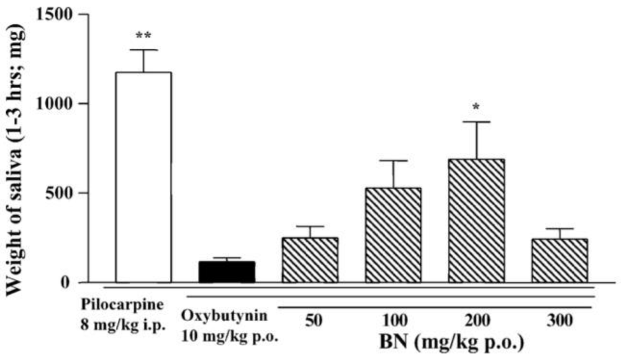

Fig. 4. Effect of BN on salivary secretions (1–3 h) in anesthetized rats treated with oxybutynin and pilocarpine. BN was administered p.o. just before administration of oxybutynin. After 10 min, pilocarpine was injected i.p. Each column and vertical bar represents the mean ± S.E. of six animals. *$P<0.05$ and **$P<0.01$ compared to oxybutynin (10 mg/kg, p.o.) + pilocarpine-treated group (8 mg/kg, i.p.).

(Masaru S., Kazuhiro G., Hiroyuki I., et al. Effects of Byakko-ka-ninjin-to on salivary secretion and bladder function in rats. J Ethnopharmacol. 2005 Nov 14;102(2):164-9.)

5. 임상시험용 의약품 정보

5.1. 임상시험용 의약품의 개요

5.1.1. 필로카르핀

제품명	필로겐정 Pilogen Tab.
제조사	씨티씨바이오
품목허가일	2008년 6월 27일
성상 및 제형	흰색의 원형 필름코팅정
성분/함량	Pilocarpine Hydrochloride 염산필로카르핀 5mg
허가된 효능효과	1. 두부암 또는 경부암에 대한 방사선요법에 의해 일어나는 타액선의 기능 저하로 인한 구강건조증의 치료<br>2. 쇼그렌증후군 환자의 구강건조증 또는 안구건조증 치료.
복용방법	시험군: 1회 0.5정(2.5mg), 1일 4회, 12주간 경구 투여<br>대조군: 1회 1정(5mg), 1일 4회, 12주간 경구 투여<br>(4회: 아침, 점심, 저녁 식사 후, 취침 전)
저장방법	기밀용기, 실온보관(1-30℃)

5.1.2. 백호가인삼탕

<table>
<tr><td>제품명</td><td>한풍백호가인삼탕엑스과립 (일반의약품)
Baekhogainsamtang Ext. Granule Hanpoong</td></tr>
<tr><td>제조사</td><td>한풍제약</td></tr>
<tr><td>품목허가일</td><td>1979년 9월 18일</td></tr>
<tr><td>성상 및 제형</td><td>회갈색의 과립제</td></tr>
<tr><td>성분/함량</td><td>1회 용량 4g 중
<table>
<tr><td>배합목적</td><td>성분명</td><td>규격</td><td>분량(mg)</td><td>활성물질용량</td></tr>
<tr><td>주성분</td><td>지모
Anemarrhena Rhizome</td><td>KP</td><td>2,000</td><td></td></tr>
<tr><td>주성분</td><td>석고
Gypsum</td><td>KHP</td><td>5,330</td><td></td></tr>
<tr><td>주성분</td><td>감초
Licorice</td><td>KP</td><td>660</td><td>글리시리진산으로서 13.2mg</td></tr>
<tr><td>주성분</td><td>갱미
Oryzae Semen</td><td>KHP</td><td>3,300</td><td></td></tr>
<tr><td>주성분</td><td>인삼
Ginseng</td><td>KP</td><td>1,000</td><td>진세노사이드 Rb1으로서 2.0mg</td></tr>
<tr><td></td><td>수침연조엑스</td><td></td><td>830</td><td></td></tr>
</table></td></tr>
</table>

	부형제	유당수화물	KP	2,260	
	첨가제	옥수수전분	KP	적량	
	첨가제	히드록시프로필 셀룰로우스	KP	적량	
	첨가제	백당지방산에스 테르	KP	적량	
	첨가제	스테아르산마그 네슘	KP	적량	
	첨가제	경질무수규산	KP	적량	
		합계		4,000	100%
허가된 효능효과	목이 마르고, 화끈거림이 있는 증상				
복용방법	시험군 : 1회 1포(4g), 1일 3회, 12주간 경구 투여<br>(3회: 아침, 점심, 저녁 식사 전)<br>대조군 : 미투여				
저장방법	기밀용기, 실온보관(1-30℃)				

5.2. 임상시험용 의약품의 포장 및 라벨링

- 본 연구에 사용되는 임상시험용의약품 중 필로겐정은 씨티씨바이오에서, 한풍백호가인삼탕은 (주) 한풍제약에서 제조되어 경희대학교병원 임상약국으로 전달받아 <의약품 등의 안전에 관한 규칙[총리령 제1022호, 2013.03.23제정]> 제69조 6항에 따라 아래와 같이 라벨링한다. 명지병원과 인하대병원, 강동경희대병원은 경희대학교병원 임상약국에서 라벨링하여 이를 인하대병원과 명지명원, 강동경희대병원 임상약국에 전달한다.
- 임상시험용의약품은 시험대상자 방문일정에 따라 '방문2'부터 '방문4'까지 각 30일분을 기밀 용기에 포장한다.

1. "임상시험용"이라는 표시
2. 제품의 코드명 또는 주성분의 일반명
3. 제조번호 및 사용(유효)기한 또는 재검사일자
4. 저장방법
5. 임상시험계획 승인을 받은 자의 상호와 주소
6. "임상시험 외의 목적으로 사용할 수 없음"이라는 표시

5.3. 임상시험용 의약품의 관리 및 보관방법

- 임상시험용의약품의 관리약사는 임상시험에 사용되는 의약품의 인수, 보관, 조제, 관리 및 반납에 대한 책임을 가진다. 관리약사는 임상시험용의약품의 수령사실 및 수량을 서면으로 확인하고 서명해야 하며 적절히 관리하여야 한다.
- 임상시험용의약품은 연구자의 처방에 따라 관리약사에 의해 투약되며, 관리약사는 각 시험대상자에게 지급된 임상시험용의약품 및 관리에 대한 기록을 정확히 하여야 한다.
- 본 임상시험은 공개 임상시험이며 시험대상자 식별번호로 식별하며 각 시험대상자에게 처방된 임상시험용의약품과 회수된 임상시험용의약품에 대해서는 적절히 기록되어야 한다.
- 임상시험의 중지 및 종료 시 관리약사는 미사용된 임상시험용의약품을 경희대학교병원 임상약국에 반납하고 반납증을 보관여야 하며 경희대학교병원 임상약국은 반납된 임상시험용의약품을 폐기하도록 한다.

6. 임상시험의 기간

- 임상시험 계획서 IRB 승인일 ~ 4년

7. 대상질환

- 방사선구강건조증, 쇼그렌증후군 등 구강건조를 호소하는 환자

8. 시험대상자의 선정기준, 제외기준, 목표한 시험대상자의 수 및 산정근거

8.1. 선정기준

- 다음 중 한 개 이상 해당

- 두경부영역에 방사선치료를 받은 병력
- 쇼그렌증후군으로 진단 (다음의 두 가지 조건을 충족)

  (1) Anti-Ro/SSA 검사 양성

  (2) Saliva flow rate (Unstimulated) ≤ 0.1mL/min

- Salivary flow test (SFR) 중 unstimulated test에서 0.25 mL/min 미만
- 최근 1개월 구강건조에 대한 Visual Analog Scale 상 4점 이상
- 만 19세 이상
- 임상시험 참여에 서면으로 동의한 자

8.2. 제외기준

- 아래의 질환이 있거나 과거력이 있는 자
  - 심부전
  - 고혈압 약물 치료에도 불구하고 수축기혈압 160 mmHg 이상 또는 이완기혈압 100 mmHg 이상인 환자
  - 기관지천식 또는 그 병력이 있는 환자
  - 임상증상(실신, 가슴 두근거림, 호흡곤란, 무력감)을 동반하는 부정맥, 치료가 필요하거나 임상적 문제를 일으킬 수 있는 주요 부정맥 (동기능부전, 방실전도차단, 심방세동, 심방조동, 방실접합부 의존성 빈맥, 심실성 빈맥)
  - 관상동맥(심장동맥)질환
  - 천식, 만성기관지염 또는 만성 폐쇄성 폐질환
  - 선천성 아미노산대사이상
  - 간성혼수 또는 간성혼수 우려가 있음
  - 협우각녹내장, 말초성 망막증, 홍채염 및 기타 필로카르핀 사용이 금기인 안과 질환을 가진 자.
- 임상시험약 투여 시작일 전 7일 이내에 실시한 혈액 검사결과가 다음 기준에 해당하는 자

- 헤모글로빈 8.0g/dL 미만
- AST (GOT) 및 ALT (GPT)가 시험기관 기준치 상한의 3.0배 초과
- 총빌리루빈이 시험기관 기준치 상한의 2.0배 초과
- 크레아티닌이 시험기관 기준치 상한 1.5배 초과 또는 크레아티닌 클리어런스 (실측치 또는 Cockcroft/Gault 공식에 의한 추정치)가 60mL/min 이하인 경우

- 쿠마딘(Coumadin), 헤파린(Heparin), 와파린(Warfarin), 아스피린(단, 혈전 예방 목적으로 사용되는 저용량 아스피린은 예외로 함), 항콜린제, 항콜린효과가 있는 1세대 항히스타민제, 항콜린효과가 있는 항우울제, 모노아민산화효소억제제(MAO inhibitor), 이뇨제, 미네랄로코르티코이드제, β-아드레날린 길항제나 디지탈리스제(강심제)를 복용 중인 자(단, β-아드레날린 길항제를 복용중인 자는 심장전문의의 판단에 따라 위험성이 크지 않을 경우 임상시험에 참여할 수 있다.)
- 임신 또는 모유수유를 하는 자, 적절한 피임법을 사용하지 않은 자(경구, 주사법, 주입 또는 호르몬 피임방법, 자궁 내 장치 및 차단 방법)
- 남성 시험자의 적절한 피임법을 사용하지 않은 경우, 시험약 투여종료 후 4주간의 피임에 동의하지 않은 자(남성과 여성 모두)
- 갈락토오스 불내성(galactose intolerance), Lapp 유당분해효소 결핍증(Lapp lactase deficiency) 또는 포도당-갈락토오스 흡수 장애(glucose-galactose malabsorption)
- 임상시험약의 경구 투여가 어려운 자
- 백호가인삼탕 및 이 약물의 주요 구성성분에 임상적으로 유의한 과민반응을 나타낸 자
- 필로카르핀에 임상적으로 유의한 과민반응을 나타낸 자
- 스크리닝 검사결과를 비롯한 기타 사유로 연구자의 판단에 따라 임상시험 참여에 부적합하다고 판단한 자
- 인지기능 저하가 있거나, 읽기 및 쓰기가 불가능한 자.

8.3. 목표한 시험대상자의 수

- 총 120명 (시험군 60명, 대조군 60명)
- 임상시험실시기관 별 시험대상자 수

	경희대학교병원	인하대병원	명지병원	강동경희대병원	합계
시험군	32	16	2	10	60
대조군	32	16	2	10	60
합계	64	32	4	20	120

- 기관 별 경쟁적 모집
- 경희대학교병원 시험대상자 수: 총 64명 (시험군 32명, 대조군 32명)

8.4. 시험대상자 산정 근거

- 본 임상시험은 방사선구강건조증, 쇼그렌증후군 환자 중 구강건조를 일정 정도 이상 호소하는 환자들을 대상으로 무작위로 시험군 및 대조군에 배정하여 시험군에는 '필로카르핀(1/2 용량)+백호가인삼탕 병용투여', 대조군에는 '필로카르핀(상용용량) 단독투여'를 제공하여 그 유효성(시험군이 대조군 대비 비열등함) 및 안전성(대조군에 비해 이상반응이 적음)을 평가하는 목적으로 무작위배정, 공개, 평행, 다기관, 연구자 임상시험의 형태로 설계하였다.
- 유효한 시험대상자 수를 산정하기 위하여 다음을 가정하였다.
  ① 평가변수의 통계적 가설검정은 단측 z-검정(비열등성 검정)으로 한다.
  ② 유의수준(Level of significance, α)은 0.05로 한다. (연구자임상임을 고려하여 0.05로 설정함)
  ③ 제 2종 오류(β)는 0.1로 하여 검정력(Power of the test)은 90%를 유지한다.
  ④ 시험군과 대조군의 대상자수 비율은 1:1로 한다.
  ⑤ 제1차 유효성 평가변수 = 12주 후 Salivary flow test (SFR)
- σ = 0.35 / d(비열등성한계) = 0.21
- 참고문헌으로부터 필로카르핀 5mg 투여 12주 후 SFR은 0.17±0.13이었고, 위약 투여 12주 후 SFR은 0.38±0.48이었음. 이를 기반으로 산출한 표준편차(within)은 0.35로

도출되었음.

연구자임상이며 임상시험의 탐색적 측면을 고려하여 참고문헌을 참고하여 필로카르핀 5mg 및 위약 투여 12주 후 μcIH - μpIH의 점추정치 (0.38-0.17=0.21)를 비열등성 한계로 설정하였음.

- 참고문헌: Vivino FB, et al. Pilocarpine tablets for the treatment of dry mouth and dry eye symptoms in patients with Sjögren syndrome: a randomized, placebo-controlled, fixed-dose, multicenter trial. P92-01 Study Group. Arch Intern Med. 1999. PMID: 9927101 Clinical Trial.
- 본 임상시험의 H01과 H11은 다음과 같다.

H01: μc - μt > 0.21

H11: μc - μt ≤ 0.21

μc = 12주간 필로카르핀(상용용량) 단독 투여 후 SFR

μt = 12주간 필리카르핀(1/2용량), 백호가인삼탕 병용 투여 후 SFR

$$n_c = \frac{(1+\lambda)\sigma^2 (Z_\alpha + Z_\beta)^2}{\lambda(\,|\,\mu_c - \mu_t\,|\, - d)^2}$$

Nc=Nt≒48

- 탈락율 20% 고려하면 군 당 60명, 총 120명의 시험대상자가 필요하다.

	대조군	시험군	합계
최종 평가 연구대상자수	48	48	96
Drop-out(20%) 고려 연구대상자수	60	60	120

9. 임상시험의 방법

9.1. 임상시험의 설계 및 계획

- 본 임상시험은 무작위 배정(randomization), 공개(open-label), 평행(parallel),

다기관(multi-center), 연구자 임상시험의 형태로 진행한다. 방사선구강건조증, 쇼그렌증후군 등 일정 정도 이상의 구강건조를 호소하는 환자를 대상으로 첫 중재일(1주)로부터 7일 이내(-7d)에 문진, 신체검진 및 임상실험실검사 등 스크리닝 검사를 시행하여, 본 임상시험에 적합하다고 판단되는 시험대상자를 선정한다. 선정된 시험대상자는 필로카르핀 1/2용량과 백호가인삼탕을 병행 투여하는 시험군과 필로카르핀 상용용량을 단독으로 투여하는 대조군 중 하나에 무작위로 배정되며, 총 12주간 투여 후 유효성 및 안전성을 비교 평가한다. 시험대상자는 스크리닝을 위한 방문 1회(Visit 1), 등록, 무작위배정 후 임상시험이 종료되는 12주차까지 4회(Visit 2 – Visit 5) 등 총 5회 방문을 하게 된다.

9.2. 연구일정표

일정 / 항목	Screening Period	Treatment period						
	-1w - 0w	1w	2-3w	4w	5-7w	8w	9-11w	12w
	V1 (-1w~0d)	V2 (0w)		V3 (4w±2d)		V4 (8w±2d)		V5 (12w±2d)
서면동의	●							
인구학적 정보	●							
병력조사	●							
신체검사	●	●		●		●		●
활력징후	●	●		●		●		●
임상실험실검사	●			●		●		●
12 lead ECG	●							
무작위배정	●							
필로카르핀 투여1)		●	●	●	●	●	●	●
백호가인삼탕 투여2)		●	●	●	●	●	●	●
OHIP-14 평가		●		●		●		●
EQ-5D-5L평가		●		●		●		●
VAS 평가	●			●		●		●
Salivary flow test (SFR)	●			●		●		●
Salivary scintigraphy		●						●
이상반응 모니터링		●		●		●		●
병용약물 확인		●		●		●		●

9.3. 임상시험용 의약품의 투여량, 투여방법, 투여기간

		투여량	투여방법	투여기간
시험군	필로겐정	1회 2.5mg, 총 4회	1일 4회 (아침, 점심, 저녁 식후 30분, 취침 전)	12주
	한풍백호가인삼탕	1회 4g, 총 3회	1일 3회 (아침, 점심, 저녁 식전 30분)	12주
대조군	필로겐정	1회 5mg, 총 4회	1일 4회 (아침, 점심, 저녁 식후 30분, 취침 전)	12주

9.4. 병용요법

- 없음

9.5. 시험대상자 무작위배정

9.6. 시험대상자 등록 및 무작위배정 방법

- 기관별로 층화하여 확률론적 이론에 따른 층화무작위배정법으로 시험군과 대조군에 1:1로 배정한다.
- 무작위배정방법은 기관별로 층화하여 각 층화별로 무작위배정코드가 시험군과 대조군으로 주어지는 층화블록(4블록) 무작위배정법을 실시한다.
- 무작위배정은 본 임상시험과 독립된 통계전문가가 Microsoft Windows용 SAS ver.9.1.3(SAS Institute Inc., NC, Cary, USA)을 이용하여 무작위 배정번호를 부여하여 무작위배정표를 작성한다.

9.7. 무작위배정의 운영 및 보관

- 무작위배정표가 작성되면 통계전문가가 관리한다.
- 시험대상자는 연구에 대한 설명을 듣고 임상시험 참여에 대한 동의서를 작성하게 되면, 스크리닝번호를 순서대로 부여받게 된다.

- 스크리닝검사를 통해 최종적으로 임상시험 참여에 선정된 시험대상자는 등록번호를 부여받게 되고, 통계전문가가 보관 중인 무작위배정표에 근거하여 시험대상자등록번호에 따른 군을 할당받게 된다.

9.8. 시험대상자 번호

- 임상시험에 참여하고자 서면 동의한 자원자에게 서면 동의 시간 순서대로 스크리닝 번호(screening number)를 부여한다. 스크리닝 번호는 KH-S001 로 시작하고, 'KH-'를 뺀 나머지는 전체 네 자리, 숫자 세 자리로 구성된다(경희대병원 'KH-', 인하대부속병원 'IH-', 명지병원 'MJ-', 강동경희대병원 'KD-'). 최종적으로 스크리닝 통과가 결정된 날짜 순서대로 (동일한 날짜인 경우 동의 시간 순서대로) 시험대상자 식별번호 (subject number)를 부여하며, 미리 작성된 무작위배정표에 따라 해당 군 에 배정된다. 시험대상자 식별번호는 각 KH-R1001로 시작하고, 'KH-'를 뺀 나머지는 전체 다섯 자리, 숫자 네 자리로 구성된다(경희대병원 'KH-', 인하대부속병원 'IH-', 명지병원 'MJ-', 강동경희대병원 'KD-'), 식별번호는 다음과 같이 부여한다. 첫번째 자리의 1(혹은 2)은 해당 군을, 끝에 세자리는 무작위배정 순서대로 부여된다. 각 시험대상자에게 부여된 스크리닝 번호, 시험대상자 식별번호는 임상시험이 끝날 때까지 시험대상자를 인식하는 코드로 사용된다.

9.9. 임상시험일정

9.9.1. 스크리닝 방문

- 임상시험에 참여하고자 서면 동의한 환자를 대상으로 시험대상자의 적합성 여부를 검토하기 위한 스크리닝 검사를 첫 중재일(1주)로부터 7일 이내(-7d)에 최소 8시간 이상 공복상태에서 시행하며 다음 검사에 의해 임상적으로 유의한 이상이 있는 대상자는 제외한다. 참고로, 스크리닝 결과 해당 검사항목의 변화가 일시적 변화소견으로 판단될 경우, 첫 번째 중재일 이전 재검사를 통해 적합성 여부를 재검토할 수 있다.

- 인구학적 정보 및 병력조사.
- 활력징후 및 신체검진
- 임상실험실검사 (혈액학 검사 / 생화학 검사 / 뇨검사 / 면역학 검사 / 혈액응고검사 / 혈청학적 검사)
- 12-lead ECG (심전도)
- Salivary flow rate (SFR) test
- 구강건조증에 대한 Visual Analog Scale 평가

9.9.2. 방문 2~5 (Visit 2~5, Week 0~12)

- 스크리닝 검사를 통해 선정 및 제외기준에 의거하여 임상시험 참여에 적합하고, 자발적으로 서면 동의한 시험대상자는 두 가지 군 중 하나에 무작위로 배정된다.
- 시험군 및 대조군 각각의 정해진 임상시험약 4주 분량을 4주 간격으로 교부 받는다 (투여 전 (0주, 방문2)투여 4주 후(4주±2일, 방문3), 8주 후(8주±2일, 방문4))
- 임상시험 투여 전(0주, 방문2), 투여 4주 후(4주±2일, 방문3), 8주 후(8주±2일, 방문4), 12주 후(12주±2일, 방문5) 등 매 방문 시에 다음의 검사가 진행될 예정이다.
  - 복약순응도 평가 및 미투여 임상시험약 반납
  - 이상반응 및 병용 투여 약물 확인
  - 활력징후 및 신체검진
  - 임상실험실검사 (혈액학 검사, 생화학 검사, 뇨검사)
  - Salivary flow rate (SFR) test
  - 구강건조증에 대한 Visual Analog Scale 평가
  - Salivary scintigraphy 검사
  - 구강 건강 관련 삶의 질 (OHIP-14: The 14-item Oral Health Impact Profile) 설문지
  - 삶의질 (EQ-5D-5L) 설문지

9.10. 구급약

- 별도의 구급약 없음.

9.11. 병용금지약물

쿠마딘(Coumadin), 헤파린(Heparin), 와파린(Warfarin), 아스피린(단, 혈전 예방 목적으로 사용되는 저용량 아스피린은 예외로 함), 항콜린제, 항콜린효과가 있는 1세대 항히스타민제, 항콜린효과가 있는 항우울제, 모노아민산화효소억제제(MAO inhibitor), 이뇨제, 미네랄로코르티코이드제, β-아드레날린 길항제나 디지탈리스제(강심제)를 복용 중인 자(단, β-아드레날린 길항제를 복용중인 자는 심장전문의의 판단에 따라 위험성이 크지 않을 경우 임상시험에 참여할 수 있다.)

9.12. 병용허용약물

- 상기 '9-11. 병용금지약물'을 제외한 약물은 허용하되, 스크리닝 검사 시 임상시험담당자의 검토를 받는다.

10. 관찰항목, 임상검사항목 및 관찰검사항목

10.1. 스크리닝 평가항목

- 다음의 항목에 대한 평가 및 검사를 실시하여 선정 및 제외기준에 의거 임상시험 참여에 적합한 시험대상자를 선정한다.

10.1.1. 일반적 사항 평가

- 성별, 연령
- 과거 병력, 최근 병력, 약물복용력
- 흡연유무, 흡연량, 알코올섭취유무, 알코올섭취량
- 두경부 수술 및 방사선치료 병력

10.1.2. 활력징후 및 신체검진

- 혈압 및 맥박수 (급격한 체위 변동 없이 3분 이상 좌위를 유지한 상태에서 측정)
- 체온 (고막체온).
- 신장(cm), 체중(kg)

10.1.3. 12-lead ECG (심전도)

- automatic analysis & recording이 출력되는 ventricular rate (beats/min), PR interval (msec), QRS (msec), QT/QTc (msec)을 기록한다.

10.1.4. 임상실험실검사

10.1.4.1. 혈액학 검사

- WBC with differential count (segmented neutrophil, lymphocyte, monocyte, eosinophil, basophil), RBC, hemoglobin, hematocrit, platelet

10.1.4.2. 생화학 검사

- glucose, BUN, uric acid, cholesterol, total protein, albumin, total bilirubin, alkaline phosphatase, AST, ALT, γ-GTP, CPK, LDH, creatinine, sodium, potassium, chloride, triglyceride, HDL, LDL, C-reactive protein, phosphorus, calcium

10.1.4.3. 뇨검사

- pH, specific gravity, protein, bilirubin, glucose, urobilinogen, ketone, nitrite, blood, leukocyte, microscopy (Urine RBC, Urine WBC, Urine Epithelial Cell, others)

10.1.4.4. 면역학 검사

- 항핵항체 (ANA), 항쇼그렌증후군 A항체 (Anti-Ro/SSA), 항쇼그렌증후군 B항체 (Anti-La/SSB),

10.1.4.5. 혈액응고검사

- PT, aPTT

10.1.4.6. 혈청학적검사

- HBsAg, anti-HBs Ab, anti-HCV Ab, IgM anti-HAV Ab

10.1.5. Salivary flow rate (SFR) test

- 비자극, 자극 각각의 상태에서 침을 모으는 방식으로 측정한다.
- (비자극) 최소 1시간 이상 금식 후 5분 동안 시험관에 자연스럽게 생성된 침을 뱉어서 모인 침의 양을 측정하여 비자극 타액분비량을 계산한다.
- (자극) 무설탕 레몬향 캔디를 입에 물고 있는 동안 같은 방식으로 5분간 생성된 침을 시험관에 뱉어서 모인 침의 양을 측정하여 자극 타액분비량을 계산한다.

10.1.6. 구강건조증에 대한 Visual Analog Scale 평가

- 구강건조 항목을 평가한다.
- 증상들의 정도는 시각 아날로그 평가척도 (VAS: visual analog scale; 불편감 없음을 0, 참을 수 없는 매우 심한 불편감을 10으로 하여 현재 통증의 척도를 수치화한 것)를 이용하여 평가한다.

10.2. 유효성 평가항목

10.2.1. Salivary flow rate (SFR) test

- 방문2 (0주)는 스크리닝검사 수치로 대체한다.
- 방문3 (4주), 방문4 (8주), 방문5 (12주)에 각각 검사한다.

10.2.2. 구강건조증에 대한 Visual Analog Scale 평가

- 방문2 (0주)는 스크리닝검사 수치로 대체한다.
- 방문3 (4주), 방문4 (8주), 방문5 (12주)에 각각 검사한다.

10.2.3. Salivary scintigraphy 검사

- 방문2 (0주), 방문5 (12주)에 각각 검사한다.
- 침샘 기능의 객관적인 평가를 위해서 방사선물질 (Technetium-99m pertechnetate: Tc-99m pertechnetate) 투여 후 Salivary scintigraphy를 시행한다.
- Tc 99m sodium pertechnetate 370 MBq를 우선 환자에게 정맥 투여 후 저에너지-고민감도 콜리메이터 방식의 듀얼헤드 감마 카메라 (Philips Forte, Philips Medical Systems)를 이용해 영상을 획득한다.
- 영상은 방사성의약품 정맥 투여 후 5분, 15분, 30분 후 획득하며, 또한 타액선 분비자극제 투여 후 같은 방식으로 전면 영상을 획득한다.
- 검사하는 동안 환자의 머리는 고정하며 침을 삼키지 않도록 지시한다.
- 영상을 분석하기 위해서 4개의 주타액선인 양측 이하선과 악하선, 배경방사능을 측정하기 위한 측두골에 각각 관심영역 (ROI: regions of interest)을 설정하여 섭취율 (UR: uptake ratio), 최대 축적 (MA: maximum accumulation), 최대 분비 (MS: maximum secretion) 세 가지 척도를 계산함으로써 침샘 기능을 평가한다.
- 섭취율은 각 침샘의 '최고방사능 – 배경방사능 (방사선 물질을 투여하지 않아도 기본적으로 측정되는 방사량)'으로 계산한다.
- 최대 축적(%)은 '(자극 전 최고 방사능 – 방사성의약품 투여 5분 후 방사능) / (자극 전 최고 방사능) *100'으로 계산한다.
- 최대 분비 (%)는 '(자극 전 최고 방사능 – 자극 후 방사능) / (자극 전 최고 방사능) *100'으로 계산한다.

10.2.4. 구강 건강 관련 삶의 질 (OHIP-14: The 14-item Oral Health Impact Profile) 설문지

- 방문2 (0주), 방문3 (4주), 방문4 (8주), 방문5 (12주)에 각각 검사한다.

- 구강 건강 관련 삶의 질을 설문지를 이용하여 주관적 삶의 질을 평가한다.

입안의 병 때문에 다음과 같은 상황이 얼마나 자주 발생하나요?				
	4점 전혀 안 그렇다	3점 가끔 그렇다	2점 종종 그렇다	1점 자주 그렇다
발음이 어렵다				
맛을 느끼기 힘들다				
통증이 있다				
음식을 먹을 때 불편하다				
자신감이 떨어진다				
긴장되어 부자연스럽다				
식사하는 것이 만족스럽지 못하다				
식사에 방해가 된다				
긴장을 풀기가 어렵다				
조금 당혹스러울 때가 있다				
다른 사람들을 대하는 것이 짜증스럽다				
일방적인 일을 하는데 방해가 된다				
일상적인 생활이 만족스럽지 못하다				
전반적인 활동이 지장이 있다				

10.3. 안전성 평가항목

10.3.1. 이상반응 평가

- 치료 부작용의 정도를 평가하는 grading system으로 가장 널리 쓰이는 MedDRA ver. 23.1 (Korean)를 바탕으로 분류하고 평가한다.
- 임상시험용의약품 투여 시작 시점부터 투여 종료 시 까지 매 방문 시에 이상반응 발생여부 및 등급을 평가한다.
- 기본적인 grading의 원칙은 아래와 같다.

Grade 1: 증상은 있으나 치료가 필요하지 않은 부작용<br>Grade 2: 증상을 경감시킬 치료가 필요하나 기본적인 일상생활 (ADL)을 방해하지 않고

생명의 위협을 주지 않는 부작용<br>Grade 3: 치료하지 않고 그대로 두었을 때 생명에 위협을 가할 수 있는 부작용<br>Grade 4: 생명에 위협을 가하는 부작용

10.3.2. 활력징후

- 매 방문 시마다 측정한다.
- 혈압, 맥박수, 호흡수, 체온

10.3.3. 혈액검사 및 소변

- 스크리닝 방문(V1), 임상시험용의약품 의약품 투여 4주 후(V3), 투여 8주 후(V4), 투여 12주 후(V5)에 실시하며, 그 항목은 다음과 같다.

10.3.3.1. 혈액학 검사

- Hemoglobin, Hematocrit, RBC, WBC, WBC differential cell count, Platelet

10.3.3.2. 생화학검사

- Alkaline phosphatase, BUN, Creatinine, γ-GTP, SGPT(ALT), SGOT(AST), Albumin, Protein, Total bilirubin, CRP, LDH, sodium, potassium, chloride, phosphorus, calcium

10.3.3.3. 뇨검사

- pH, specific gravity, Protein, Glucose, Ketones, Bilirubin, Blood, Urobilinogen, Nitrite, WBC

10.3.3.4. 혈액응고검사

- PT, aPTT

10.4. 경제성평가항목

- 방문2 (0주), 방문3 (4주), 방문4 (8주), 방문5 (12주)에 각각 검사한다.
- 건강관련 삶의 질을 설문지를 이용하여 주관적 삶의 질을 평가한다.
- 향후 경제성평가를 위한 삶의 질 척도로 이용한다

건강관련 삶의 질(EQ-5D-5L)

각 항목에 대하여 당신의 현재 건강상태를 가장 잘 표현하는 문장을 하나만 골라주세요.

1. 운동 능력

☐ 나는 걷는데 전혀 문제가 없다.
☐ 나는 걷는데 약간의 문제가 있다.
☐ 나는 걷는데 중간 정도의 문제가 있다.
☐ 나는 걷는데 심한 문제가 있다.
☐ 나는 걸을 수 없다.

2. 자기 관리

☐ 나는 스스로 씻거나 옷 갈아 입는데 전혀 문제가 없다.
☐ 나는 스스로 씻거나 옷 갈아 입는데 약간의 문제가 있다.
☐ 나는 스스로 씻거나 옷 갈아 입는데 중간 정도의 문제가 있다.
☐ 나는 스스로 씻거나 옷 갈아 입는데 심한 문제가 있다.
☐ 나는 스스로 씻거나 옷을 갈아 입을 수 없다.

3. 일상 생활(일, 공부, 가정 생활, 오락 활동 등)

☐ 나는 일상 생활을 하는 데 전혀 문제가 없다.
☐ 나는 일상 생활을 하는 데 약간의 문제가 있다.
☐ 나는 일상 생활을 하는 데 중간 정도의 문제가 있다.
☐ 나는 일상 생활을 하는데 심한 문제가 있다.
☐ 나는 일상 생활을 할 수가 없다.

4.통증 / 불편함

☐ 나는 전혀 통증이나 불편함이 없다.
☐ 나는 약간의 통증이나 불편함이 있다.
☐ 나는 중간 정도의 통증이나 불편함이 있다.
☐ 나는 심한 정도의 통증이나 불편함이 있다.
☐ 나는 극심한 정도의 통증이나 불편함이 있다.

5. 불안감 / 우울감

☐ 나는 전혀 불안하거나 우울하지 않다.
☐ 나는 약간의 불안감이나 우울감이 있다.
☐ 나는 중간 정도의 불안감이나 우울감이 있다.
☐ 나는 심한 정도의 불안감이나 우울감이 있다.
☐ 나는 극심한 정도의 불안감이나 우울감이 있다.

10.5. 복약순응도 평가

- 임상시험용의약품의 투여량에 대하여 방문 시 시험대상자가 지참하고 온 임상시험용의약품의 잔량을 확인하여 평가하다.
- 복약순응도 (%) = [(처방량 – 반납량) / 계획된 복용량] * 100

11. 예측 부작용 및 사용상의 주의사항

11.1. 필로겐정

11.1.1. 예측부작용

(1) 두부암 또는 경부암 환자

- ① 때때로 다음의 이상반응이 나타날 수 있다.
  - 발한, 오심, 비염, 설사, 한기, 홍조, 빈뇨, 현기증, 무력증, 두통, 소화불량, 유루, 부종, 복통, 약시, 구토, 인두염, 고혈압, 시각이상, 결막염, 연하곤란, 울체, 근육통, 소양감, 발진, 부비강염, 빈맥, 미각도착증, 진전, 음성변화
- ② 드물게 다음의 이상반응이 나타날 수 있다.
  - 전신 : 체취, 저체온, 점막 이상
  - 심혈관계 : 서맥, ECG 이상, 심계항진, 실신
  - 소화기계 : 식욕부진, 식욕증가, 식도염, 위장관 장애, 혀의 질환
  - 조혈계 : 백혈구 감소증, 임파절 질환
  - 신경계 : 불안, 착란, 우울, 과운동증, 지각감퇴, 신경과민, 감각이상, 언어장애, 연축
  - 호흡기계 : 객담의 증가, 천명, 하품
  - 피부 : 지루
  - 특수감각 : 난청, 눈의 통증, 녹내장
  - 비뇨생식기 : 배뇨장애, 자궁출혈, 요로장애
- ③ 장기간의 치료에서 심혈관계 기저질환을 가진 두명의 환자가 각각 심근 경색과 실신을 경험하였다. 약물과의 인과관계는 불명확하다.

(2) 쇼그렌증후군 환자

- ① 2개의 위약대조군 시험에서 이 약과 관련된 이상반응 중 가장 빈번한 것은 발한, 빈뇨, 한기, 혈관확장(홍조)이며,환자가 치료를 중단한 가장 빈번한 이유는 발한이었다.
- 때때로 다음의 이상반응이 나타날 수 있다.
  - 발한, 빈뇨, 오심, 홍조, 비염, 설사, 한기, 침분비 증가, 무력증, 두통, 유행성 감기, 소화불량, 현기증, 통증, 부비강염, 복통, 인두염, 발진, 감염, 우발적 외상, 알러지반응, 배통, 시야의 흔들림, 변비, 기침증가, 부종, 오한, 안면부종, 발열, 방귀, 설염, 화학적・혈액학적 뇨분석을 포

함한 실험실적 검사치의 이상, 근육통, 심계항진, 소양감, 졸음, 위염, 빈맥, 이명, 뇨실금, 요로감염, 질염, 구토

- ② 드물게 다음의 이상반응이 나타날 수 있다.
  - 전신 : 흉통, 낭포, 사망, 모닐리아(칸디다)증, 경부통증, 경부경직, 광과민반응
  - 심혈관계 : 협심증, 부정맥, ECG 이상, 저혈압, 고혈압, 두개내 출혈, 편두통, 심근경색
  - 소화기계 : 식욕 감퇴, 빌리루빈혈증, 담석증, 결장염, 구강 건조, 트림, 위염, 위장염, 위장관 이상, 치은염, 간염, 간기능 검사 비정상, 하혈, 구역 및 구토, 췌장염, 이하선 비대, 타액선 비대, 객담 증가, 미각 상실, 혀 이상, 치아 이상
  - 조혈계 : 혈뇨, 림프절 장애, 혈소판이상, 혈소판혈증, 혈소판감소증, 혈전증, 백혈구 이상
  - 대사와 영양 : 말초 부종, 저혈당증
  - 골격근육계 : 관절통, 관절염, 골 이상, 자연적 골절, 병리학적 골절, 근무력증, 건질환, 치아 질환
  - 신경계 : 실어증, 착란, 우울증, 비정상적 꿈, 정서 불안, 과운동증, 지각 감퇴증, 불면증, 다리 경련, 신경과민증, 이상 감각증, 비정상적 사고, 진전
  - 호흡기계 : 기관지염, 호흡곤란, 딸꾹질, 후두 경련, 후두염, 폐렴, 바이러스 감염, 음성 변화
  - 피부 : 탈모증, 접촉성 피부염, 피부 건조, 습진, 결절성 홍반, 박탈성 피부염, 단순포진, 피부 궤양, 대소수성 발진
  - 특수감각 : 백내장, 결막염, 안구 건조, 이(귀)질환, 이통, 안질환, 안출혈, 녹내장, 유루 질환, 망막 질환, 미각 도착, 시각 이상
  - 비뇨생식기 : 유방 통증, 배뇨 곤란, 유방염, 월경과다, 자궁 출혈, 난소 질환, 농뇨, 난관염, 요도 통증, 빈뇨, 질출혈, 질 모닐리아(칸디다)증

11.1.2. 사용상의 주의사항

11.1.2.1. 경고사항

- 1) 심한 심혈관계 질환 환자 : 이 약에 의해 유발된 혈류동태 또는 박동의 이상을 보정할 수

없었다. 심혈관계 질환 환자에게 이 약을 투여할 경우, 의사의 철저한 감독하에서 신중히 투여하여야 한다.

- 2) 눈 : 눈에 이 약을 적용한 환자에서, 특히 야간에 또한 시력교정을 받은 환자에게 시각이 저하되는 시야의 혼탁이 일어났으며, 심각한 지각장애의 원인이 되는 것으로 보고되었다. 밤에 운전을 하거나 어두운 곳에서 위험한 일을 할 때 주의가 요망된다.
- 3) 이 약은 기도의 저항력, 기관지 평활근의 긴장, 기관지 분비를 증가시킨다는 보고가 있다. 천식, 만성기관지염 또는 만성 폐색성질환 환자에게 이 약을 투여할 경우, 의사의 철저한 감독하에서 신중히 투여하여야 한다.

11.1.2.2. 투여 금기 환자

- 1) 천식환자
- 2) 필로카르핀 과민증 환자
- 3) 축동이 유익하지 않은 경우 : 급성 홍채염이나 협우각 녹내장(폐쇄우각 녹내장)환자

11.1.2.3. 일반적 주의

- ① 이 약을 담석증 환자나 담석증이 의심되는 환자 또는 담도질환 환자에게 투여할 때에는 주의하여야 한다. 담낭 또는 담관 평활근의 위축은 담낭염, 담관염, 담도폐색을 포함한 합병증을 촉진시킬 수 있다.
- ② 이 약은 요관 평활근의 긴장을 증가시킬 수 있으며, 이론적으로 특히 신결석증 환자에 있어서 신산통(또는 요관 역류)을 촉진시킬 수 있다.
- ③ 콜린 효능약은 용량과 관련하여 중추신경계 효과를 지닌다. 이것은 기초적인 인식을 지닌 환자 또는 정신의학적인 장애환자 치료시에 고려되어야 한다.
- ④ 이 약을 투여하면 특히 밤에 안전하게 운전할 수 없을 정도의 시각장애를 일으킬 수 있다는 것을 환자에게 알려야 한다.
- ⑤ 이 약을 복용하는 중 충분한 수분을 섭취할 수 없는 상황일 때 환자에게 발한이 과다하게

나타난다면 탈수증이 일어날 수 있다.

- ⑥ 이 약의 독성은 부교감신경 유사 효과의 과다로 특징지어진다. 이는 두통, 시야 장애, 눈물, 발한, 호흡곤란, 위장관 연축, 구역, 구토, 설사, 방실 차단, 빈맥, 서맥, 저혈압, 고혈압, 쇽, 정신혼동, 부정맥, 진전을 포함한다.
- 이 약의 용량과 관련된 심혈관계 약리작용은 저혈압, 고혈압, 서맥, 빈맥을 포함한다.
- ⑦ 중등도 간장애 환자에서 혈중 청소율이 감소한다는 것에 근거하여 이 환자들에서의 초기 용량은 1회 5mg을 1일 2회로 하고, 치료반응과 내약성에 따라 조절한다.
- 경증 간장애 환자(Child-Pugh score 5~6)는 용량감소가 필요하지 않다. 중증 간 장애(Child-Pugh score 10~15)를 가진 피험자에서의 약동력학적 연구는 수행되지 않았다. 이런 환자들에게는 이 약물의 사용은 권장되지 않는다

11.1.2.4. 상호작용

- 이 약은 심장 전도장애의 가능성 때문에 β-아드레날린 길항제를 투여받고 있는 환자에게는 주의하여 투여한다.
- 부교감신경 흥분작용을 지닌 약물을 이 약과 동시에 투여하면, 상가적인 약리효과가 예상된다. 이 약과 항콜린 효능약(아트로핀, 흡입용 이프라트로피늄)을 동시에 사용하면 그 효과를 길항한다.
- 전형적인 약물상호작용 실험은 수행되지 않았지만, 다음의 약물들이 쇼그렌환자의 효능실험에 최소 10%의 환자에게 병용투여되었다. 아세틸살리실산, 인공누액, 칼슘, 결합형 에스트로겐, 황산 히드록시클로로퀸, 이부프로펜, 레보타이록신 나트륨염, 초산 레보타이록신, 초산 메드록시프로게스테론, 메토트렉세이트, 나프록센, 오메프라졸, 파라아세타몰, 프레드니손

11.1.2.5. 임부에 대한 투여

- 이 약을 임신한 암컷 랫트에서 1일 90㎎/㎏(성인 1일 최대량의 26배)을 경구투여하였을 때, 평균 태자체중의 감소와 골격변화의 증가와 관련이 있었다. 임신기간 및 수유기간동안 1일

36㎎/㎏(성인 1일최대량의 10배)을 경구투여하였을 때, 사망률이 증가되었고, 18㎎/㎏(성인 1일 최대량의 5배)와 이를 초과한 용량의 투여로 갓난 새끼의 생존 및 새끼의 평균 체중의 감소가 관찰되었다. 임신한 여성에 대한 적절하고 잘 계획된 임상시험은 없으므로 임신기간에 이 약을 투여할 경우, 유익성이 태아에 대한 위험성을 상회할 때에만 사용하여야 한다.

11.1.2.6. 수유부에 대한 투여

- 이 약이 모유에서 분비되는지 여부는 알려지지 않았다. 그러나 많은 약이 모유로 이행, 분비되고 또한 수유중 유아에게 일련의 이상반응을 줄 가능성이 있기 때문에 모체에 대한 이 약의 유익성을 감안하여 수유를 중지하거나 또는 투약중단을 고려하여야 한다.

11.1.2.7. 소아에 대한 투여

- 소아에서의 안전성과 유효성은 입증되지 않았다.

11.1.2.8. 고령자에 대한 투여

- 1) 두부암 또는 경부암 환자 : 15명의 고령 자원자 시험에서 5명의 여성이 10명의 남성에 비해 높은 최대혈중농도와 곡선하면적(AUC)을 나타냈다
- 2) 쇼그렌증후군의 고령환자에게 투여시 빈뇨, 설사, 현기증의 증가경향이 보고된 바 있다.

11.1.3. 과량투여시의 처치

- 이 약은 100㎎ 이상 투여시 치명적일 가능성이 있다고 생각된다. 과용량에 대한 치료는 아트로핀(0.5㎎~1.0㎎)을 피하 또는 정맥투여하고, 환자의 체력유지에 유효한 호흡과 순환의 유지를 조정하여야 한다. 또한 심한 심혈관기능 저하나 기관지 수축이 있을 때에는 에피네프린(0.3㎎~1.0㎎)을 피하 또는 정맥투여하는 것이 바람직하다. 이 약이 투석 가능한 지는 알려져 있지 않다.

11.1.4. 기타

- 1) 신질환 및 간질환 환자에게 이 약을 경구투여할 경우, 이에 대한 약력학은 알려지지 않았다.
- 2) 발암성: 생존시 경구 발암성 연구가 CD-1 마우스와 SD 랫트에서 실시되었다. 이 약은 어떤 용량의 시험(최대 30mg/kg/day까지로 임상적으로 관찰되는 최대 전신투여보다 약 50배 더 투여한 용량)에서도 마우스에서 암을 유발하지 않았다. 랫트에서는 임상적으로 관찰되는 최대 전신투여보다 약 100배 더 투여한 용량인 18mg/kg/day에서 수컷과 암컷에서 양성 갈색세포종의 발현 및 암컷 랫트에서 간세포선종 발현이 통계적으로 유의한 증가를 보였다. 랫트에서 관찰된 발암성은 최대 임상 허용량의 수배에서만 나타났으며 임상사용과는 관련이 없는 것으로 보인다.
- 3) 변이원성: 다음 연구들을 통해 이 약이 유전독성을 야기할 수 있다는 증거가 얻어지지 않았다.
- ① 역유전자 변이를 위한 세균평가(살모넬라와 대장균)
- ② 중국 햄스터 난소세포주에서의 in vitro 염색체 변이 평가
- ③ 마우스에서의 in vivo염색체 변이 평가(micronucleus)
- ④ 랫트 간세포 배양에서 일차 DNA손상 평가(비예정된 DNA 합성)
- 4) 수정능력 손상: 이 약을 수컷 및 암컷 랫트에 18mg/kg/day용량(임상적으로 관찰되는 최대 전신투여보다 약 100배 더 투여한 용량)으로 경구투여시 생식능 감소, 정자운동성 감소, 비정상 정자의 형태적 증거 등을 포함하는 손상된 생식능을 나타냈다. 생식능의 감소가 수컷 혹은 암컷 동물에 대한 영향인지, 양쪽 모두에 대한 영향인지는 불명확하다. 개에게 본제를 3mg/kg/day용량으로 6개월간 투여(성인 최대량의 약 3배)했을 때, 정자 생성능의 손상 결과를 보였다. 이 결과는 이 약이 남성과 여성의 생식능에 손상을 줄 수도 있음을 나타낸다.
- 임신을 계획중인 사람에게는 이 약의 유익성이 생식능 손상을 상회할 때에만 투여해야 한다

11.1.5. 보관 및 취급상의 주의사항

- 1) 어린이의 손이 닿지 않는 곳에 보관하여야 한다.
- 2) 다른 용기에 바꾸어 넣는 것은 사고원인이 되거나 품질유지면에서 바람직하지 않으므로 이를 주의하여야 한다.

11.2. 한풍백호가인삼탕

11.2.1. 예측부작용

- (1) 피부 : 발진, 두드러기 등
- (2) 소화기계: 식욕부진, 위부불쾌감, 묽은 변, 설사 등
- (3) 위알도스테론증 : 요량이 감소하거나 얼굴과 손발이 붓고, 눈꺼풀이 무거워지고, 손이 굳어지고, 혈압이 높아지거나 두통 등(1일 최대 복용량이 감초로서 1g 이상인 제제는 장기간 계속하여 복용할 경우 저칼륨혈증, 혈압상승, 나트륨 체액의 저류, 부종, 체중증가 등의 위알도스테론증이 나타날 수 있으므로, 관찰(혈청칼륨치의 측정)을 충분히 하고 이상이 확인되는 경우 복용을 중지할 것.)
- (4) 근병증 : 저칼륨혈증의 결과로서 근병증이 나타날 수 있으므로, 관찰을 충분히 하고 무력감, 사지경련, 마비 등의 이상이 확인되는 경우 복용을 중지할 것.

11.2.2. 사용상의 주의사항

- (1) 다음과 같은 사람은 이 약을 복용하기 전에 의사, 한의사, 치과의사, 약사, 한약사와 상의할 것.
- 1) 고혈압 환자
- 2) 심장애 또는 신장애 환자
- 3) 부종 환자
- 4) 위장이 허약하고 냉증이 있는 환자(식욕부진, 위부불쾌감, 묽은 변, 설사 등이 나타날 수 있다.)

- 5) 몸이 허약한 환자(체력이 쇠약해진 환자, 몸이 약한 환자)(이상반응이 쉽게 나타나고 그 증상이 악화될 수 있다.)
- 6) 임부 또는 임신하고 있을 가능성이 있는 여성
- 7) 의사의 치료를 받고 있는 환자(다른 약물을 투여 받고 있는 환자)
- 8) 고령자(일반적으로 고령자는 생리기능이 저하되어 있으므로 감량하는 등 주의할 것.)
- 9) 어린이(어린이에 대한 안전성이 확립되어 있지 않다(사용경험이 적다.).)

- (2) 기타 이 약의 복용시 주의할 사항
- 1) 정해진 용법·용량을 잘 지킬 것.
- 2) 장기간 계속하여 복용하지 않는 것이 원칙이나 부득이 장기간 계속하여 복용할 경우에는 의사, 한의사, 치과의사, 약사, 한약사와 상의할 것.
- 3) 칼륨함유제제, 감초함유제제, 글리시리진산 또는 그 염류 함유제제, 루프계 이뇨제(푸로세미드, 에타크린산) 또는 티아지드계 이뇨제(트리클로르메티아지드)와 병용시 위알도스테론증이나 저칼륨혈증으로 인하여 근병증이 나타나기 쉬우므로 신중히 복용할 것.
- 4) 다른 한약제제 등과 함께 복용할 경우에는 함유 생약의 중복에 주의할 것.

- (3) 저장상의 주의사항
- 1) 직사광선을 피하고 되도록 습기가 적은 서늘한 곳에 보관할 것(사용 후 반드시 밀폐 보관할 것.).
- 2) 어린이의 손이 닿지 않는 곳에 보관할 것.
- 3) 의약품을 원래 용기에서 꺼내어 다른 용기에 보관하는 것은 의약품 오용에 의한 사고 발생이나 의약품 품질 저하의 원인이 될 수 있으므로 원래의 용기에 넣고 꼭 닫아 보관할 것.

12. 시험대상자의 임상시험 참여 중지 및 탈락 기준

12.1. 참여 중지 및 탈락 기준

- 시험대상자 선정에서 제외되어야 할 조건이 스크리닝 이후에 판명된 경우
- 시험대상자 또는 보호자가 임상시험 참가 동의를 철회한 경우
- 임상시험 중 선정 및 제외 기준 등 중대한 임상시험계획서 위반사항이 새롭게 발견되는 경우
- 중대한 이상반응/이상약물반응이 발생한 경우
- 시험대상자가 임상시험용의약품의 안전성 및 유효성을 평가하는데 영향을 줄 것으로 예상되는 약물을 투여한 경우
- 임상시험 중 병용금지약물을 복용한 경우
- 기타 임상시험담당자 및 책임자의 판단에 의해 임상연구의 진행이 적합하지 못하다고 판단되는 경우

12.2. 중지 및 탈락 시의 처치

- 시험대상자가 중지 및 탈락하는 경우에는 임상시험용의약품의 투여를 중지하고 중지 및 탈락 시점까지 획득한 모든 데이터와 함께 중지일(최종 투여일) 및 탈락일, 중지 및 탈락에 대한 사유, 중지 및 탈락시의 처치와 경과를 증례기록서에 기록한다.

12.3. 임상시험계획서 위반에 대한 처리

- 연구자는 임상시험계획서의 위반이 발생하지 않도록 계획서에 대해 충분히 숙지하고 철저히 이행하여야 한다.
- 중대한 임상시험계획서 위반의 경우, 해당 시험대상자를 분석에서 탈락처리(PP분석 제외)함을 원칙으로 하며 해당사항은 다음과 같다.

1) 동의서 미취득
2) 선정 및 제외기준에 위반된 경우
3) 임상시험 기간 동안 병용금지약물을 투약한 경우

4) 임상시험 시작과 종료 시점의 주요검사가 누락된 경우

5) 임상시험약의 복약순응도가 70% 미만인 경우

- 복약순응도를 대상자가 임상시험 기간 동안 복용 완료한 임상시험약의 양을 복용해야할 총량으로 나눈 값으로 정의하고, 이 값이 70% 미만일 경우 탈락시킨다.
- 기타 연구결과 해석에 영향을 미치지 않을 것으로 판단되는 경미한 임상시험계획서의 위반사항은 위반 또는 지연 정도와 사유를 명확히 기재하고, 위반 또는 지연사항이 임상시험에 영향을 주었는지 종합적으로 고찰하여 PP분석에 포함한다.

13. 효과 평가기준, 평가방법 및 해석방법(통계분석방법 등)

13.1. 통계분석 집단 및 일반적인 원칙

13.1.1. 인구학적 정보 평가 집단

- 무작위 배정된 모든 시험대상자를 대상으로 한다.

13.1.2. 안전성 평가 집단

- 안전성(Safety)분석군 : 임상시험에 참여하여 최소한 1회 이상 임상시험용의약품을 투여 받은 시험대상자를 대상으로 한다.
- 안전성 평가의 경우 자료의 보정없이 원자료로 분석한다.

13.1.3. 유효성 평가 집단

- 본 임상시험의 시험대상자로부터 얻어진 자료는 크게 ITT(Intention To Treat) 분석군과 PP(Per Protocol) 분석군의 두 가지 형태로 분석한다.
- ITT 분석군 : 임상시험에 참여하여 임상시험용의약품을 최소한 1회 이상 투여 받고 유효성 평가변수 측정이 1회 이상 이루어진 시험대상자를 대상으로 한다. 이때 어떤 시점에서 결측치가 발생되거나 임상시험이 종료되기 전에 시험대상자가 탈락하면 가장 최근에 얻은 자료를 마치 해당시점에서 얻어진 것처럼 자료 분석을 실시한다(Last observation Carried Forward Analysis).

- PP 분석군 : ITT 분석군에 포함되는 시험대상자 중 임상시험 계획서대로 완료한 시험대상자로부터 얻어진 자료를 분석에 포함시킨다. '13.3. 임상시험계획서 위반에 대한 처리' 내용 중 복약순응도 70% 미만을 포함하여 '중대한 임상시험계획서 위반'에 해당하는 경우 PP분석군에서 제외시킨다.
- 유효성 평가는 원칙적으로 ITT 분석군과 PP 분석군을 모두 실시하며, 분석결과가 다른 경우에는 보수적인 PP 분석군을 주 분석법으로 하고, ITT 분석군은 보조 분석법으로 분석하여 그 결과를 ITT 분석결과와 비교한다.

13.2. 인구학적 기본자료

- 본 임상시험에 포함된 모든 시험대상자의 자료를 각 군 별로 평가하며 연속형 자료는 평균, 표준편차, 최소 & 최대치 등을 구하고 범주형 자료의 경우는 절대 및 상대빈도를 구한다.
- 무작위배정의 타당성을 확인하기 위해 시험군과 대조군 간의 인구 통계학적 자료와 기저치 자료를 비교 평가한다. 분포검정을 수행한 후에 이에 따라 연속형 변수는 t-test(또는 Mann-Whitney U test)로, 범주형 변수는 Chi-square test(또는 Fisher's exact test)나 필요시 stratification factor를 고려한 Cochran-Mantel-Haenszel Method를 이용하여 비교한다.

13.3. 유효성 평가 변수에 대한 분석

13.3.1. 1 차 유효성 평가

13.3.1.1. 평가항목 및 기준

- 12주 후 Unstimulated Salivary Flow Rate (SFR) 측정치

13.3.1.2. 통계분석방법

- 1차 유효성 평가변수에 대한 분석은 신뢰구간을 사용한다. 12주 후 Unstimulated Salivary Flow Rate (SFR) 두 군간 평균차이의 신뢰구간을 구하고, 신뢰구간의 하단이 -

비열등성한계 (-0.21)보다 크면 시험군이 대조군에 비해 효과가 열등하지 않다고 결론을 내린다.

- 추가분석으로 두 군간 12주 후 Unstimulated Salivary Flow Rate (SFR)에 대해 정규성 검정($p<0.05$)을 수행한 후에 이에 따라 independent t-test 또는 Mann-Whitney U test를 이용하여 비교하며, 기저치에 두 군간에 유의한 차이가 있을 경우에는 기저치를 공변량으로 하는 ANCOVA분석을 실시한다.

13.3.2.2 차 유효성 평가

13.3.2.1. 평가항목 및 기준

- 12주 후 Stimulated Salivary Flow Rate (SFR) 측정치
- 12주 후 Unstimulated SFR의 기저치 대비 변화량 및 변화율
- 12주 후 Stimulated SFR의 기저치 대비 변화량 및 변화율
- 구강 건강 관련 삶의 질 (OHIP-14: The 14-item Oral Health Impact Profile) 설문지 점수의 기저치 대비 변화량 및 변화율
- Visual analogue scale (구강 건조) 점수의 기저치 대비 변화량 및 변화율
- Salivary scintigraphy에 의한 양측 이하선과 악하선의 섭취율 (UR: uptake ratio), 최대 축적 (MA: maximum accumulation), 최대 분비 (MS: maximum secretion) 등의 기저치 대비 변화량 및 변화율
- 구강건조증 증상평가 설문지 점수의 기저치 대비 변화량 및 변화율

13.3.2.2. 통계분석방법

- 연속형 변수의 경우 자료의 분포에 대한 정규성 검정을 수행하여 비정규 분포를 이루는 자료의 경우 정규분포를 이루도록 log transformation, square root transformation 등의 방법으로 자료를 전환한 후 모수적 방법으로 분석하거나, 또는 비정규 분포 자료를 비모수적 방법으로 분석한다.

- 군간 비교는 independent t-test(또는 Mann-Whitney U test)를 이용하여 분석한다.
- 군내 연속형 변수의 비교는 paired t-test(또는 Wilcoxon signed-rank test)를 이용하여 분석한다.
- 반복측정 자료의 group effect, time effect, group×time effect 등은 repeated-measures ANOVA(또는 repeated-measures ANCOVA)를 통해 분석하고, 자료의 성격에 따라 GEE model test를 통해 분석한다.
- p-value가 0.05보다 작으면 두 군 간에 유의한 차이가 있다고 판단한다.

13.4. 안전성 평가 변수에 대한 분석

- 이상반응, 임상실험실 검사, 활력징후, 신체검사 등을 분석한다.
- 원칙적으로 안전성(Safety) 평가군을 대상으로 실시하며, 자료의 보정없이 원자료로 분석한다.
- 모든 시험대상자의 활력징후, 신체검진 및 임상실험실검사 결과를 도표화하여 총괄적으로 검토하고 시험대상자 별로 이상여부를 판단하여 이의 임상적 의미를 기재하고 시험약과의 관련성 여부를 검토하며, 임상적으로 유의한 변동을 보인 대상자의 결과는 별도의 표로 제시한다.

13.4.1.1. 이상반응

- 이상반응은 MedDRA ver. 23.1 (Korean)을 기준으로 system organ class (SOC, 기관계대분류)와 Preferred Term (PT, 대표용어) 로 표준화한다.
- 본 임상시험에서 발생한 이상반응 및 약물이상반응은 시험군에 따라 SOC와 PT기준으로 발생 빈도(대상자 수), 백분율 및 발생건수를 제시하고, 중증도별로 발생한 이상반응 및 약물이상반응에 대해서도 SOC기준으로 발생 빈도(대상자 수), 백분율 및 발생건수를 제시한다.
- 중대한 이상반응(약물이상반응)은 시험군에 따라 SOC기준으로 발생 빈도(대상자 수),

백분율 및 발생건수를 제시하고, 이상반응, 기간, 경과, 중증도, 결과, 인과관계에 대해 일람표로 요약한다.

- 또한 이상반응이 발생한 모든 시험대상자의 개별 목록(individual lists)을 제시한다. (투약군/군, 보고된 이상반응, 임상시험용 의약품 투여와 관련된 시작일시(start date/time) 및 종료일시(end date/time), 경과, 중증도(intensity), 중대함(seriousness) 및 임상시험용 의약품과의 관련성(relationship), 취해진 조치(action taken) 및 최종 결과(final outcome)를 포함), 시험의 중단을 초래한 이상반응은 별도의 목록으로 제시한다. 이상반응은 MedDRA ver. 23.1 (Korean)에 따른 선호 용어를 사용하여 요약한다.

13.4.1.2. 활력징후, 신체검사

- 각 시점에 측정된 수치 및 투약 전 검사결과로부터의 변화에 대하여 기술통계(도수(N), 중앙값, 평균, 표준편차(SD), 최소값 및 최대값)로 요약한다.

13.4.1.3. 임상실험실검사

- 양적 변수(예, CBC, Clinical chemistry)의 경우, 각 측정 시점에 검사 결과 및 투약 전 검사결과로부터의 변화를 기술통계(도수(N), 중앙값, 평균, 표준편차(SD), 최소값 및 최대값)로 요약한다. 정성 변수의 경우, 투약 전 검사결과와 투약 후 검사결과를 요약하여 기술한다.

14. 부작용을 포함한 안전성의 평가기준, 평가방법 및 보고방법

14.1. 안전성 관련 용어의 정의

14.1.1. 이상반응(adverse events)

- 이상반응(adverse event, AE)이란, 임상시험에 사용되는 의약품을 투여 받은 피험자에게서 발생한 바람직하지 않고 의도되지 않은 증후(sign), 증상(symptom), 질병을 말하며, 해당

임상시험약과에 반드시 인과관계를 가져야 하는 것은 아니다. 그러므로 이상반응은 시험약과 관련되는 것으로 간주되든 아니든, 바람직하지 않고 의도되지 않은 증후 (예를 들어, 임상적으로 의미 있는 임상병리 검사치 이상), 증상 또는 시험중인 의약품과의 인과관계와는 무관하게, 임상시험 중에 새로 나타나거나 악화된 질병, 증후, 증상 또는 임상적으로 의미 있는 임상병리 검사치 이상 등이 포함된다.

14.1.2. 이상약물반응(Adverse Drug Reaction, ADR)

- 이상약물반응 (Adverse Drug Reaction, ADR)이란, 임상시험에 사용되는 의약품의 임의의 용량에서 발생한, 모든 유해하고 의도되지 않은 반응으로서, 임상시험에 사용되는 의약품과의 인과관계를 배제할 수 없는 경우를 말한다.

14.1.3. 중대한 이상반응(Serious adverse events)

- 중대한 이상반응/이상약물반응(SAE)은 임상시험 중 발생한 이상반응 또는 이상약물반응 중에서 다음 각 항목에 해당하는 경우를 말한다.
  - 사망하거나 생명에 대한 위험이 발생한 경우
  - 입원할 필요가 있거나 입원 기간을 연장할 필요가 있는 경우
  - 영구적이거나 중대한 장애 및 기능 저하를 가져온 경우
  - 태아에게 기형 또는 이상이 발생한 경우

14.2. 이상반응의 평가

14.2.1. 이상반응의 중증도 평가기준

- 연구자는 시험기간 동안 보고된 각각의 이상반응 및 중대한 이상반응에 대한 중증도를 평가한다. 중증도는 MedDRA ver. 23.1 (Korean)에 의거하여 평가한다.

Grade	
1	Mild; asymptomatic or mild symptoms; clinical or diagnostic observations only; intervention not indicated.

2	Moderate; minimal, local or noninvasive intervention indicated; limiting age-appropriate instrumental ADL*
3	Severe or medically significant but not immediately life-threatening; hospitalization or prolongation of hospitalization indicated; disabling; limiting self care ADL**
4	Life-threatening consequences; urgent intervention indicated.
5	Death related to AE.

14.2.2. 이상반응의 인과관계 평가

- 이상반응 및 중대한 이상반응에 대한 임상시험용의약품과의 인과관계 평가 기준은 다음과 같이 판정한다.
- 1) Definitely related (명확히 관련이 있음)
  - 이 약을 투여하였다는 증거가 있는 경우
  - 이 약 투여와 다른 어떤이유보다 본제 투여에 의해 가장 개연성있게 설명되는 경우
  - 투여 중단으로 이상반응이 사라지는 경우
  - 재투여(rechallenge, 가능한 경우에만 실시) 결과가 양성인 경우
  - 이상반응이 이 약 또는 동일계열의 이 약에 대해 이미 알려져 있는 정보와 일관된 양상을 보이는 경우
- 2) Probably related (관련이 있다고 생각됨)
  - 이 약을 투여하였다는 증거가 있는 경우
  - 이 약 투여와 이상반응 발현의 시간적 순서가 타당한 경우
  - 이상반응이 다른 원인보다 이 약 투여에 의해 더욱 개연성 있게 설명되는 경우
  - 투여 중단으로 이상반응이 사라지는 경우
- 3) Possibly related (관련이 있을 가능성이 있음)
  - 이 약을 투여하였다는 증거가 있는 경우
  - 이 약 투여와 이상반응 발현의 시간적 순서가 타당한 경우
  - 이상반응이 다른 가능성있는 원인들과 같은 수준으로 본제에 기인한다고 판단되는 경우

- 투여중단으로(실시된 경우) 이상반응이 사라지는 경우

- 4) Probably not related (관련이 없다고 생각됨)
  - 이 약을 투여하였다는 증거가 없는 경우
  - 이상반응에 대해 보다 가능성 있는 다른 원인이 있는 경우
  - 투여 중단 결과(실시된 경우)가 음성이거나 모호한 경우
  - 5) Definitely not related (명확히 관련이 없다고 생각됨)
  - 이 약을 투여하였다는 증거가 없는 경우
  - 이상반응에 대해 가장 개연성 있게 설명되는 다른 어떤 이유가 있는 경우
  - 투여 중단 결과(실시된 경우)으로 이상반응이 소실되지 않는 경우
- 6) Unknown (알 수 없음)

14.3. 이상반응 및 중대한 이상반응에 대한 기록

- 연구자는 증례기록서(CRF)에 이상 반응 및 중대한 이상 반응과 관련된 모든 정보 즉,이상반응명, 발생일, 종료일, 강도, 임상시험용의약품과의 연관성, 결과, 치료여부, 중대한이상반응 여부에 대한 기록을 한다.

14.4. 이상반응의 보고

14.4.1. 이상반응의 보고

- 모든 이상반응 및 중대한 이상반응은 시험대상자가 자발적으로 보고했든, 질문을 통해 알게 되었든, 또는 이학적 검사나 실험실적 검사 또는 다른 방법으로 확인이 되었든, 시험대상자의 진료기록과 증례기록서(Case Report Form, 이하 “CRF”)에 기록한다.
- 각 이상반응에 대해 일반적으로 다음과 같은 내용을 기록한다: 이상반응의 정확한 특성/기술, 발현일, 소실일, 심한 정도/강도, 치료가 필요한 지의 여부, 그런 경우 치료나 조치의 내용, 그 결과(환자가 이상반응으로부터 회복되었는지 여부), 시험약과의 인과관계, 중대한 이상반응에 해당하는지 여부.

- 중대한 이상반응에 해당하는 경우에는 시험자는 매우 신속하게(보통 24시간이내) 의뢰자에게 보고해야 한다. 이는 1) 임상시험에서 환자의 안전을 지속적으로 확보하기 위해서이고, 2) 식약청의 보고요건을 충족하기 위해서이다. 시험자는 이상반응을 의뢰자에게 보고할 뿐만 아니라, 해당 임상시험심사위원회(Institutional Review Board, "IRB")의 요건에 따라 이를 IRB에도 보고한다. IRB는 어떤 것을 언제까지 보고하여야 하며, 보고 메카니즘이 어떠한 지에 대해 시험자에게 알려주어야 한다. 시험자는 또한 의뢰자로부터 해당 시험약에 대한 "안전성 관련 사항에 대한 정보"를 전달받을 수 있다. 이러한 안전성 정보를 받는 경우에는 이를 IRB에 제출한다.

14.4.2. 중대한 이상약물반응의 보고

- 의뢰자는 기타 관련된 시험자, 심사위원회 및 식품의약품안전처장에게 중대하고 예상하지 못한 모든 이상약물반응을 다음 각호의 1에서 정한 기간내에 신속히 보고하여야 한다.
- 사망을 초래하거나 생명을 위협하는 경우에는 의뢰자가 이 사실을 보고받거나 알게 된 날로부터 7일 이내. 다만, 이 경우 상세한 정보를 최초 보고일로부터 8일 이내에 추가로 보고하여야 한다.
- 다른 모든 중대하고 예상하지 못한 이상약물반응의 경우에는 의뢰자가 이 사실을 보고받거나 알게 된 날로부터 15일 이내
- 의뢰자는 제1항의 보고와 관련하여 추가적인 안전성 정보를 주기적으로 해당 이상약물반응이 종결(해당 이상약물반응의 소실 또는 추적조사의 불가 등)될 때까지 보고하여야 한다.

14.5. 이상반응/이상약물반응의 추적관찰

- 임상실험실 검사 등의 결과에 대한 정상/이상의 기준과 시험책임자가 치료 전과 후의 변화가 임상적으로 의미 있게 피험자의 건강에 악영향을 끼친 것으로 판단하는 경우

이상반응으로 판정한다. 각 임상검사치의 정상/이상은 개별 피험자에서 판정하며 필요한 경우 통계적 검증을 할 수 있다. 임상검사치 이상에 대하여 그 정도를 기재하고 시험약과의 관련성을 판정한다. 임상검사치에 어떤 이상이 있다면 환자를 추적관찰하고 임상검사를 반복하여 검사치가 정상으로 회복되었는지를 기록하여야 한다.

14.6. 임상시험 후 시험대상자의 진료 및 치료기준

- 본 임상시험이 완료 또는 중지 탈락된 대상자는 진료 후 필요에 따라 적절한 조치를 받도록 한다. 임상시험 담당자에 의해 처치가 필요하다고 판단되는 이상반응이 발생한 경우 '피해자 보상에 관한 규약'에 의거하여 적절한 조치를 받도록 한다.

15. 연구윤리

15.1. 임상시험심사위원회(IRB)

- 임상시험을 시작하기 전에, 시험자는 임상시험자자료집, 임상시험계획서, 대상자 설명서 및 동의서, 대상자 확보방법(광고 등을 포함), 그 밖에 대상자에게 문서 형태로 제공되는 각종 정보에 대하여 IRB의 심사를 받아야 한다. IRB는 시험 실시에 대한 심의 결과를 임상시험 시작 전에 시험자에게 문서로 전달한다.
- 시험책임자는 임상시험계획서 및 개정 또는 변경사항에 대하여 IRB의 승인을 받은 후 임상시험을 실시해야 한다. 또한 중대한 이상반응을 포함하여 환자의 안전성이나 임상시험의 지속적인 실시에 영향을 줄 수 있는 사건, 특히 안전성과 관련된 변화를 반드시 IRB에 보고해야 한다. 해당 IRB 기준에 따라 필요시 진행상황에 대한 보고서를 IRB에 제출하고, 임상시험 종료 시에는 이를 IRB에 알려야 한다.

15.2. 시험대상자 동의

- 임의의 시험 절차(스크리닝을 포함하여)의 시행, 시험 관련 검사의 시작 및 임상시험용 의약품의 투여 이전에 시험대상자 동의가 성립되어야 한다. 서명과 날짜가 기입된

시험대상자 동의서를 의약품 임상시험 관리기준(KGCP)에 의거하여 각 시험대상자로부터 취득한다. 시험자는 작성된 시험대상자 동의서를 시험 기록의 일부로서 보관한다.

- 시험대상자 동의서는 적절히 변경될 수 있다. (예, 시험계획서 변경 또는 유의한 새로운 안전성 정보로 인하여) 동의서가 개정되는 경우, 시험자는 해당 의학연구윤리심의위원회(IRB)의 승인 이전에 시험자가 변경된 동의서를 검토하고 승인하였는지, 시험에 참여하였고 현재 참여하고 있는 모든 시험대상자가 서명하였는지 확인하여야 할 책임이 있다.
- 시험 스크리닝 이전에 서명되고 날짜가 기입된 시험대상자 동의서에 대한 기록은 시험대상자 동의가 취득된 시점에 증례기록서(CRF) 및 근거문서에 반드시 명시한다.

15.3. 비밀보장

- 대상자의 신원을 파악할 수 있는 모든 기록은 비밀을 유지해야 한다. 증례기록서 등 임상시험에 관련된 모든 서류에는 대상자의 사생활 보호 및 기록의 비밀 보장을 위하여 대상자의 이름이 아닌 대상자 식별코드(대상자 번호, 이니셜)로 기록하고 구분하며, 의료기록 중 문서(병리학적 보고서, 영상기록 등)에 대상자 이름이 있을 경우에는 이를 삭제하여 보관한다. 임상시험의 결과가 출판될 경우에도 대상자의 신원은 비밀 상태로 유지될 것이며, 시험대상자의 개별적인 데이터를 출판 또는 보고해야 할 경우에는 대상자의 번호 또는 이니셜만을 기록할 것이다.
- 임상시험 모니터 요원 및 점검자, 그리고 식품의약품안전처와 임상시험심사위원회에서는 수집된 정보의 확인을 목적으로 대상자의 의료 기록을 열람할 수 있다. 이 때 노출되는 대상자의 정보는 철저한 비밀 유지하에 다루어질 것이며, 시험자는 대상자에게 이러한 사실을 알려줄 것이다.

15.4. 시험대상자 안전보호에 관한 대책

- 임상시험 실시기관은 본 임상시험의 실시에 필요한 설비와 전문인력을 갖추고,

임상시험을 적절하게 실시할 수 있도록 준비에 완벽을 기해야 한다.

- 시험자는 대상자를 임상시험에 등록하기 전에 각 대상자들의 건강상태를 확인하여 임상시험에 참여하기에 적합한지 철저히 확인하여야 한다. 또한 시험자는 임상시험계획서 및 임상시험용 의약품에 대하여 충분히 숙지하고 계획서에 따라 임상시험을 실시한다. 대상자의 안전을 보장하기 위해 최선을 다하며, 임상시험으로 인한 이상반응이 발생한 경우, 시험자는 즉시 필요한 검사 및 치료를 받을 수 있도록 조치하여야 한다. 또한 필요한 경우 해당 이상반응이 소실되거나 추적 조사가 불가능하게 될 때까지 관찰하여야 한다.
- 임상시험 기간 중 대상자가 이상반응으로 인해 처치를 받기 원하거나 시험자가 의학적 처치가 필요하다고 판단하는 경우에는 즉시 시험기관에 내원하여 관련 검사를 받아야 한다. 시험자가 이상반응 및 검사 결과를 평가하여 시험을 중지하여야 한다고 판단하거나, 대상자가 시험 중단을 원하는 경우 시험자는 임상시험 종료에 따른 절차를 신속하게 진행하고 대상자에게 해당 증상을 완화시킬 수 있는 치료를 적극적으로 실시해야 한다.

16. 기타 임상시험을 안전하고 과학적으로 실시하기 위하여 필요한 사항

임상시험을 실시함에 있어 KGCP 및 Helsinki 선언의 근본정신을 준수하여 윤리적이고 과학적인 배려 하에 연구를 실시하도록 한다. 본 임상시험계획서에서 제시하지 않는 내용은 KGCP 와 관련 법령, 고시 및 Helsinki 선언의 내용에 따른다.

16.1. 증례기록서 (CRFs)

- 시험자는 각 시험 참여자에 대한 모든 관찰 사항 및 관련 기타 자료를 기록하도록 고안된 적절하고 정확한 증례 기록을 준비하고 유지하여야 한다. 이는 본 시험계획서에 명시된 임상시험용 의약품 수불확인의 정확한 근거 문서를 포함한다. 시험자는 시험 이후 시험대상자에게 연락하기 위하여 필요한 시험대상자 식별 코드 목록을 보존하여야 한다.

- 이 목록은 시험 완료 이후 시험자의 소속 기관에서 기밀 사항으로 유지할 것이다. 증례기록서는 스크리닝에서 제외된 시험대상자를 포함하여 시험에 등록된 각 시험대상자에 대하여 작성한다. 본 시험에서 증례기록서에 기록된 모든 정보는 시험대상자의 근거 문서와 일관성이 유지되어야 한다.
- 증례기록서에는 기록해야 할 자료가 발생할 때 즉시 기록한다. 만약 증례 종료시까지 기록되지 않은 경우 적절한 누락사유를 기록하여야 한다. e-CRF 상의 수정이 필요한 경우 e-CRF의 System query (자동)와 Manual query (수동)를 사용하여 수정자료, 수정자 성명 및 서명, 수정사유, 수정일이 기록되도록 한다.

16.2. 전산시스템의 사용

- 임상시험의 자료는 자료처리의 편의성과 임상시험 질관리를 위하여 종이CRF 대신 e-CRF(REDcap, Vanderbilt 대학 개발, https://www.project-redcap.org/)를 사용하여 입력, 관리하며, 모든 서명은 전자서명으로 대체한다(근거: <2015년 임상시험 관련 자주 묻는 질의응답집>(식약처, 2015)).

16.3. 자료의 질 보장 및 자료보안

- 임상 시험 시작 전에 연구자가 참여한 개시모임을 개최한다. 이 모임에서 연구계획서, 연구 수행, 증례기록서 완성, 샘플 수집 및 전처리 방법 등에 관한 자세한 논의를 한다. 모니터 요원은 모니터링 계획에 따라 모니터링을 한다.
- 증례기록서에 기록된 모든 자료를 임상시험 코디네이터와 임상시험 모니터요원이 이중 확인하도록 한다. 이상이 발견된다면 근거문서와 비교 검토한다. 증례기록서에 입력된 자료를 시험기관의 SOP 와 자료 관리 계획 (data management plan)에 따라 이중 확인하도록 한다. 자료 입력 완료가 확인되면, 데이터베이스 시스템을 lock 한다.
- 해결되지 않은 쿼리에 대한 쿼리 리스트는 임상시험 모니터 요원을 통해 시험자와 함께 해결하도록 한다. 데이터베이스는 서명된 쿼리해결에 기초하여 수정한다.

- 연구를 통하여 얻어진 근거문서, 증례기록서 등의 모든 문서에 대해서는 보안이 유지되어야 하며, 연구자는 의뢰자의 동의 없이 이러한 정보들을 공개해서는 안 된다.
- 시험대상자의 익명성이 보장되어야 하므로, 모든 문서에 시험대상자 이름 대신 시험대상자 번호나 이니셜을 사용한다. 시험대상자를 식별할 수 있는 문서도 연구자에 의해 보안이 유지되어야 한다.

16.4. 기록의 관리 및 보관

- 임상연구 실시와 관련된 각종 자료 및 기록을 경희대학교병원에서 보존하고 보안을 유지하도록 한다. 임상연구 관련 문서에 대한 관리 및 보관 책임자는 시험책임자로 하고 접근자는 시험담당자로 제한한다. 임상시험결과보고서 작성 완료 이후에는 생명윤리법에 따라 동의서 및 기타 임상연구 관련 문서는 3년간 보존하도록 한다.

16.5. 임상시험 실시기관의 모니터링

- 시험대상자의 권리와 복지 보호, 보고된 임상시험 관련 자료가 근거문서와 대조하여 정확하고, 완전하며, 검증이 가능한지 여부 확인, 임상시험이 승인된 계획서, 의약품임상시험관리기준 및 시행규칙 제 28조의 규정에 따라 수행되는 지의 여부 확인을 위하여 모니터링을 실시한다.
- 임상시험에 대한 모니터링은 업무를 위탁 받은 경희대학교한방병원 한의약임상시험센터 모니터요원의 정기적인 시험자 방문과 전화를 통해서 이루어 질 것이다. 방문 시 모니터는 기본적으로 환자기록 원본, 약물 관리 기록, 자료 보관(연구 파일)등을 확인하며, 시험대상자의 고유코드별로 할당된 무작위번호의 눈가림이 유지됨을 확인해야 한다. 또한, 모니터는 임상시험 진행과정을 잘 살피고, 문제가 있을 경우 시험자와 상의한다.
- 이들 방문의 적절한 시간은 시험자와 모니터 간 협의하여 배분하여야 한다. 시험자는 또한 의약품 임상시험 관리기준에 정의된 것과 같이, 모니터가 증례기록서에 기입된 자료들을 확인할 수 있는 시험대상자의 원자료들(source documents: 병원 또는 개인

차트, 실험실 결과기록, 예약 기록 등)을 볼 수 있도록 해주어야 한다.

17. 참고문헌

- Masahiro UMINO, et al. Effect of Byakko-ka-Ninjin-to on Xerostomia in elderly patients -Analysis of the relationship between improvement of subjective symptom and Kampo diagnosis-. Kampo Medicine. 1994;45(1):107-113.)
- Shuji YAKUBO, et al. The Effects of Byakko-ka-ninjin-to on Patients in whom Thirst has been induced by Disopyramide Phosphate. Kampo Medicine. 1995;46(3):433-438.)
- Masaru Sakaguchi, et al. Effects of Byakko-ka-ninjin-to on salivary secretion and bladder function in rats. J Ethnopharmacol. 2005 Nov 14;102(2):164-9.
- A Niijima, et al. Effect of byakko-ka-ninjin-to on the efferent activity of the autonomic nerve fibers innervating the sublingual gland of the rat. J Auton Nerv Syst. 1997 Mar 19;63(1-2):46-50.
- -Vivino FB, et al. Pilocarpine tablets for the treatment of dry mouth and dry eye symptoms in patients with Sjögren syndrome: a randomized, placebo-controlled, fixed-dose, multicenter trial. P92-01 Study Group. Arch Intern Med. 1999. PMID: 9927101 Clinical Trial.
