# Supplementary material for: Efficacy of combined administration of Baekhogainsam-Tang and low-dose pilocarpine on frequent intractable xerostomia: Study protocol for a randomized controlled trial
Source: PLoS One. 2024 Nov 11;19(11):e0307557. doi: 10.1371/journal.pone.0307557 (PMC11554038; doi:10.1371/journal.pone.0307557)
Supplement: S3 Appendix — (PDF) [file pone.0307557.s003.pdf]

Clinical Study Protocol

Effect of Combined Administration of Pilocarpine and Herbal medicine (Baekhogainsam-Tang Ext. Granule) on frequent intractable dry mouth: A randomized, open-label, parallel, multi-center trial

Protocol No: KHMC-Xerostomia-01

Clinical trial stage	Intervention study
Principal investigator	Young-Gyu Eun
Clinical research center	Kyung Hee University Medical Center
Version number/date	Version 2.6 / 2023.10.19

Version 2.6

Protocol Version History

No	Version No.	Version Date	Modification
1	1.0	2020.10.07	None
2	2.0	2021.01.21	Modification of inclusion criteria (excluding senile dry mouth syndrome)<br>Modification of exclusion criteria (related to prohibited drugs, side effects, and male contraception)<br>Add a prohibited drug<br>Add a screening clinical laboratory test<br>Add inspection item for laboratory tests to the assessment of adverse reactions
3	2.1	2021.07.01	Protocol No. error correction<br>OHIP-14 Questionnaire typing error correction
4	2.2	2021.07.27	Correction of typing error in prescription days
5	2.3	2021.08.15	Correction of typing error in drug formulation
6	2.5	2022.02.16	Modification of exclusion criteria<br>Addition of one institution (Kyung Hee University Hospital at Gangdong)<br>Adjusting the number of subjects and method of assigning test subjects' number
7	2.6	2023.11.06	Change in expected dropout rate<br>Change in the number of test subjects<br>Extension of patient recruitment period

Summary of the protocol

Title	Effect of Combined Administration of Pilocarpine and Herbal medicine (Baekhogainsam-Tang Ext. Granule) on frequent intractable dry mouth: A randomized, open-label, parallel, multi-center trial
Trial stage	Intervention study
Objective	To ascertain whether combined administration of BIT herbal medicine and low-dose pilocarpine can demonstrate both non-inferior efficacy and fewer side effects in patients with frequent intractable xerostomia (patients who underwent radiation therapy to the head and neck or with Sjogren's syndrome) than the administration of only high-dose pilocarpine.
Principal investigator	Young-Gyu Eun / Department of Otolaryngology-Head and Neck Surgery, Kyung Hee University School of Medicine, Kyung Hee University Medical Center
Co-investigator	Junhee Lee / Department of Sasang Constitutional Medicine, Kyung Hee University College of Korean Medicine, Kyung Hee University Korean Medicine Hospital
Clinical pharmacist	Kyung Won Yoon / Pharmaceutical Headquarters, Kyung Hee University Medical Center, Pharmacist<br>Hye Jung Hong / Pharmaceutical Headquarters, Kyung Hee University Medical Center, Pharmacist
Co-clinical trial institutions	Kyung Hee University Medical Center<br>Inha University Hospital / Ji Won Kim<br>Myongji Hospital / Su Young Jung<br>Kyung Hee University Hospital at Gangdong / Young Chan Lee
Participants	Patients with frequent intractable xerostomia (patients who underwent radiation therapy to the head and neck or with Sjogren's syndrome)
Inclusion / Exclusion criteria	● **Inclusion criteria**<br>1. One or more of the following<br>• Patients who undergone radiation therapy to the head and neck<br>• Patients with Sjogren's syndrome with both followings<br>- positive anti-Ro/SS-A antibody reaction<br>- Saliva flow rate (Unstimulated) ≤ 0.1mL/min<br>2. unstimulated SFR ≤ 0.25 ml/min<br>3. VAS score for dry mouth over the past one month ≥ 4<br>4. age >19 years

5. compliance with all written informed consent

- **Exclusion criteria**

1. Patients with the following past medical history
   - heart failure
   - medication-refractory hypertension (systolic blood pressure ≥ 160 mmHg or diastolic blood pressure ≥ 100 mmHg)
   - bronchial asthma
   - arrhythmia accompanied by clinical symptoms (fainting, palpitation, or shortness of breath) or requiring treatments (sinus failure, atrial conduction block, atrial fibrillation, atrial junction dependent tachycardia, ventricular tachycardia)
   - coronary artery disease
   - asthma, chronic obstructive pulmonary diseases, chronic bronchitis
   - inborn errors of amino acid metabolism
   - hepatic encephalopathy
   - ophthalmic diseases (such as narrow angle glaucoma, peripheral retinopathy, and iritis)
2. Patients taking following medications are also excluded: coumadin, heparin, warfarin, aspirin, anticholinergics, first-generation antihistamines, antidepressants with anticholinergic effects, monoamine oxidase inhibitors, diuretics, mineralocorticoids, β-adrenaline antagonist, or digitalis; patients taking low-dose aspirin or β-adrenaline antagonist can participate in the protocol based on the cardiologist's opinion.
3. Clinical laboratory test results falling within the following ranges (within 7 days prior to the start of clinical trial drug administration)
   - hemoglobin < 8.0 g/dL
   - aspartate transaminase (AST) or alanine transaminase (ALT) > 3 times the upper limit of each hospital standard
   - total bilirubin > 2 times the upper limit of each hospital standard
   - creatinine >1.5 times the upper limit of each hospital standard, or creatinine clearance ≤ 60 mL/min
4. pregnancy or lactation, disagreement with proper contraception
5. Failure to use appropriate contraception methods (male participants), participants who did not agree to contraception for 4 weeks after the end of administration of the trial drug (both male and female)

	6. galactose intolerance, Lapp lactase deficiency, or glucose-galactose malabsorption<br>7. difficulty in oral administration of a clinical trial drug<br>8. significant hypersensitivity to Baekhogainsam-Tang or components<br>9. significant hypersensitivity to pilocarpine<br>10. Participants who is deemed unsuitable for participation in clinical trials at the discretion of the investigator due to various reasons including screening test results<br>11. cognitive disorder; inability to read and write.
Experimental and control group	1. Experimental group (Combined Administration of Pilocarpine and Herbal medicine)<br>: receive both two medications<br>• pilocarpine hydrochloride half dose (2.5 mg)<br>• Baekhogainsam-Tang herbal granules<br>2. Control group (Conventional Care, CPC)<br>: receive only pilocarpine<br>• pilocarpine hydrochloride 5 mg dose

<table>
<tr><td>Clinical trial drug</td><td>

- **Trial drug 1**

<table>
<tr><td>Product name</td><td>Pilogen Tab.</td></tr>
<tr><td>Manufacturer</td><td>CTC Bio, Inc.
(Seoul, Republic of Korea)</td></tr>
<tr><td>Approval date</td><td>2008-Jun-18</td></tr>
<tr><td>Formulation</td><td>white circular film coating tablet</td></tr>
<tr><td>Components / Amounts</td><td>Pilocarpine Hydrochloride 5 mg</td></tr>
<tr><td>Authorized efficacy effect</td><td>The treatment of symptoms of dry mouth from salivary gland dysfunction caused by radiotherapy for head and neck cancer.
The treatment of symptoms of dry mouth or dry eye in patients with Sjogren's syndrome.</td></tr>
<tr><td>Dosage & Administration</td><td>Experimental group: half table (2.5 mg) four times a day, taken orally for 12 weeks
Control group: one tablet (5 mg) four times a day, taken orally for 12 weeks
(four times: after each meal and before bedtime)</td></tr>
<tr><td>Storage</td><td>Airtight container, room temperature (1-30℃)</td></tr>
</table>

- **Trial drug 2**

<table>
<tr><td>Product name</td><td>Baekhogainsam-tang Ext. Granule</td></tr>
<tr><td>Manufacturer</td><td>Hanpoong Pharmaceutical & Food Co., Ltd.
(Jeonju, Republic of Korea)</td></tr>
<tr><td>Approval date</td><td>1979-Sep-18</td></tr>
<tr><td>Formulation</td><td>grayish-brown granule</td></tr>
<tr><td>Components / Amounts</td><td>Single dose (4 g / dose)
<table>
<tr><td>Application category</td><td>Scientific name</td><td>stan dard</td><td>Amount (mg)</td><td>Active substance capacity</td></tr>
<tr><td>Main component</td><td>Anemarrhena Rhizome</td><td>KP</td><td>2,000</td><td></td></tr>
<tr><td>Main component</td><td>Gypsum</td><td>KHP</td><td>5,330</td><td></td></tr>
</table>
</td></tr>
</table>

</td></tr>
</table>

	Main component	Licorice	KP	660	13.2 mg as glycyrrhizinate
	Main component	Oryzae Semen	KHP	3,300	
	Main component	Ginseng	KP	1,000	2.0 mg as ginsenoside Rb1
		Soft extract		830	
	Diluent	Lactose Hydrate	KP	2,260	
	Excipients	Corn Starch	KP	Quantum sufficit	
	Excipients	Hydroxypropyl Cellulose	KP	Quantum sufficit	
	Excipients	Sucrose Fatty Acid Ester	KP	Quantum sufficit	
	Excipients	Magnesium Stearate	KP	Quantum sufficit	
	Excipients	Light Anhydrous Silicate	KP	Quantum sufficit	
		Total		4,000	100%
Authorized efficacy effect	The treatment of symptoms such as flushing, thirst, and sweating				
Dosage & Administration	Experimental group: One packet (4 g) three times a day, taken orally for 12 weeks<br>(three times: before each meal)<br>Control group: None				
Storage	Airtight container, room temperature (1-30℃)				

Sample size, basis for calculation

- Sample size

- 120

	Experimental group	Control group
	Pilocarpine half tablet + Baekhogainsam-tang	Pilocarpine one tablet
Sample size	60	60

- Sample size by clinical trial institution

	Kyung Hee University Medical Center	Inha University College of Medicine	Myongji Hospital	Kyung Hee University Hospital at Gangdong	Total
Experimental group	32	16	2	10	60
Control group	32	16	2	10	60
Total	64	32	4	20	120

- Competitive recruitment

- Sample size in Kyung Hee University Medical Center: 64 (experimental 32, control 32)

- Basis for calculation of sample size

- The aim of this prospective, randomized, open-label, parallel-group, multi-center, and controlled trial is to ascertain whether combined administration of BIT herbal medicine and low-dose pilocarpine can demonstrate both non-inferior efficacy and fewer side effects in patients with frequent intractable xerostomia (patients who underwent radiation therapy to the head and neck or with Sjogren's syndrome) than the administration of only high-dose pilocarpine.

- The assumptions for calculating the valid sample size are as follows.

- One-sided Z test (Non-inferiority test)

	- Level of significance (α) = 0.05 (Considering this is an intervention study)<br>- Type 2 error (β) = 0.1 (Power of the test = 90%)<br>- Experimental group: Control group = 1 : 1 (λ=1)<br>- Primary outcome: unstimulated salivary flow rate (SFR) after 12 weeks of treatment<br>- σ = 0.35 / d (non-inferiority limit) = 0.21<br>- In the previous study, the mean ± standard deviation (SD) of unstimulated SFR for 5 mg pilocarpine therapy vs placebo group after 12 weeks of medication, respectively, were 0.38 ± 0.48 vs 0.17 ± 0.13 mL/min (p <0.001). Based on these results, the SD was calculated to be 0.35<br>- In consideration of the exploratory aspects of the clinical trial, the point estimation (μclH - μplH; 0.38−0.17=0.21) after 12 weeks of administration of 5 mg pilocarpine and placebo was set as the non-inferiority limit.<br>- Reference: Vivino FB, et al. Pilocarpine tablets for the treatment of dry mouth and dry eye symptoms in patients with Sjögren syndrome: a randomized, placebo-controlled, fixed-dose, multicenter trial. P92-01 Study Group. Arch Intern Med. 1999. PMID: 9927101 Clinical Trial.<br>- H01 : μc - μt > 0.21<br>H11 : μc - μt ≤ 0.21<br>μc = SFR after 12 weeks of administration of only pilocarpine one tablet<br>μt = SFR after 12 weeks of combined administration of Baekhogainsam-tang herbal medicine and low-dose pilocarpine<br>- Nc=Nt= $\frac{(1+\lambda)\sigma^2(Z_\alpha+Z_\beta)}{\lambda d^2}$ ≒48<br>- Assuming a 20% dropout rate, we calculated the sample size as 120 participants (60 in each group).
Trial design	Eligible xerostomia participants with Sjogren’s syndrome or those who have received radiotherapy for head and neck cancer will be randomly allocated to either the experimental or control group after screening based on the inclusion and exclusion criteria. The patients will receive treatment for 12 weeks. The aim of this prospective, randomized, open-label, parallel-group, multi-center, and controlled trial is to assess

	and compare the efficacy and safety in each group.
Combination therapy	None
Permitted drug in combination	Drugs other than prohibited drugs in combination are allowed, but they are reviewed by principal investigator and sub-investigators during screening and clinical trials period.
Prohibited drug in combination	Coumadin, heparin, warfarin, aspirin, anticholinergics, first-generation antihistamines, antidepressants with anticholinergic effects, monoamine oxidase inhibitors, diuretics, mineralocorticoids, β-adrenaline antagonist, or digitalis<br>Patients taking low-dose aspirin or β-adrenaline antagonist can participate in the protocol based on the cardiologist's opinion.
Primary and secondary outcomes	1. Primary outcome<br>- Unstimulated SFR after 12 weeks of treatment<br>- Participants will abstain from food intake for at least one hour. The participants will need to rinse their mouth with water, held it open, and gather saliva for 5 min. SFR (ml/min) is calculated by dividing the measured amount of saliva (ml) by time (5 min).<br>2. Secondary outcomes<br>- Stimulated SFR after 12 weeks of medication<br>- Differences and mean percentage changes in unstimulated and stimulated SFR from baseline (week 0) to the end of the trial (week 12)<br>- To determine the stimulated SFR, the participants will be encouraged to suck sugar-free lemon candy. The procedure for measuring the stimulated salivary flow is the same as that for measuring the unstimulated salivary flow.<br>- Differences and mean percentage change in the visual analogue scale (VAS) and 14-item Oral Health Impact Profile (OHIP-14), and from baseline

	(week 0) to the end of the trial (week 12).<br>- Differences and mean percentage changes in the uptake ratio (UR), maximum accumulation (MA), and maximum secretion (MS) on salivary scintigraphy from baseline (week 0) to the end of the trial (week 12).
Safety monitoring	Adverse events, vital signs, physical examinations, and clinical laboratory tests
Statistical methods	**1. Patients group for data analysis**<br>- Demographic information will be assessed for all participants.<br>- Safety outcomes will be analyzed in the intention-to-treat (ITT) population.<br>- Efficacy outcomes will be analyzed for both the per-protocol (PP) and ITT populations. In case of discrepancies between the efficacy outcomes of PP and ITT populations, both set of results will be presented and compared, with the PP population considered the main analysis and the ITT population as supplementary analysis.<br>- The ITT population consists of participants who received at least one dose of the clinical trial drugs and underwent efficacy evaluation more than once during the treatment period. In cases of missing values due to dropouts or discontinuation before the clinical trial ends, missing data from dropout participants will be imputed using the last observation carried forward analysis.<br>- The PP population is a subset of the ITT population. It includes participants who consumed >70% of the prescribed doses of clinical study drugs and completed follow-up visits and corresponding outcome measurements (weeks 4, 8, and 12) during the 12-weeks treatment periods. Participants falling under a 'significant violation of clinical trial plan' will be excluded

from the PP population.

2. Statistical analysis

- Data will be presented as the mean ± SD for continuous data or frequencies for categorical data. To confirm the validity of the random allocation, demographic and baseline values between the experimental and control groups will be compared and evaluated. We will use the independent t-test or Mann-Whitney U test for continuous outcome measures and the chi-square test, Fisher's exact test, or Cochran-Mantel-Haenszel Method for categorical outcome measures.
- A 95% confidence interval (95% CI) will be used to analyze primary outcomes. We will calculate the 95% CI of the mean difference in the unstimulated SFR between the two groups after 12 weeks of medication. If the lower end of the 95% CI of the mean difference is greater than the non-inferiority limit (−0.21), it will be concluded that the experimental group is not inferior to the control group. For further analysis, if the SFR between the two groups satisfy the normality test, a t-test will be performed to compare the two groups in terms of the primary outcomes; otherwise, the Mann-Whitney U test will be performed. If there is a significant difference in baseline values between the two groups, repeated-measures analysis of covariance (ANCOVA) will be performed.
- To analyze the secondary outcomes, in the case of continuous outcome measures, a normality test for the distribution of data will be initially performed. In the case of non-normally distributed data, the data will be transformed to a normal distribution using the log-transformation or square root transformation methods. The analysis will the proceed using

	either parametric or non-parametric methods. Specifically, an independent T test or Mann-Whitney U test will be performed to compare values between the two groups. The Paired T test or Wilcoxon signed-rank test will be performed to compare intragroup continuous values. The group and time interaction effect of repeated-measures data will be analyzed through repeated-measures analysis of variance (ANOVA) or ANCOVA, or through the GEE model test based on the nature of the data. The level of significance will be set at $p < 0.05$.

❖ Summary schedule of the clinical trial

Timepoint / Assessment	Screening Period	Treatment period											
	-1w – 0w	1w	2w	3w	4w	5w	6w	7w	8w	9w	10w	11w	12w
	V1 (-1w~0d)	V2 (0w)			V3 (4w±2d)				V4 (8w±2d)				V5 (12w±2d)
Informed consent	●												
Demographics	●												
Medical examination	●												
Physical examination	●	●			●				●				●
Vital signs	●	●			●				●				●
Blood tests	●				●				●				●
12 lead ECG	●												
Random allocation	●												
Pilocarpine[1)]		●	●	●	●	●	●	●	●	●	●	●	●
Baekhogainsam-Tang[2)]		●	●	●	●	●	●	●	●	●	●	●	●
OHIP-14		●			●				●				●
EQ-5D-5L		●			●				●				●
VAS	●				●				●				●
Salivary flow test (SFR)	●				●				●				●
Salivary scintigraphy		●											●
Monitoring adverse events		●			●				●				●
Evaluating concomitant medication		●			●				●				●

1) Pilocarpine: Experimental group = half table (2.5 mg) four times a day / Control group = one tablet (5 mg) four times a day

2) Baekhogainsam-Tang: Experimental group = one packet (4 g) three times a day / Control group = none

※ Visits one and two can be performed simultaneously, after obtaining written informed consent.

※ If the test items included in the screening test have already been performed in the same hospital, the results of the test is recognized as a screening test up to 30 days after the test.

Table of Contents

❖ **Summary schedule of the clinical trial** 14

1. The title and stage of the study 20

1.1. Trial title 20

1.2. Trial stage 20

2. Trial institution 20

2.1. Clinical trial institution 20

2.2. Co-clinical trial institutions 20

3. Principal investigator, Co-investigator, and clinical pharmacist 20

3.1. Principal investigator 20

3.2. Co-investigator 20

3.3. Research assistants 21

3.4. Clinical pharmacist 21

4. The objective and background of the study 21

4.1. Objective 21

4.2. Backgrounds 21

4.2.1. Background for diseases 21

4.2.2. Backgrounds for conventional treatments 22

4.3. Backgrounds on clinical trial drugs 23

4.4. Necessity of the clinical trial 27

5. The clinical trial drug information 29

5.1. Overview of clinical trial drugs 29

5.1.1. Pilocarpine 29

5.1.2. Baekhogainsam-Tang 29

5.2. Packing and labeling of clinical trial drugs 31

5.3. Methods of management and storage of clinical trial drugs 31

6. The duration of the study 32

7. Test subjects 32

8. Inclusion criteria, exclusion criteria, sample size, and basis for calculation 32

8.1. Inclusion criteria 32

8.2. Exclusion criteria **33**

8.3. Sample size **34**

8.4. Basis for sample size calculation **35**

9. Trial design **36**

9.1. Trial design **36**

9.2. Trial schedule **37**

9.3. Dosage and administration of the trial drugs **37**

9.4. Combination therapy **38**

9.5. Randmo allocation **38**

9.6. Methods of registration and randomization **38**

9.7. Managements of random allocation **38**

9.8. Test subject number **39**

9.9. Clinical trial schedule **39**

9.9.1. Screening visit 39

9.9.2. Visit 2~5 (Week 0~12) 40

9.10. First aid medicine **41**

9.11. Prohibited drug in combination **41**

9.12. Permitted drug in combination **41**

10. Observation items and clinical examination items **41**

10.1. Screening evaluation items **41**

10.1.1. Evaluation of general items 41

10.1.2. Vital signs, physical examination 42

10.1.3. 12-lead ECG 42

10.1.4. Clinical laboratory examination 42

10.1.5. Salivary flow rate (SFR) test 43

10.1.6. Evaluation of visual analog scale (VAS) for xerostomia 43

10.2. Evaluation of efficacy **43**

10.2.1. Salivary flow rate (SFR) test 43

10.2.2. Evaluation of VAS for xerostomia 43

10.2.3. Salivary scintigraphy 44

10.2.4. OHIP-14 (The 14-item Oral Health Impact Profile) Questionnaire 44

10.3. Evaluation of safety **45**

10.3.1. Adverse events evaluation 45
10.3.2. Vital signs 46
10.3.3. Clinical laboratory examination 46

10.4. Econimic assessment 47

11. Predictive side effects and precautions 48

11.1. Pilogen 48
11.1.1. Predictive side effects 48
11.1.2. Precautions 50
11.1.3. Treatment when overdosing 54
11.1.4. Others 54
11.1.5. Storage and handling precautions 55

11.2. Baekhogainsam-Tang 55
11.2.1. Predictive side effects 55
11.2.2. Precautions 56

12. Suspension and withdrawal criteria 57

12.1. Suspension and withdrawal criteria 57

12.2. Management in case of suspension and withdrawal 58

12.3. Management of violation of clinical trial plan 58

13. Evaluation criteria, methods, and statistical analysis 59

13.1. Patient group for data analysis 59
13.1.1. Demographic information 59
13.1.2. Safety outcomes 59
13.1.3. Efficacy outcomes 59

13.2. Demographic data 60

13.3. Efficacy assessment 60
13.3.1. Primary outcome efficacy assessment 60
13.3.2. Secondary outcomes efficacy assessment 61

13.4. Safety assessment 62

14. Safety evaluation criteria and method, and reporting method 64

14.1. Definition of safety-related terms 64
14.1.1. Adverse events (AEs) 64
14.1.2. Adverse Drug Reaction (ADR) 64
14.1.3. Serious adverse events (SAEs) 64

14.2. Evaluation of adverse events (AEs) 65
14.2.1. Criteria for evaluating the severity of AEs 65
14.2.2. Evaluation of causality of AEs 65
14.3. Records of AEs and SAEs 66
14.4. Report of AEs 67
14.4.1. Report of AEs 67
14.4.2. Report of SAEs 67
14.5. Follow-up of AEs and adverse drug reactions (ADRs) 68
14.6. Management and treatment of test subjects after clinical trial 68
15. Research ethics 68
15.1. Institutional Review Board (IRB) 68
15.2. Informed consent 69
15.3. Confidentiality 70
15.4. Measures for safety protection of test subjects 70
16. Other items necessary to conduct clinical trials safely and scientifically 71
16.1. Case report forms (CRFs) 71
16.2. Use of computer systems 72
16.3. Ensuring data quality and data security 72
16.4. Management and storage of records 73
16.5. Monitoring of clinical trial institutions 73
17. References 74

1. The title and stage of the study

1.1. Trial title

- Effect of Effect of Combined Administration of Pilocarpine and Herbal medicine (Baekhogainsam-Tang Ext. Granule) on frequent intractable dry mouth: A randomized, open-label, parallel, multi-center trial

1.2. Trial stage

- Intervention study

2. Trial institutions

2.1. Clinical trial institution

- Kyung Hee University School of Medicine, Kyung Hee University Medical Center

2.2. Co-clinical trial institutions

- Myongji Hospital / Su Young Jung
- Inha University Hospital / Ji Won Kim
- Kyung Hee University Hospital at Gangdong / Young Chan Lee

3. Principal investigator, Co-investigator, and clinical pharmacist

3.1. Principal investigator

- Department of Otolaryngology-Head and Neck Surgery, Kyung Hee University School of Medicine, Kyung Hee University Medical Center / Young-Gyu Eun

3.2. Co-investigator

- Department of Sasang Constitutional Medicine, Kyung Hee University College of Korean Medicine, Kyung Hee University Korean Medicine Hospital / Junhee Lee

3.3. Research assistants

- Department of Otolaryngology-Head and Neck Surgery, Kyung Hee University School of Medicine, Kyung Hee University Medical Center / Jung Min Park
- Department of Clinical Pharmacology and Therapeutics, Kyung Hee University Hospital / Min Jung Lee

3.4. Clinical pharmacist

Kyung Won Yoon / Pharmaceutical Headquarters, Kyung Hee University Medical Center, Pharmacist
Hye Jung Hong / Pharmaceutical Headquarters, Kyung Hee University Medical Center, Pharmacist

4. The objective and background of the study

4.1. Objective

- To ascertain whether combined administration of BIT herbal medicine and low-dose pilocarpine can demonstrate both non-inferior efficacy and fewer side effects in patients with frequent intractable xerostomia (patients who underwent radiation therapy to the head and neck or with Sjogren's syndrome) than the administration of only high-dose pilocarpine.

4.2. Backgrounds

4.2.1. Background for diseases

- Xerostomia, dry mouth, or oral dryness is defined as the subjective perception of dry mouth caused by various factors. Depending on the mechanism, the salivary gland function is

normal, but there are pseudo-dryness, in which the patient complains subjectively of dry mouth, and genuine dry mouth, caused by a decrease in salivary gland function. When the stimulated salivary flow rate is < 0.7 ml/min or the unstimulated salivary flow rate is < 0.1 ml/min, the condition is generally defined as salivary gland hypofunction.

- Intractable xerostomia is a condition in which dry mouth symptoms and salivary gland hypofunction persist even after treatment, including improvement in living conditions and stimulation of the salivary glands. The prevalence of xerostomia in patients with Sjogren's syndrome or those who have received radiotherapy for head and neck cancer is almost 100%, making them the representative causes of intractable xerostomia (Fox PC. 2007; Shiboski, et al. 2007).

4.2.2. Backgrounds for conventional treatments

- The treatment of intractable xerostomia requires addressing systemic diseases, adjusting medications and modifying lifestyle factors. Local spray medications, sugar-free gums, adequate hydration, and lubricants are commonly employed. Local spray drugs can be divided into saliva gland secretion stimulants including gum and candy, and saliva alternatives. Saliva gland secretion stimulants include stimulants including malic acid, sugar-free gum, mouth rinse, gels, and toothpaste, which stimulate saliva secretion. Saliva substitute contains olive oil, betaine, glycerol, etc., similar to natural saliva and acts by increasing the salivary viscosity.
- However, these treatments have certain limitations in patients who have received radiotherapy for head and neck cancer or with Sjogren's syndrome, which are the main causes of intractable xerostomia. Pilocarpine has been approved by the US Food and Drug Administration (FDA) to increase salivary secretion in these patients. However, its efficacy diminishes when a significant portion of the glands is damaged. It often exhibits more side effects at high-doses and limited effectiveness at low-doses. Prolonged high-dose

pilocarpine use may lead to various side effects, including sweating, chills, nausea, dizziness, increased urinary frequency, and palpitations. Moreover, the use of pilocarpine is contraindicated in patients with severe asthma, chronic obstructive pulmonary disease, glaucoma, iritis, and taking beta-blockers.

- In other words, the treatment of intractable xerostomia currently remains at an auxiliary level rather than a fundamental treatment. In addition, systemic saliva secretions are accompanied by many side effects, thus thorough confirmation and attention are required. Therefore, as a more fundamental treatment, it is necessary to develop and introduce a convergence treatment technology that helps regenerate salivary glands that can function as secretions and suppresses the inflammatory response of salivary glands.

4.3. Backgrounds on clinical trial drugs

4.3.1.1. Pilocarpine

- In the case of pilocarpine, pilogen tab 5 mg is planned to be used. Pilocarpine is a natural compound extracted from Pilocarpus jaborandi, a South American shrub. This vegetable alkaloid, an acetylcholine muscarinic M3 receptor agonist, functions as a sialagogue that stimulates the secretion of exocrine glands, including the parotid and submandibular glands. Pilocarpine has been proven to be effective in patients with xerostomia after radiotherapy in several clinical studies. In particular, compared to artificial saliva, candy, and water intake, dryness and discomfort in the oral cavity were significantly improved, and the secretion of actual saliva was also increased in case of pilocarpine administration. In addition, even in patients with Sjogren's syndrome, the administration of pilocarpine has been demonstrated in clinical studies to improve symptoms significantly for dry mouth and eye. Pilocarpine is the only drug in Korea that can be used to stimulate salivary secretion. However, the administration of pilocarpine is limited in patients because it can be accompanied by side effects such as palpitations and hyperhidrosis. In addition, there is a

disadvantage that pilocarpine should be taken frequently and continuously due to its short action time low cumulative effect.

- In this study, the control group was set to administer the common dose of pilocarpine, and the experimental group was set to administer half dose of pilocarpine together with Baekhogainsam-Tang.

4.3.1.2. Baekhogainsam-Tang

- The results of previous studies about use of Baekhogainsam-Tang for xerostomia are as follows.
  - After the administration of Baekhogainsam-Tang for xerostomia in elderly patients, 60% of 30 patients reported an improvement in symptoms (Masahiro UMINO, et al. Effect of Byakko-ka-Ninjin-to on Xerostomia in elderly patients -Analysis of the relationship between improvement of subjective symptom and Kampo diagnosis-. Kampo Medicine. 1994;45(1):107-113.)
  - As a result of administering Baekhogainsam-Tang for 12 weeks to 11 patients who complained of dry mouth and thirst due to taking disopyramid phosphate, it was reported that Baekhogainsam-Tang is effective in improving thirst, a common side effect of disopyramid phosphate (Shuji YAKUBO, et al. The Effects of Byakko-ka-ninjin-to on Patients in whom Thirst has been induced by Disopyramide Phosphate. Kampo Medicine. 1995;46(3):433-438.)
  - In one previous animal experiment, it has been reported that Baekhogainsam-Tang has a significant effect on oral dryness induced by anticholinergic drugs. (Masaru Sakaguchi, et al. Effects of Byakko-ka-ninjin-to on salivary secretion and bladder function in rats. J Ethnopharmacol. 2005 Nov 14;102(2):164-9.)

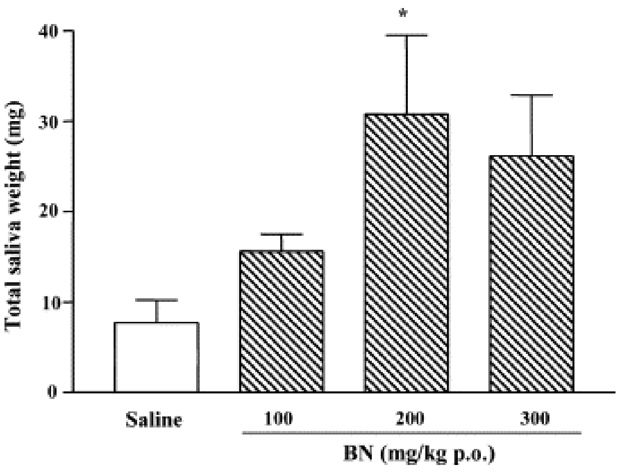

-

Figure 1. Effect of BN on salivary secretion in anesthetized rats. Byakko-ka-ninjin-to was administered p.o. Each column and vertical bar represent the mean ± S.E. of six animals. *P < 0.05 compared to control (saline 500 µL/animal, p.o.) group.

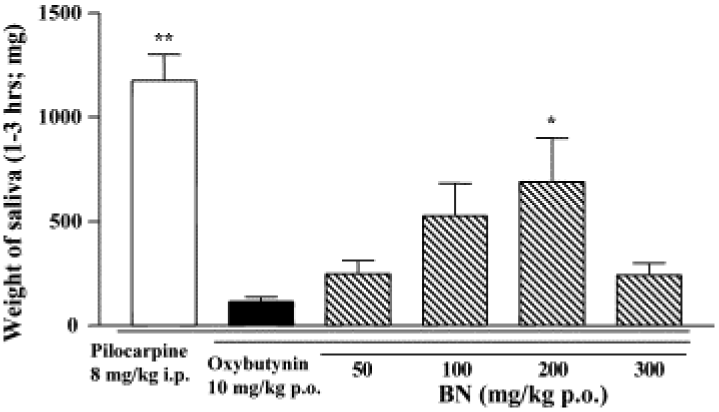

Figure 2. Effect of Byakko-ka-ninjin-to on salivary secretions (1–3 h) in anesthetized rats treated with oxybutynin and pilocarpine. BN was administered p.o. just before administration of oxybutynin. After 10 min, pilocarpine was injected i.p. Each column and vertical bar represent the mean ± S.E. of six animals. *P < 0.05 and **P < 0.01 compared to oxybutynin (10 mg/kg, p.o.) + pilocarpine-treated group (8 mg/kg, i.p.).

- In the animal experiment evaluating the effect of administration of Baekhogainsam-Tang on the autonomic nerves acting as the sublingual salivary glands, Baekhogainsam-Tang has been reported to promote saliva secretion. (A Niijima, et al. Effect of byakko-ka-ninjin-to on the efferent activity of the autonomic nerve fibers innervating the sublingual gland of

the rat. J Auton Nerv Syst. 1997 Mar 19;63(1-2):46-50.)

- The Baekhogainsam-Tang granules will be purchased from Hanpoong Pharmaceutical & Food Co., Ltd. Baekhogainsam-Tang (BIT; Baihu Jia Renshen-tang in Chinese; Byakko-ka-ninjin-to in Japanese) is known to be effective in treating symptoms such as flushing, thirst, and sweating due to dehydration during the chronic stage of high fever. BIT, widely used in Korea, China, and Japan, has been approved by the Korea FDA for clinical use in patients complaining of sore throat and thirst. Thus, BIT is expected to have a similar effect while reducing the dose of pilocarpine by treating xerostomia caused by radiotherapy or Sjogren's syndrome. In this clinical trial, the only experimental group was set to administer BIT 4 g three times a day, referring to the permitted dosage of Hanpoong Pharmaceutical & Food Co., Ltd.
- The results of representative studies on the stability of long-term treatment of BIT for xerostomia are as follows.
  - Using Sprague-Dawley (SD) Rat as an experimental animal, a single-dose toxicity test and a 13-week repeated-dose toxicity test were conducted for BIT (TJ-34). In the single-dose toxicity test, 2g/kg and 8g/kg were administered to 10 SD rat groups (5 males and 5 females). No adverse reactions related to death or medication were observed, and there were no abnormal findings in subsequent autopsy. The lethal dose is estimated to be 8 g/kg.
  - In the repeated-dose toxicity test, SD rat was divided into control group (n=40, 20/sex, 0 mg/kg) and experimental group 1 (n=20, 10/sex, 125 mg/kg), experimental group 2 (n=20, 10/sex, 500 mg/kg), and experimental group 3 (n=40, 20/sex, 2000 mg/kg), and some of the control group (n=20, 10/sex, 0 mg/kg) and experimental group 3 (n=20, 10/sex, 2000 mg/kg) were additionally observed for 4 weeks after the end of administration. No adverse reactions related to death or medication were observed during the experimental process,

and body weight and water and feed intake were not affected by medication. No change was observed in urine and ophthalmic tests after the end of administration, and no change was observed in hematologic and biochemical tests. In post-experimental autopsy, no abnormal findings related to medication were observed in organ weight, visual and histologic examination. In conclusion, the nontoxic dose of BIT is estimated to be 2000 mg/kg/day in a laboratory setting. (Minematsu S., Yoshinaga K., Takei H., et al. A single oral dose toxicity study and a 13-week repeated dose study with a 4-week recovery period of TSUMURA Byakko-ka-ninjin-to (TJ-34) in rats. Japanese Pharmacology and Therapeutics 1995 23:SUPPL. 7 (169-189))

4.4. Necessity of the clinical trial

- Similar to pilocarpine, BIT would effectively relieve symptoms in patients with intractable xerostomia. We hypothesized that patients with intractable xerostomia taking both BIT and low-dose pilocarpine would have similar symptom relief but fewer side effects compared with those taking only high-dose pilocarpine. Thus, we aimed to ascertain whether combined administration of BIT herbal medicine and low-dose pilocarpine can demonstrate both non-inferior efficacy and fewer side effects in patients with frequent intractable xerostomia (patients who underwent radiation therapy to the head and neck or with Sjogren’s syndrome) than the administration of only high-dose pilocarpine.

4.5. Evidence for co-administration test design

- Pilocarpine, an acetylcholine muscarinic M3 receptor agonist, functions as a sialagogue that stimulates the secretion of exocrine gland.
- However, since the half-life of pilocarpine is short within 3 hours, and there is no cumulative effect, it should be taken several times per day (2-4 times). Meanwhile, the side effects of pilocarpine are characterized by excessive parasympathetic nerve-like effects. These include headache, visual field disorder, tears, sweating, dyspnea, gastrointestinal dystrophy, nausea,

vomiting, diarrhea, atrioventricular block, tachycardia, bradycardia, hypotension, hypertension, shock, delirium, arrhythmia, and progression. However, there are no other drugs available to patients with xerostomia in actual clinical practice due to pilocarpine' side effects.

- In this study, we want to confirm the effect of administrating both pilocarpine at half dose of commercial dose (previous clinical trial reported that half dose of pilocarpine was significantly less effective than the commercial dose) and Baekhogainsam-Tang (increasing salivary gland secretion, and improving the salivary gland secretion of pilocarpine) compared to administrating only pilocarpine at commercial dose. In other words, the design basis and goal of this study is to verify that the co-administration of Baekhogainsam-Tang has the same efficacy and reduced side effects while reducing the administration of pilocarpine.
- The paper on the basis of co-administration

  Pilocarpine, a muscarinic agonist, significantly increases salivary gland secretion, but its action is inhibited by anticholinergic drugs such as oxybutynin and propionate. In animal experiments, the salivary glands' stimulation of pilocarpine inhibited by oxybutynin was significantly recovered when BIT was co-administered.

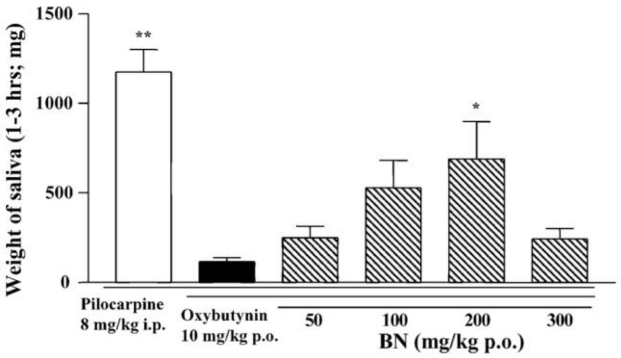

Fig. 4. Effect of BN on salivary secretions (1–3 h) in anesthetized rats treated with oxybutynin and pilocarpine. BN was administered p.o. just before administration of oxybutynin. After 10 min, pilocarpine was injected i.p. Each column and vertical bar represents the mean ± S.E. of six animals. *$P<0.05$ and **$P<0.01$ compared to oxybutynin (10 mg/kg, p.o.) + pilocarpine-treated group (8 mg/kg, i.p.).

(Masaru S., Kazuhiro G., Hiroyuki I.,

et al. Effects of Byakko-ka-ninjin-to on salivary secretion and bladder function in rats. J Ethnopharmacol. 2005 Nov 14;102(2):164-9.)

5. The clinical trial drug information

5.1. Overview of clinical trial drugs

5.1.1. Pilocarpine

Product name	Pilogen Tab.
Manufacturer	CTC Bio, Inc.<br>(Seoul, Republic of Korea)
Approval date	2008-Jun-18
Formulation	white circular film coating tablet
Components / Amounts	Pilocarpine Hydrochloride 5 mg
Authorized efficacy effect	The treatment of symptoms of dry mouth from salivary gland dysfunction caused by radiotherapy for head and neck cancer.<br>The treatment of symptoms of dry mouth or dry eye in patients with Sjogren's syndrome.
Dosage & Administration	Experimental group: half table (2.5 mg) four times a day, taken orally for 12 weeks<br>Control group: one tablet (5 mg) four times a day, taken orally for 12 weeks<br>(four times: after each meal and before bedtime)
Storage	Airtight container, room temperature (1-30℃)

5.1.2. Baekhogainsam-Tang

Product name	Baekhogainsam-tang Ext. Granule
Manufacturer	Hanpoong Pharmaceutical & Food Co., Ltd.<br>(Jeonju, Republic of Korea)
Approval date	1979-Sep-18

<table>
<tr><td>Formulation</td><td>grayish-brown granule</td></tr>
<tr><td>Components / Amounts</td><td>Single dose (4 g / dose)
<table>
<tr><th>Application category</th><th>Scientific name</th><th>standard</th><th>Amount (mg)</th><th>Active substance capacity</th></tr>
<tr><td>Main component</td><td>Anemarrhena Rhizome</td><td>KP</td><td>2,000</td><td></td></tr>
<tr><td>Main component</td><td>Gypsum</td><td>KHP</td><td>5,330</td><td></td></tr>
<tr><td>Main component</td><td>Licorice</td><td>KP</td><td>660</td><td>13.2 mg as glycyrrhizinate</td></tr>
<tr><td>Main component</td><td>Oryzae Semen</td><td>KHP</td><td>3,300</td><td></td></tr>
<tr><td>Main component</td><td>Ginseng</td><td>KP</td><td>1,000</td><td>2.0 mg as ginsenoside Rb1</td></tr>
<tr><td></td><td>Soft extract</td><td></td><td>830</td><td></td></tr>
<tr><td>Diluent</td><td>Lactose Hydrate</td><td>KP</td><td>2,260</td><td></td></tr>
<tr><td>Excipients</td><td>Corn Starch</td><td>KP</td><td>Quantum sufficit</td><td></td></tr>
<tr><td>Excipients</td><td>Hydroxypropyl Cellulose</td><td>KP</td><td>Quantum sufficit</td><td></td></tr>
<tr><td>Excipients</td><td>Sucrose Fatty Acid Ester</td><td>KP</td><td>Quantum sufficit</td><td></td></tr>
<tr><td>Excipients</td><td>Magnesium Stearate</td><td>KP</td><td>Quantum sufficit</td><td></td></tr>
<tr><td>Excipients</td><td>Light Anhydrous Silicate</td><td>KP</td><td>Quantum sufficit</td><td></td></tr>
<tr><td></td><td>Total</td><td></td><td>4,000</td><td>100%</td></tr>
</table></td></tr>
<tr><td>Authorized efficacy effect</td><td>The treatment of symptoms such as flushing, thirst, and sweating</td></tr>
<tr><td>Dosage & Administration</td><td>Experimental group: One packet (4 g) three times a day,
taken orally for 12 weeks
(three times: before each meal)
Control group: None</td></tr>
<tr><td>Storage</td><td>Airtight container, room temperature (1-30℃)</td></tr>
</table>

5.2. Packing and labeling of clinical trial drugs

- Among the clinical trial drugs in this study, pilogen tabs are manufactured by CTC Bio, Inc., and Baekhogainsam-tang Ext. Granules are manufactured by Hanpoong Pharmaceutical & Food Co., Ltd., and delivered to the Clinical pharmacy of Kyung Hee University Medical Center. The drugs shall be labeled as follow in accordance with Article 69-6 of the Regulations on the Safety of Drugs, etc (Amended on Mar. 23, 2013). The clinical pharmacy of Kyung Hee University Medical Center labels drugs and delivers them to Myongji Hospital, Inha University Hospital, Kyung Hee University Hospital at Gangdong's clinical pharmacy.
- Depending on the visit schedule of the participants, clinical trial drugs are packed in airtight container for 30 days from 'Visit2' to 'Visit 4'.

1. Mark "for clinical trials"<br>2. Code name of the product or general name of the principal component<br>3. Manufacturing number and valid deadline or re-inspection date<br>4. Storage methods<br>5. Trade name and address of the person who has received approval for the clinical trial plan<br>6. Indication "Not available for use for purposes other than clinical trials"

5.3. Methods of management and storage of clinical trial drugs

- The clinical trial pharmacist is responsible for the acquisition, storage, preparation, management, and return of drugs used in clinical trials. The clinical trial pharmacist shall confirm in writing the receipts and quantity of clinical trial drugs received, sign them, and manage them appropriately.
- Clinical trial drugs are administered by the clinical trial pharmacist according to the principal investigator's prescription, and the clinical trial pharmacist must accurately record the

clinical trial drugs and management given to each participant.

- This clinical trial is an open clinical trial, identified by the test subject identification number, and the clinical trial drug prescribed to each test subject and the returned clinical trial drug should be properly recorded.
- When the clinical trial is suspended or terminated, the clinical trial pharmacist must return the unused clinical trial drug to the clinical pharmacy of Kyung Hee University Medical Center and store the return certificate, and the clinical pharmacy of Kyung Hee University Medical Center must discard the returned clinical trial drug.

6. The duration of the study

- IRB approval date ~ 4 years

7. Test subjects

- Patients with frequent intractable xerostomia (patients who underwent radiation therapy to the head and neck or with Sjogren's syndrome)

8. Inclusion criteria, exclusion criteria, sample size, and basis for calculation

8.1. Inclusion criteria

- One or more of the following
  - Patients who undergone radiation therapy to the head and neck
  - Patients with Sjogren's syndrome with both followings

    (1) positive anti-Ro/SS-A antibody reaction

    (2) Saliva flow rate (Unstimulated) ≤ 0.1mL/min

- unstimulated SFR ≤ 0.25 ml/min
- VAS score for dry mouth over the past one month ≥ 4
- age >19 years
- compliance with all written informed consent

8.2. Exclusion criteria

- Patients with the following past medical history
  - heart failure
  - medication-refractory hypertension (systolic blood pressure ≥ 160 mmHg or diastolic blood pressure ≥ 100 mmHg)
  - bronchial asthma
  - arrhythmia accompanied by clinical symptoms (fainting, palpitation, or shortness of breath) or requiring treatments (sinus failure, atrial conduction block, atrial fibrillation, atrial junction dependent tachycardia, ventricular tachycardia)
  - coronary artery disease
  - asthma, chronic obstructive pulmonary diseases, chronic bronchitis
  - inborn errors of amino acid metabolism
  - hepatic encephalopathy
  - ophthalmic diseases (such as narrow angle glaucoma, peripheral retinopathy, and iritis)
- Clinical laboratory test results falling within the following ranges (within 7 days prior to the start of clinical trial drug administration)
  - hemoglobin < 8.0 g/dL
  - aspartate transaminase (AST) or alanine transaminase (ALT) > 3 times the upper limit of each hospital standard
  - total bilirubin > 2 times the upper limit of each hospital standard

- creatinine >1.5 times the upper limit of each hospital standard, or creatinine clearance ≤ 60 mL/mi

- Patients taking following medications are also excluded: coumadin, heparin, warfarin, aspirin, anticholinergics, first-generation antihistamines, antidepressants with anticholinergic effects, monoamine oxidase inhibitors, diuretics, mineralocorticoids, β-adrenaline antagonist, or digitalis; patients taking low-dose aspirin or β-adrenaline antagonist can participate in the protocol based on the cardiologist's opinion.
- pregnancy or lactation, disagreement with proper contraception
- Failure to use appropriate contraception methods (male participants), participants who did not agree to contraception for 4 weeks after the end of administration of the trial drug (both male and female)
- galactose intolerance, Lapp lactase deficiency, or glucose-galactose malabsorption
- difficulty in oral administration of a clinical trial drug
- significant hypersensitivity to Baekhogainsam-Tang or components
- significant hypersensitivity to pilocarpine
- Participants who is deemed unsuitable for participation in clinical trials at the discretion of the investigator due to various reasons including screening test results
- cognitive disorder; inability to read and write

8.3. Sample size

- 120 participants (Experimental group 60, control group 60)
- Sample size by clinical trial institution

	Kyung Hee University Medical Center	Inha University College of Medicine	Myongji Hospital	Kyung Hee University Hospital at Gangdong	Total

Experimental group	32	16	2	10	60
Control group	32	16	2	10	60
Total	64	32	4	20	120

- Competitive recruitment
- Sample size in Kyung Hee University Medical Center: 64 (experimental 32, control 32)

8.4. Basis for sample size calculation

- The aim of this prospective, randomized, open-label, parallel-group, multi-center, and controlled trial is to ascertain whether combined administration of BIT herbal medicine and low-dose pilocarpine can demonstrate both non-inferior efficacy and fewer side effects in patients with frequent intractable xerostomia (patients who underwent radiation therapy to the head and neck or with Sjogren's syndrome) than the administration of only high-dose pilocarpine.
- The assumptions for calculating the valid sample size are as follows.

① One-sided Z test (Non-inferiority test)

② Level of significance (α) = 0.05 (Considering this is an intervention study)

③ Type 2 error (β) = 0.1 (Power of the test = 90%)

④ Experimental group: Control group = 1 : 1 (λ=1)

⑤ Primary outcome: unstimulated salivary flow rate (SFR) after 12 weeks of treatment

- σ = 0.35 / d (non-inferiority limit) = 0.21

In the previous study, the mean ± standard deviation (SD) of unstimulated SFR for 5 mg pilocarpine therapy vs placebo group after 12 weeks of medication, respectively, were 0.38 ± 0.48 vs 0.17 ± 0.13 mL/min (p <0.001). Based on these results, the SD was calculated to be 0.35.

In consideration of the exploratory aspects of the clinical trial, the point estimation (μcIH -

μpIH; 0.38−0.17=0.21) after 12 weeks of administration of 5 mg pilocarpine and placebo was set as the non-inferiority limit.

- Reference: Vivino FB, et al. Pilocarpine tablets for the treatment of dry mouth and dry eye symptoms in patients with Sjögren syndrome: a randomized, placebo-controlled, fixed-dose, multicenter trial. P92-01 Study Group. Arch Intern Med. 1999. PMID: 9927101 Clinical Trial.
- The H01 and H11 of this clinical trial are as follows.

H01: μc - μt > 0.21

H11: μc - μt ≤ 0.21

μc = SFR after 12 weeks of administration of only pilocarpine one tablet

μt = SFR after 12 weeks of combined administration of Baekhogainsam-tang herbal medicine and low-dose pilocarpine

$$n_c = \frac{(1+\lambda)\sigma^2 (Z_\alpha + Z_\beta)^2}{\lambda(\,|\,\mu_c - \mu_t\,|\, - d)^2}$$

Nc=Nt≒48

- Assuming a 20% dropout rate, we calculated the sample size as 120 participants (60 in each group).

	Control	Experimental	Total
Resultant sample size	48	48	96
Sample size considering 20% dropout	60	60	120

9. Trial design

9.1. Trial design

- This study is a prospective, randomized, open-label, parallel-group, multi-center, and controlled trial involving participants from four clinical research centers in Korea. Eligible participants will be randomly allocated to either the experimental or control group after screening based on the inclusion and exclusion criteria. The patients will receive treatment for 12 weeks. The unstimulated and stimulated salivary flow rate (SFR) test, visual analog scale (VAS), salivary scintigraphy, and questionnaires for oral symptoms and quality of life will be assessed for up to 12 weeks after the first visit.

9.2. Trial schedule

Timepoint / Assessment	Screening Period	Treatment period						
	-1w - 0w	1w	2-3w	4w	5-7w	8w	9-11w	12w
	V1 (-1w~0d)	V2 (0w)		V3 (4w±2d)		V4 (8w±2d)		V5 (12w±2d)
Informed consent	●							
Demographics	●							
Medical examination	●							
Physical examination	●	●		●		●		●
Vital signs	●	●		●		●		●
Blood tests	●			●		●		●
12 lead ECG	●							
Random allocation	●							
Pilocarpine[1)]		●	●	●	●	●	●	●
Baekhogainsam-Tang[2)]		●	●	●	●	●	●	●
OHIP-14		●		●		●		●
EQ-5D-5L		●		●		●		●
VAS	●			●		●		●
Salivary flow test (SFR)	●			●		●		●
Salivary scintigraphy		●						●
Monitoring adverse events		●		●		●		●
Evaluating concomitant medication		●		●		●		●

9.3. Dosage and administration of the trial drugs

		Dosage	Administration	periods
Experimental group	Pilogen tab	half table (2.5 mg) four times a day	Four times a day (after each meal and before bedtime)	12 weeks
	Baekhogainsam-tang Ext. Granule	One packet (4 g) three times a day	Three times a day (before each meal)	12 weeks
Control group	Pilogen tab	one tablet (5 mg) four times a day	Four times a day (after each meal and before bedtime)	12 weeks

9.4. Combination therapy

- None

9.5. Random allocation

9.6. Methods of registration and randomization

- An independent statistician, blinded to the study design and purpose, will perform the random allocation of a total of 120 patients to the experimental group or the control group in a 1:1 ratio using a stratified block randomization method (a block size of 4).
- Random numbers will be generated by a computerized random-number generator using the block-randomization method of SAS version 9.1.3 (SAS Institute Inc., NC, Cary, USA) for sequence generation.

9.7. Managements of random allocation

- When a random assignment table is prepared, it is managed by an independent statistician.
- When the test subjects listen to the explanation of the study and write a consent form for participation in the clinical trial, they are given screening numbers in order.
- Participants who pass the screening test during their first visit will be assigned a registration number and group based on the random assignment table stored by an independent statistician.

9.8. Test subject number

- A screening number is assigned to the test subjects who have agreed to participate in the clinical trial in the order of time of written informed consent. The screening number starts with KH-S001, and the rest, excluding KH-, consists of four digits in total and three numbers (Kyung Hee University Medical Center 'KH-', Inha University Hospital 'IH-', Myongji Hospital 'MJ-', and Kyung Hee University Hospital at Gangdong 'KD-'). Finally, test subject number (registration number) is assigned in order of the data the screening passes were determined (in the order of the time of informed consent if the same date) and assigned to the group according to a pre-written random assignment table. The test subject identification number starts with each KH-R1001, and the rest, excluding KH-, consists of five digits and four numbers (Kyung Hee University Medical Center 'KH-', Inha University Hospital 'IH-', Myongji Hospital 'MJ-', and Kyung Hee University Hospital at Gangdong 'KD-'), The identification number shall be assigned as follows. One (or two) of the first number is assigned to the group, and the last three numbers are assigned in the order of random assignments. The screening number and test subject identification number assigned to each test subject are used as codes to recognize the test subject until the end of the clinical trial.

9.9. Clinical trial schedule

9.9.1. Screening visit

- Screening tests for the test subjects who have agreed in writing to participate in the clinical trial to review the suitability are performed <u>on an empty stomach for at least 8 hours</u> within 7 days (-7d) from the first intervention date (1 week), excluding those with clinically significant abnormalities by the following tests. For reference, if the abnormal findings in the screening test item are judged to be a temporary change, the suitability may be

reexamined through a re-examination before the first intervention date.

- Demographics and medical examination
- Vital signs and physical examination
- Clinical laboratory examination (hematologic test / biochemical test / urinalysis / immunology test / blood clotting test / serological examination)
- 12-lead ECG
- Salivary flow rate (SFR) test
- Evaluation of visual analog scale (VAS) for xerostomia

9.9.2. Visit 2~5 (Week 0~12)

- Eligible participants are randomly allocated to either the experimental or control group after screening based on the inclusion and exclusion criteria and written informed consent.
- Both experimental and control groups receive 4-week clinical trial drugs every 4 weeks (Before administration (0 weeks, Visit 2), 4 weeks after administration (4 weeks ± 2 days, Visit 3), and 8 weeks after administration (8 weeks ± 2 days, Visit 4)).
- The following tests will be conducted at each visit, including before administration (0 weeks, Visit 2), 4 weeks after administration (4 weeks ± 2 days, Visit 3), 8 weeks after administration (8 weeks ± 2 days, Visit 4), and 12 weeks after administration (12 weeks ± 2 days, Visit 5).
  - Assessment of drug compliance and return of unused clinical trial drugs
  - Monitoring adverse events and evaluating concomitant medication
  - Vital signs and physical examination
  - Clinical laboratory examination (hematologic test / biochemical test / urinalysis)
  - Salivary flow rate (SFR) test
  - Evaluation of visual analog scale (VAS) for xerostomia

- Salivary scintigraphy
- 14-item Oral Health Impact Profile (OHIP-14) questionnaire
- 5-level EuroQol 5-dimensional (EQ-5D-5L) questionnaire

9.10. First aid medicine

- No additional first aid medication

9.11. Prohibited drug in combination

Coumadin, heparin, warfarin, aspirin, anticholinergics, first-generation antihistamines, antidepressants with anticholinergic effects, monoamine oxidase inhibitors, diuretics, mineralocorticoids, β-adrenaline antagonist, or digitalis. Patients taking low-dose aspirin or β-adrenaline antagonist can participate in the protocol based on the cardiologist's opinion.

9.12. Permitted drug in combination

- Drugs other than prohibited drugs in combination are allowed, but they are reviewed by principal investigator and sub-investigators during screening and clinical trials period.

10. Observation items and clinical examination items

10.1. Screening evaluation items

- Evaluate and inspect the following items to select the test subjects suitable for participation in clinical trials based on the inclusion and exclusion criteria.

10.1.1. Evaluation of general items

- Gender, age
- Past medical history, recent medical history, and drug use history
- Information on smoking and alcohol intake

- History of head and neck surgery and radiotherapy

10.1.2. Vital signs, physical examination

- Blood pressure, pulse rate (measured with no sudden change in position for more than 3 minutes), and respiratory rate
- Body temperature (tympanic)
- Height (cm), Weight (kg)

10.1.3. 12-lead ECG

- Record the ventricular rate (beats/min), PR interval (msec), QRS (msec), QT/QTc (msec) from the automatic analysis & recording.

10.1.4. Clinical laboratory examination

10.1.4.1. Hematology test

- WBC with differential count (segmented neutrophil, lymphocyte, monocyte, eosinophil, basophil), RBC, hemoglobin, hematocrit, platelet

10.1.4.2. Biochemical test

- glucose, BUN, uric acid, cholesterol, total protein, albumin, total bilirubin, alkaline phosphatase, AST, ALT, γ-GTP, CPK, LDH, creatinine, sodium, potassium, chloride, triglyceride, HDL, LDL, C-reactive protein, phosphorus, calcium

10.1.4.3. Urinalysis

- pH, specific gravity, protein, bilirubin, glucose, urobilinogen, ketone, nitrite, blood, leukocyte, microscopy (Urine RBC, Urine WBC, Urine Epithelial Cell, others)

10.1.4.4. Immunology test

- ANA, Anti-Ro/SSA, Anti-La/SSB

10.1.4.5. Blood clotting test

- PT, aPTT

10.1.4.6. Serological examination

- HBsAg, anti-HBs Ab, anti-HCV Ab, IgM anti-HAV Ab

10.1.5. Salivary flow rate (SFR) test

- Measuring by collecting saliva in both unstimulated and stimulated state
- (Unstimulated) The participants will abstain from food intake for at least one hour, and they will need to rinse their mouth with water, held it open, and gather saliva for 5 min.
- (Stimulated) The participants will be encouraged to suck sugar-free lemon candy. The procedure for measuring the stimulated salivary flow is the same as that for measuring the unstimulated salivary flow.

10.1.6. Evaluation of visual analog scale (VAS) for xerostomia

- Evaluate xerostomia-related symptoms.
- The degree of symptoms is assessed using the VAS (a numerical measure of current pain with '0' no discomfort and '10' intolerable very severe discomfort).

10.2. Evaluation of efficacy

10.2.1. Salivary flow rate (SFR) test

- Visit 2 (Week 0) is replaced by screening test.
- Evaluation at Visit 3 (Week 4), Visit 4 (Week 8), Visit 5 (Week 12).

10.2.2. Evaluation of VAS for xerostomia

- Visit 2 (Week 0) is replaced by screening test.
- Evaluation at Visit 3 (Week 4), Visit 4 (Week 8), Visit 5 (Week 12).

10.2.3. Salivary scintigraphy

- Evaluation at Visit 2 (Week 0) and Visit 5 (Week 12).
- For the objective evaluation of salivary gland function, salivary scintigraphy will be performed after the administration of a radiotracer (Technium-99m pertechnetate).
- After intravenous administration of Tc 99m sodium permeate 370 MBq to patients, images are obtained using a low energy-high sensitivity collimator-type dual-head gamma camera (Philips Forte, Philips Medical Systems).
- Images are obtained 5 min, 15 min, and 30 min after intravenous administration of radiotracer, and front images are obtained in the same way after administration of salivary gland secretion stimulators (lemon candy).
- During the examination, the patient's head is fixed and instructed not to swallow saliva.
- To analyze the scintigraphy images, the counts of the regions of interest (ROI) will be evaluated in the parotid and submandibular glands. Three parameters – uptake ratio (UR), maximum accumulation (MA), and maximum secretion (MS)– will be calculated using the following formulas
- (1) UR is calculated as the salivary gland count – the background count.
- (2) The MA (%) is calculated as follows: (maximum activity before stimulation – initial activity 5 min after radiotracer injection) × 100 / maximum activity before stimulation.
- (3) The MS (%) is calculated as (maximum activity prior to stimulation – activity after stimulation) × 100 / peak activity before stimulation.

10.2.4. OHIP-14 (The 14-item Oral Health Impact Profile) Questionnaire

- Evaluation at Visit 2 (Week 0), Visit 3 (Week 4), Visit 4 (Week 8), and Visit 5 (Week 12).
- Subjective quality of life will be evaluated using OHIP-14 Korean version. The responses will be measured on a scale ranging from 0 (never) to 4 (always), generating a total score of 0 to 56 (lowest quality).

How often do the following situations occur due to a disease in your mouth?					
	0<br>Never	1<br>Rarely	2<br>Occasionally	3<br>Frequently	4<br>Always
Problems with pronunciation					
Bad sense of taste					
Pain					
Discomfort when eating					
Concern for the mouth					
Self-consciousness due to oral problems					
Dissatisfaction with food intake					
Interruption of meals					
Difficulty relaxing due to oral problems					
Embarrassment arising from oral problems					
Irritability					
Problems at work due to oral problems					
Found life less satisfying due to oral problems					
Complete inability to function					

10.3. Evaluation of safety

10.3.1. Adverse events evaluation

- The severity of AE will be assessed in accordance with the MedDRA version 23.1.

- From the start of clinical trial drug administration to the end of administration, the occurrence and grade of adverse events are evaluated at each visit.
- The basic principles of grading are as follows

Grade 1: Side effects that are symptomatic but do not require treatment<br>Grade 2: Side effects that require treatment to alleviate symptoms but do not interfere with activities of daily living (ADL) and do not pose a threat to life<br>Grade 3: Side effects that can be life-threatening if left untreated<br>Grade 4: Life-threatening side effects

10.3.2. Vital signs

- Measuring at every visit.
- Blood pressure, pulse rate, respiratory rate, and body temperature

10.3.3. Clinical laboratory examination

- Evaluation following items at Visit 1 (Screening), Visit 3 (Week 4), Visit 4 (Week 8), and Visit 5 (Week 12).

10.3.3.1. Hematology test

- Hemoglobin, Hematocrit, RBC, WBC, WBC differential cell count, Platelet

10.3.3.2. Biochemical test

- Alkaline phosphatase, BUN, Creatinine, γ-GTP, SGPT(ALT), SGOT(AST), Albumin, Protein, Total bilirubin, CRP, LDH, sodium, potassium, chloride, phosphorus, calcium

10.3.3.3. Urinanalysis

- pH, specific gravity, Protein, Glucose, Ketones, Bilirubin, Blood, Urobilinogen, Nitrite, WBC

10.3.3.4. Blood clotting test

- PT, aPTT

10.4. Economic assessment

- Evaluation at Visit 2 (Week 0), Visit 3 (Week 4), Visit 4 (Week 8), and Visit 5 (Week 12).
- The patients' health status will be evaluated using the EQ-5D-5L questionnaire.
- This questionnaire consists of five dimensions (mobility, self-care, usual activities, pain/discomfort, and anxiety/depression), each with five levels (no problems, slight problems, moderate problems, severe problems, and extreme problems; scores of 1 to 5). The EQ-5D-5L health states are defined by combining one level from each of the five dimensions, for a total of 3125 possible health states. (from www.euroqol.org)
- It is used as a measure of quality of life for future economic evaluation.

Under each heading, please tick the ONE box that best describes your health TODAY.

MOBILITY
I have no problems in walking about ❑
I have slight problems in walking about ❑
I have moderate problems in walking about ❑
I have severe problems in walking about ❑
I am unable to walk about ❑

SELF-CARE
I have no problems washing or dressing myself ❑
I have slight problems washing or dressing myself ❑
I have moderate problems washing or dressing myself ❑
I have severe problems washing or dressing myself ❑
I am unable to wash or dress myself ❑

USUAL ACTIVITIES *(e.g. work, study, housework, family or leisure activities)*
I have no problems doing my usual activities ❑
I have slight problems doing my usual activities ❑
I have moderate problems doing my usual activities ❑
I have severe problems doing my usual activities ❑
I am unable to do my usual activities ❑

PAIN / DISCOMFORT
I have no pain or discomfort ❑
I have slight pain or discomfort ❑
I have moderate pain or discomfort ❑
I have severe pain or discomfort ❑
I have extreme pain or discomfort ❑

ANXIETY / DEPRESSION
I am not anxious or depressed ❑
I am slightly anxious or depressed ❑
I am moderately anxious or depressed ❑
I am severely anxious or depressed ❑
I am extremely anxious or depressed ❑

Sample

10.5. Medication compliance assessment

- Adherence will be ascertained at each visit by history taking and counting the returned unused tablets and empty packaging.
- Medication compliance (%) will be evaluated by dividing the actually consumed tablets by the total numbers of tablets prescribed.

11. Predictive side effects and precautions

11.1. Pilogen

11.1.1. Predictive side effects

(1) Patients with head and neck cancer

- ① Sometimes the following adverse reactions may occur
  - Sweating, nausea, rhinitis, diarrhea, coldness, flushing, frequent urination, dizziness, helplessness, headache, indigestion, swelling, abdominal pain, amblyopia, vomiting, pharyngitis, hypertension, visual abnormalities, conjunctivitis, dysphagia, ulcer, muscle pain, itchy sense, rash, sinusitis, tachycardia, palpitations, and voice changes
- ② In rare cases, the following adverse reactions may occur.
  - Whole body: Body odor, hypothermia, mucosal abnormalities
  - Cardiovascular system: bradycardia, ECG abnormality, cardiac hypertension, fainting
  - Digestive system: loss of appetite, increased appetite, esophagitis, gastrointestinal disturbance, tongue disease
  - Hematopoietic system: leukopenia, lymph node disease
  - Nervous system: anxiety, confusion, depression, hypermotility, tardiness, nervousness, sensory abnormalities, speech disorders
  - Respiratory system: sputum increase, wheezing, yawning
  - Skin: seborrheic
  - Special sensations: hearing loss, eye pain, glaucoma

- Urogenital system: Urology disorder, uterine bleeding, urinary tract disorder

- ③ In long-term treatment, two patients with underlying cardiovascular disease experienced myocardial infarction and fainting, respectively. The causal relationship with the drug is unclear.

(2) Patients with Sjogren's syndrome

- ① Among the adverse reactions associated with this drug in two placebo-controlled trials, the most frequent were sweating, frequent urination, coldness, and vasodilation (redness), and the most frequent reason why patients discontinued treatment was sweating.
- Sometimes the following adverse reactions may occur
  - sweating, frequent urination, nausea, flushing, rhinitis, diarrhea, chills, increased saliva secretion, helplessness, headache, epidemic cold, indigestion, dizziness, pain, sinusitis, abdominal pain, pharyngitis, rash, infection, accidental trauma, allergic reaction, stomachache, visual field shaking, constipation, cough increase, swelling, facial edema, fever, fart, tongue inflammation, muscle pain, heart palpitations, itching sense, drowsiness, gastritis, tachycardia, tinnitus, incontinence, urinary tract infection, vaginitis, and vomiting
- ② In rare cases, the following adverse reactions may occur.
  - Whole body: chest pain, cyst, death, candidiasis, neck pain, neck stiffness, photosensitivity reaction
  - Cardiovascular system: angina, arrhythmia, ECG abnormalities, low blood pressure, high blood pressure, intracranial bleeding, migraine, myocardial infarction
  - Digestive system: loss of appetite, bilirubinemia, cholelithiasis, colitis, dry mouth, trim, gastritis, gastroenteritis, gastrointestinal abnormalities, gingivitis, hepatitis, liver function test abnormalities, hemoptysis, nausea and vomiting, pancreatitis, dysplasia, salivary gland hyperplasia, sputum increase, loss of taste, tongue abnormalities, tooth abnormalities

- Hematopoietic system: hematuria, lymph node disorders, platelet abnormalities, thrombocytopenia, thrombosis, leukocyte abnormalities
- Metabolism and nutrition: Peripheral edema, hypoglycemia
- Skeletal muscle system: joint pain, arthritis, bone abnormalities, natural fractures, pathological fractures, tendon disease, tooth disease
- Nervous system: aphasia, confusion, depression, abnormal dreams, emotional anxiety, hypermotility, tardiness, insomnia, leg cramps, nervousness, dysphoria, abnormal thinking
- Respiratory system: bronchitis, shortness of breath, hiccups, laryngeal spasms, laryngeal inflammation, pneumonia, viral infection
- Skin: alopecia, contact dermatitis, dry skin, eczema, nodular erythema, deprivation dermatitis, herpes simplex, skin ulcers, alveolar rash
- Special senses: cataract, conjunctivitis, dry eye, ear disease, otalgia, ocular disease, eye bleeding, glaucoma, urticaria, retinal disease, taste loss, visual abnormality
- Urogenital system: Breast pain, dysuria, mastitis, excessive menstruation, uterine bleeding, ovarian disease, pyuria, urethral pain, frequent urination, vaginal bleeding, candidiasis

11.1.2. Precautions

11.1.2.1. Warnings

- 1) Patients with severe cardiovascular disease: We could not correct the abnormalities of blood flow or beat caused by this drug. When administering this drug to patients with cardiovascular disease, it should be carefully administered under the strict supervision of the doctor.
- 2) Eye: In patients who applied this drug to the eye, especially at night, patients who had vision correction occurred impaired vision, and it has been reported to cause serious perception disorders. Care should be taken when driving at night or doing something

dangerous in a dark place.

- 3) It has been reported that this drug increases airway resistance, tension in the smooth muscle of the bronchi, and bronchial secretion. When administering this drug to patients with asthma, chronic bronchitis, or chronic obstructive pulmonary disease, it should be administered carefully under the strict supervision of a doctor.

11.1.2.2. Patients contraindicated to administration

- 1) Asthma patients
- 2) Patients with pilocarpine hypersensitivity
- 3) Ophthalmic diseases (such as narrow angle glaucoma, peripheral retinopathy, and iritis)

11.1.2.3. General attention

- ① Care should be taken when administering this drug to patients with cholelithiasis or suspected cholelithiasis, or patients with biliary tract disease. Atrophy of the gallbladder or bile duct smooth muscle can promote complications including cholecystitis, cholangitis, and biliary obstruction.
- ② This drug can increase the tension of the ureter's smooth muscle, and theoretically, it can promote renal colic (or ureter's reflux), especially in patients with kidney or ureter stones.
- ③ Choline efficacy drugs have a central nervous system effect in terms of dose. This should be considered in the treatment of patients with psychiatric disorders.
- ④ Patients should be informed that administration of this drug can cause visual impairment, especially at night, to the extent that it is impossible to drive safely.
- ⑤ Dehydration can occur if excessive sweating occurs in the patient when sufficient water cannot be consumed while taking this drug.

- ⑥ The drug's toxicity is characterized by an excess of parasympathetic nervous system-like effects. These include headache, visual field disorder, tears, sweating, dyspnea, gastrointestinal dystrophy, nausea, vomiting, diarrhea, atrioventricular block, tachycardia, bradycardia, hypotension, hypertension, shock, delirium, arrhythmia, and progression.
- Cardiovascular pharmacological actions associated with the dose of this drug include hypotension, hypertension, bradycardia, and tachycardia.
- ⑦ Based on the decreased blood clearance rate in patients with moderate liver disorder, the initial dose in these patients is 5 mg twice a day, and adjusted according to the treatment response and tolerability.
- Patients with mild liver disorder (Child-Pugh score 5-6) do not need dose reduction. No pharmacokinetic studies were conducted in patients with severe liver disorder (Child-Pugh score 10-15). The use of this drug is not recommended in these patients.

11.1.2.4. Drug interaction

- This drug is carefully administered to patients who are receiving β-adrenaline antagonists due to the possibility of cardiac conduction disorders.
- If the drug having parasympathetic nerve excitement is administered at the same time as this drug, an agonizing effect is expected. The simultaneous use of this drug and anticholinergic drugs (atropine, inhalation ipratropinium) antagonizes the effect.
- Although no typical drug interaction experiment was performed, the following drugs were co-administered to at least 10% of patients with Sjogren’s syndrome.
- Acetylsalicylic acid, artificial tears, calcium, coupled estrogen, hydroxychloroquine sulfate, ibuprofen, levothyroxine sodium salt, acetic levothyroxine, Medroxyprogesterone acetate, methotrexate, naphroxene, omeprazole, paracetamol, prednisone

11.1.2.5. Administration to pregnant women

- When 90 mg/kg per day (26 times the maximum amount per adult) was orally administered in pregnant female rats, it was associated with a decrease in average gestational body weight and an increase in skeletal changes. When 36 mg/kg per day (10 times the maximum amount per adult) was orally administered during pregnancy and lactation, the mortality rate increased, and administration of dosages more than 18 mg/kg per day (5 times the maximum amount per adult) was associated with a decrease in the survival and the average body weight of newborn babies. There is no proper and well-planned clinical trial on pregnant women, thus when administered during pregnancy, it should be used only when the benefits exceed the risk to the fetus

11.1.2.6. Administration to lactating women

- It is not known whether this drug is secreted from breast milk. However, since many drugs are transferred and secreted to breast milk, and have the potential to cause a series of adverse reactions to infants during lactation, it is necessary to stop breastfeeding or consider discontinuation of medication in consideration of the benefits of this drug to the mother.

11.1.2.7. Administration to children

- Safety and efficacy in children have not been demonstrated.

11.1.2.8. Administration for the elderly

- 1) Patients with head and neck cancer: In a trial of 15 elderly volunteers, five women exhibited a higher maximum blood concentration and lower area under the curve (AUC) compared to 10 men

- 2) When administered to elderly patients with Sjogren's syndrome, there has been a report of an increase in frequent urination, diarrhea, and dizziness.

11.1.3. Treatment when overdosing

- This drug is considered to have a fatal potential when administered more than 100mg. Treatment for overdose should be administered subcutaneously or intravenously with atropine (0.5mg to 1.0mg), and the maintenance of breathing and circulation effective for maintaining the patient's physical strength should be adjusted. In addition, when there is severe cardiovascular function decline or bronchoconstriction, it is desirable to subcutaneously or intravenously administer epinephrine (0.3mg to 1.0mg). It is not known whether this drug is dialyzable.

11.1.4. Others

- 1) In the case of oral administration of this drug to patients with renal and liver diseases, the pharmacology for this is unknown.
- 2) Carcinogenesis: Oral carcinogenesis studies at survival state were conducted in CD-1 mice and SD rats. The drug did not cause cancer in mice at any dose trial (dose administered approximately 50 times more than the maximum systemic dose clinically observed, up to 30 mg/kg/day). In rats, there was a statistically significant increase in the expression of benign pheochromocytoma in males and females and in the expression of hepatocellular adenoma in female rats at a dose of approximately 100 times more than the maximum systemic dose observed clinically. The carcinogenesis observed in rats was seen only in several batches of the maximum clinical allowance and does not appear to be associated with clinical use.
- 3) Mutagenesis: No evidence has been obtained from the following studies that the drug

can cause genotoxicity.

- ① Assessment of Bacteria for Reverse gene mutation (Salmonella and E. coli)
- ② Evaluation of in vitro chromosomal mutation in Chinese hamster ovarian cell line
- ③ Evaluate of in vivo chromosome mutation in mice (micronucleus)
- ④ Evaluation of primary DNA damage in rat hepatocyte culture (non-scheduled DNA synthesis)
- 4) Impaired fertility: The drug was orally administered to male and female rats at an 18 mg/kg/day dose (a dose approximately 100 times more than the maximum systemic dose observed clinically) indicating impaired fertility, including decreased fertility, decreased sperm motility, and morphological evidence of abnormal spermatozoa. It is unclear whether the decrease in fertility is an effect on male or female animals, or both. When dogs were given the main agent at a 3 mg/kg/day dose for 6 months (about three times the maximum adult dose), the results of impaired sperm bioactivity were shown. This result indicates that the drug may damage the fertility of men and women.
- People planning to become pregnant should be administered only when the benefits of this drug exceed fertility impairment

11.1.5. Storage and handling precautions

- 1) Keep it out of reach of children.
- 2) Be careful because replacing it in another container can cause an accident or it is not desirable in terms of quality maintenance.

11.2. Baekhogainsam-Tang

11.2.1. Predictive side effects

- (1) Skin: rash, urticaria, etc

- (2) Digestive system: loss of appetite, stomach irritation, watery stool, diarrhea, etc
- (3) Pseudo-aldosteronism: Decreased urine volume, swollen face, hands and feet, heavy eyelids, stiffened hands, high blood pressure, headache, etc. (If a medicine with a maximum dose of 1g or more as licorice is taken for a long period of time, it may cause pseudo-aldosteronism such as hypokalemia, elevated blood pressure, retention of sodium fluid, swelling, and weight gain, thus observe (measure the serum potassium level) sufficiently and stop taking it if abnormalities are found.)
- (4) Myopathy: Since myopathy may appear as a result of hypokalemia, make sufficient observation and stop taking it if abnormalities such as weakness, quadriceps cramps, paralysis, etc. are identified.

11.2.2. Precautions

- (1) The following persons shall consult with their doctors, oriental doctors, dentists, pharmacists and oriental pharmacists before taking this medicine.
- 1) HTN patients
- 2) Patients with cardiac arrest or renal impairment
- 3) Patients with swelling
- 4) Patients with weak stomachs and coldness (deprived appetite, stomach discomfort, watery stools, diarrhea, etc. can occur)
- 5) Weak patients with / without weakened physical strength (adverse reactions can easily occur and symptoms can worsen)
- 6) Pregnant woman or likely pregnant woman
- 7) Patients being treated by a doctor (who are being given other drugs)
- 8) Elderly people (In general, elderly people have a decreased physiological function, thus be careful, such as reducing the dose of the drug)

- 9) Children (no safety is established for children (less experience in use))

- (2) Other precautions to be taken when taking this drug
- 1) Adherence to the specified usage and capacity
- 2) In principle, it is not to take it continuously for a long time, but if it is unavoidable to take it continuously for a long time, consult with doctors, oriental doctors, dentists, pharmacists, and oriental pharmacists.
- 3) When combined with potassium-containing preparations, licorice-containing preparations, glycyric acid-containing preparations, loop diuretics (furosemide, etacrine) or thiazide diuretics (trichloromethiazide), myopathy is likely to occur due to pseudo-aldosteronism or hypokalemia, so take it carefully.
- 4) When taken with other herbal medicines, etc., be careful about overlapping containing herbal medicines.

- (3) Precautions for Storage
- 1) Avoid direct sunlight and store it in a cool place with as little moisture as possible (must keep it sealed after use).
- 2) Keep it out of reach of children.
- 3) Take the medicine out of the original container and store it in another container, which may cause an accident or deterioration in the quality of the medicine due to misuse, thus put it in the original container and keep it tightly closed.

12. Suspension and withdrawal criteria

12.1. Suspension and withdrawal criteria

- If the conditions to be excluded from the selection of test subjects are determined after screening
- If the test subject or guardian withdraws consent to participate in the clinical trial
- In the event that a serious violation of the clinical trial plan, such as the criteria for inclusion and exclusion, is discovered during the clinical trial
- In the event of a serious adverse events/drug reaction
- When the test subject has administered the drug that is expected to affect the evaluation of the safety and effectiveness of a clinical trial drug
- In case of taking a combination-prohibited drug during a clinical trial
- In the event that the progress of clinical studies is deemed inappropriate by the judgement of the principal investigator or sub-investigator

12.2. Management in case of suspension and withdrawal

- If the test subject is suspended or withdrawn, the administration of the trial drug shall be stopped, and all data obtained up to the time of suspension and withdrawal shall be recorded in the electronic case report form (e-CRF) along with the date of suspension (final administration), reasons for suspension or withdrawn, and treatment and progress at suspension or withdrawn.

12.3. Management of violation of clinical trial plan

- The investigator must be fully aware of and thoroughly implement the clinical trial plan to prevent violations of that.
- In the event of a serious violation of the clinical trial plan, the test subject should be withdrawn from the analysis (excluding per-protocol analysis), and the relevant cases are as follows.

1) No written informed consent obtained

2) Violation of inclusion and exclusion criteria

3) If a prohibited drug is administered during the clinical trial period

4) If the main examination at the start and end of the clinical trial is missing

5) If the clinical trial drug's compliance is less than 70%

- Medication compliance (%) is defined by dividing the actually consumed tablets by the total numbers of tablets prescribed during the clinical trial period, and if this value is less than 70%, it is withdrawn.
- Other minor violations of the clinical trial plan that are not considered to affect the interpretation of research results are included in the per-protocol analysis by clearly stating the degree and reason for the violation or delay, and comprehensively considering whether the violation or delay affected the clinical trial.

13. Evaluation criteria, methods, and statistical analysis

13.1. Patient group for data analysis

13.1.1. Demographic information

- Demographic information will be assessed for all participants.

13.1.2. Safety outcomes

- Safety outcomes will be analyzed in the intention-to-treat (ITT) population.
- The original data are analyzed without correction of the data.

13.1.3. Efficacy outcomes

- Efficacy outcomes will be analyzed for both the per-protocol (PP) and ITT populations. In case of discrepancies between the efficacy outcomes of PP and ITT populations, both set of results will be presented and compared, with the PP population considered the main

analysis and the ITT population as supplementary analysis.

- The ITT population consists of participants who received at least one dose of the clinical trial drugs and underwent efficacy evaluation more than once during the treatment period. In cases of missing values due to dropouts or discontinuation before the clinical trial ends, missing data from dropout participants will be imputed using the last observation carried forward analysis.
- The PP population is a subset of the ITT population. It includes participants who consumed >70% of the prescribed doses of clinical study drugs and completed follow-up visits and corresponding outcome measurements (weeks 4, 8, and 12) during the 12-weeks treatment periods. Participants falling under a 'significant violation of clinical trial plan' will be excluded from the PP population.

13.2. Demographic data

- Data will be presented as the mean ± SD for continuous data or frequencies for categorical data.
- To confirm the validity of the random allocation, demographic and baseline values between the experimental and control groups will be compared and evaluated. We will use the independent t-test or Mann-Whitney U test for continuous outcome measures and the chi-square test, Fisher's exact test, or Cochran-Mantel-Haenszel Method for categorical outcome measures.
-

13.3. Efficacy assessment

13.3.1. Primary outcome efficacy assessment

13.3.1.1. Evaluation item

- Unstimulated Salivary Flow Rate (SFR) after 12 weeks of treatment

13.3.1.2. Statistical methods

- A 95% confidence interval (95% CI) will be used to analyze primary outcomes.
- We will calculate the 95% CI of the mean difference in the unstimulated SFR between the two groups after 12 weeks of medication. If the lower end of the 95% CI of the mean difference is greater than the non-inferiority limit (−0.21), it will be concluded that the experimental group is not inferior to the control group.
- For further analysis, if the SFR between the two groups satisfy the normality test, a t-test will be performed to compare the two groups in terms of the primary outcomes; otherwise, the Mann-Whitney U test will be performed. If there is a significant difference in baseline values between the two groups, repeated-measures analysis of covariance (ANCOVA) will be performed.

13.3.2. Secondary outcomes efficacy assessment

13.3.2.1. Evaluation items

- Stimulated SFR after 12 weeks of medication
- Differences and mean percentage changes in unstimulated and stimulated SFR from baseline (week 0) to the end of the trial (week 12)
- Differences and mean percentage change in the visual analogue scale (VAS) and 14-item Oral Health Impact Profile (OHIP-14), and from baseline (week 0) to the end of the trial (week 12).
- Differences and mean percentage changes in the uptake ratio (UR), maximum accumulation (MA), and maximum secretion (MS) on salivary scintigraphy from baseline (week 0) to the end of the trial (week 12).

13.3.2.2. Statistical methods

- To analyze the secondary outcomes, in the case of continuous outcome measures, a normality test for the distribution of data will be initially performed. In the case of non-normally distributed data, the data will be transformed to a normal distribution using the log-transformation or square root transformation methods.
- The analysis will the proceed using either parametric or non-parametric methods. Specifically, an independent T test or Mann-Whitney U test will be performed to compare values between the two groups.
- The Paired T test or Wilcoxon signed-rank test will be performed to compare intragroup continuous values.
- The group and time interaction effect of repeated-measures data will be analyzed through repeated-measures analysis of variance (ANOVA) or ANCOVA, or through the GEE model test based on the nature of the data.
- The level of significance will be set at $p < 0.05$.

13.4. Safety assessment

- It includes adverse events, clinical laboratory examination, vital signs, and physical examination.
- Safety outcomes will be analyzed in the intention-to-treat (ITT) population, and the original data are analyzed without correction of the data.
- All test subjects' vital signs, physical examination, and clinical laboratory examination results are charted and comprehensively reviewed. The investigator confirms whether there is an abnormality in each test subject, writes its clinical significance, reviews whether it is related to the test drug, and presents the results of test subjects with significant clinically

fluctuations in a separate table.

13.4.1.1. Adverse events (AE)

- Adverse events (AEs) are standardized into system organ class (SOC) and preferred term (PT) based on MedDRA ver. 23.1 (Korean).
- For AEs and drug reactions in this clinical trial, the frequency (number of test subjects), percentage, and number of occurrences are presented based on SOC and PT for each group.
- For serious AEs, the frequency, percentage, and number of occurrences are presented based on SOC for each group. Serious AEs are also summarized in a list of AEs, periods, courses, severity, outcomes, and causal relationships.
- In addition, individual lists of all test subjects with AEs are presented (including experimental/control group, reported AEs, start date/time and end date/time, course, intensity, seriousness, the association with clinical trial drugs, action taken, and final outcomes). AEs that caused discontinuation of the test are presented as a separate list. AEs are summarized using the preferred terms according to MedDRA ver. 23.1 (Korean).

13.4.1.2. Vital signs, physical examination

- The values measured at each visit and the changes from baseline test results are summarized in descriptive statistics (degree (N), median, mean, standard deviation (SD), minimum, and maximum values).

13.4.1.3. Clinical laboratory examination

- For continuous data (e.g., CBC, Clinical chemistry), the values measured at each visit and the changes from baseline test results are summarized in descriptive statistics (degree (N),

median, mean, standard deviation (SD), minimum, and maximum values). For categorical data, baseline and each-visit values are summarized and described using frequencies.

14. Safety evaluation criteria and method, and reporting method

14.1. Definition of safety-related terms

14.1.1. Adverse events (AEs)

- Adverse event (AE) refers to an undesirable and unintended sign, symptom, or disease that occurs in the test subject receiving a drug used in a clinical trial, and does not necessarily have a causal relationship with the clinical trial drug. Therefore, AEs include undesirable and unintended signs or symptoms (e.g., clinically meaningful laboratory test value abnormalities) that appear or worsen during a clinical trial, regardless of the causal relationship with the clinical trial drug.

14.1.2. Adverse Drug Reaction (ADR)

- Adverse Drug Reaction (ADR) refers to all harmful and unintended reactions occurring at any dose of drug used in clinical trials, in which a causal relationship with a clinical trial drug cannot be excluded.

14.1.3. Serious adverse events (SAEs)

- Serious adverse events (SAEs) refer to cases that fall under each of the followings among AEs or ADRs that occurred during clinical trials.
  - Death or life-threatening events
  - Hospitalization or prolonged hospitalization of test subjects
  - Permanent or serious disability or loss of function
  - Congenital anomalies or birth defects

14.2. Evaluation of adverse events (AEs)

14.2.1. Criteria for evaluating the severity of AEs

- The investigator evaluates the severity of each AE and SAE reported during the trial period. Severity is assessed in accordance with MedDRA ver. 23.1 (Korean).

Grade	
1	Mild; asymptomatic or mild symptoms; clinical or diagnostic observations only; intervention not indicated.
2	Moderate; minimal, local or noninvasive intervention indicated; limiting age-appropriate instrumental ADL*
3	Severe or medically significant but not immediately life-threatening; hospitalization or prolongation of hospitalization indicated; disabling; limiting self care ADL**
4	Life-threatening consequences; urgent intervention indicated.
5	Death related to AE.

14.2.2. Evaluation of causality of AEs

- The criteria for evaluating the causal relationship with clinical trial drugs for AEs and SAEs are as follows.
- 1) Definitely related
  - Any evidence that this drug has been administered
  - When AEs are most probably explained by the administration of this drug than by other causes
  - Abnormal reactions disappear after discontinuation of administration
  - If the re-administration result is positive (re-challenge, only if possible).
  - If the AE is consistent with the information already known about this drug or other drug of the same class

- 2) Probably related
  - Any evidence that this drug has been administered
  - If the time order of administration of this drug and the onset of AEs is reasonable
  - When AEs are more likely to be explained by the administration of this drug than by other causes
  - Abnormal reactions disappear after discontinuation of administration
- 3) Possibly related
  - Any evidence that this drug has been administered
  - If the time order of administration of this drug and the onset of AEs is reasonable
  - When it is determined that AEs are due to administration of this drug at a similar level to other possible causes
  - Abnormal reactions disappear after discontinuation of administration
- 4) Probably not related
  - No evidence that this drug has been administered
  - If there are other more likely causes for AEs
  - If the result of discontinuation of administration is negative or ambiguous
  - 5) Definitely not related
  - No evidence that this drug has been administered
  - If there is any other reason that most likely explains the AEs
  - If AEs are not lost as a result of discontinuation of administration
- 6) Unknown

14.3. Records of AEs and SAEs

- In electronic case report forms (e-CRFS), the investigator records all information related to AEs and SAEs, namely, the name of the AEs, the date of occurrence, the date of end, the

intensity, the association with the clinical trial drug, the results, the treatment status, and the SAEs.

14.4. Report of AEs

14.4.1. Report of AEs

- All AEs and SAEs are recorded in the test subject's medical records and electronic case report forms (e-CRFs), whether voluntarily reported by the test subject, learned through questions, or identified by physical or laboratory examination or other means.
- For each AE, the following are generally recorded: exact characteristics of AEs, date of manifestation, date of disappearance, severity/intensity, whether treatment is required, treatment process in such cases, the result (whether the patient has recovered from the AEs), causality with the test drug, and whether it corresponds to SAEs.
- In the case of SAEs, the investigator shall report it to the sponsor very quickly (usually within 24 hours). This is 1) to ensure the safety of the patient in clinical trials and 2) to meet the requirements of the Food and Drug Administration (FDA). The investigator not only reports the AEs to the sponsor, but also to the Institutional Review Board (IRB) in accordance with the requirements of the IRB. The IRB shall inform the investigator when to report something and what the reporting mechanism is. The investigator may also receive "information on safety-related matters" about the trial drug from the sponsor. If such safety information is received, it shall be submitted to the IRB.

14.4.2. Report of SAEs

- The sponsor shall promptly report all serious and unexpected AEs to other relevant investigators, the review committee, and the head of the Ministry of Food and Drug Safety within the period specified in any of the following.

- In the event of death or life-threatening, it must be reported with 7 days from the date the sponsor is reported or informed of this. In such cases, further detailed information shall be reported within eight days from the date of initial report.
- In the event of all other significant and unexpected AEs, the sponsor must report this within 15 days of being reported or made aware of.
- The sponsor shall periodically report additional safety information in connection with the report in any of the following until the AEs are terminated (such as the loss of the adverse drug reaction or the inability to follow-up investigation).

14.5. Follow-up of AEs and adverse drug reactions (ADRs)

- Normal/abnormalities in each clinical test value are determined by individual test subjects, and statistical verification can be performed if necessary. The degree of abnormality in the clinical test value is recorded and the relevance to the trial drug is determined. If there is a problem with the clinical test value, the patient should be followed up and clinical tests should be repeated to record whether the test value has recovered to normal.

14.6. Management and treatment of test subjects after clinical trial

- The test subjects who have completed or dropped out of this clinical trial should receive appropriate care as necessary. If AEs that require treatment occur as judged by the investigator, appropriate measures should be taken in accordance with the 'A protocol on compensation for victims'.

15. Research ethics

15.1. Institutional Review Board (IRB)

- Prior to commencement of a clinical trial, the investigator shall undergo an IRB review of

the clinical trial data book, clinical trial plan, the test subject's instructions and consent forms, methods of securing the test subject (including advertisements), and other information provided to the test subject in documentation. The IRB shall deliver the results of deliberation on the conduct of the trial to the investigator in writing prior to the start of the clinical trial.

- The principal investigator must conduct a clinical trial after obtaining approval from the IRB for the clinical trial plan and revisions or changes. In addition, events, especially AEs and SAEs, that may affect the patient's safety or the continued implementation of clinical trials, must be reported to the IRB. In accordance with the applicable IRB criteria, a report on progress, if necessary, must be submitted to the IRB, and the trial termination report must be notified to the IRB at the end of the clinical trial.

15.2. Informed consent

- Consent to the test subjects must be established prior to the implementation of any test procedure (including screening), the initiation of clinical trial-related tests, and the administration of clinical trial drugs. The informed consent with a signature and date is obtained from each test subject in accordance with the Korean Good Clinical Practice (KGCP). The investigator shall keep the informed consent of the test subjects as part of the test record.
- The consent form may be changed as appropriate. If the consent form is amended (e.g., due to a change in the test plan or due to significant new safety information), the investigator is responsible for verifying that the changed consent form has been reviewed and approved by all investigators prior to approval by the relevant IRB and that all test subjects who participated in the test have signed it.
- A record of the test subject's consent signed and dated prior to the test screening must be specified in the case report forms (CRFs) and the supporting document at the time the

informed consent is obtained.

15.3. Confidentiality

- All records that can identify the test subject must be kept confidential. All documents related to clinical trials, such as CRFs, are recorded and classified by the subject identification code (subject number, initials) rather than the test subject's name to protect the subject's privacy and ensure the confidentiality of the record, and if there is a subject's name in the medical record (pathological report, video record, etc.), it shall be deleted and stored. Even when the results of the clinical trial are published, the identity of the test subject will remain confidential, and only the subject number or initials will be recorded if the subject's individual data must be published or reported.
- Clinical trial monitoring personnel and inspectors, as well as the Ministry of Food and Drug Safety and the clinical trial review committee, can access the subject's medical records for the purpose of confirming the collected information. At this time, the information of the subject to be exposed will be handled under thorough confidentiality, and the investigator will inform the subject of this fact.

15.4. Measures for safety protection of test subjects

- The clinical trial institution shall be equipped with the facilities and professionals necessary for the implementation of this clinical trial and shall be fully prepared to properly conduct the clinical trial.
- Before registering the subject for the clinical trial, the investigator must thoroughly check the health status of each subject and check whether it is suitable to participate in the clinical trial. In addition, the investigator shall fully understand the clinical trial plan and the clinical trial drug, and conduct the clinical trial according to the plan. The investigator shall

do his/her best to ensure the safety of the subject, and in the adverse events (AEs) caused by a clinical trial, he/she shall immediately take measures to ensure that the subject receives the necessary examination and treatment. In addition, the investigator shall observe until the AEs are lost or follow-up becomes impossible, if necessary.

- If the test subject wants to receive treatment due to AEs or the investigator deems that medical treatment is necessary during the clinical trial period, he/she shall visit the clinical trial institution immediately for related examinations. If the investigator determines that the trial should be stopped by evaluating AEs and test results, or if the subject wants to stop the trial, the investigator shall promptly proceed with the procedure according to the end of the clinical trial and actively provide the subject with treatment that can alleviate the symptoms.

16. Other items necessary to conduct clinical trials safely and scientifically

In conducting clinical trials, research should be conducted under ethical and scientific consideration in compliance with the fundamental spirit of the Korean Good Clinical Practice (KGCP) and the Helsinki Declaration. Contents not presented in this clinical trial plan are in accordance with KGCP and related laws, notices, and contents of the Helsinki Declaration.

16.1. Case report forms (CRFs)

- The investigator shall prepare and maintain an appropriate and accurate case report form (CRF) designed to record all observations and other relevant data for each test subject. This includes the accurate documentation of evidence for confirmation of receipt of clinical trial drugs specified in this trial plan. The investigator shall preserve a list of test subject identification codes necessary to contact the subjects after the trial.
- This list will be kept confidential by the investigator's institution after completion of the

trial. The CRFs shall be prepared for each test subject registered for the trial, including those excluded from the screening. In this trial, all information recorded in the CRFs should be consistent with the test subject's supporting document.

- The data to be recorded shall be recorded in the CRF immediately. If it is not recorded by the end of the case, the appropriate reason for omission should be recorded. If modification on the e-CRF is necessary, the revised data, the name and signature of the modifier, the reason for modification, and the date of modification shall be recorded using the system query (automatic) and manual query (manual) of the electronic CRF (e-CRF).

16.2. Use of computer systems

- Data from clinical trials are input and managed using e-CRF (REDcap, Vanderbilt University Development, https://www.project-redcap.org/) instead of paper CRF for convenience in data processing and quality management of clinical trials, and all signatures are replaced by electronic signatures (Reason: <2015 frequently asked Q&A book related to clinical trials> (Food and Drug Administration, 2015)).

16.3. Ensuring data quality and data security

- Before the start of the clinical trial, an initiation meeting is held in which investigators participate. In this meeting, detailed discussions will be held on the research plans, research conduction, CRFs, sample collection and pre-processing methods. Monitoring personnel monitor according to the monitoring plan.
- The clinical trial coordinator and monitoring personnel shall double-check all data recorded in the CRF. If abnormalities are found, compare and review with the supporting documents. The data entered in the CRF should be double-checked according to the trial institution's Standard Operating Procedure (SOP) and data management plan. When data input is

confirmed, lock the database system.

- The query list for unresolved queries should be resolved with the investigator through clinical trial monitoring personnel. The database is modified based on signed query resolution.
- Security should be maintained for all documents such as supporting documents and CRFs obtained through the clinical trial, and investigators should not disclose such information without the consent of the sponsor.
- Since the anonymity of the test subjects must be guaranteed, the test subject number or initials are used in all documents instead of the test subject's name. Documents that can identify test subjects should also be secured by the investigator.

16.4. Management and storage of records

- Various data and records related to clinical trial shall be preserved and secured at Kyung Hee University Medical Center. The person in charge of management and storage of documents related to clinical trial shall be the principal investigator, and access shall be limited to the investigator. After completion of the trial, consent forms and other clinical trial-related documents shall be preserved for a period of at least 3 years in accordance with the Bioethics Act.

16.5. Monitoring of clinical trial institutions

- The trial monitoring is conducted (1) to protect the rights and well-being of the test subjects, (2) to determine whether the reported clinical trial-related data are accurate, complete and verifiable in comparison with the supporting document, (3) to determine whether the clinical trial is carried out in accordance with the approved plan, the KGCP, and Article 28 of the Enforcement Rules.

- Monitoring of clinical trials will be conducted through regular test subjects' visits and phone calls by monitoring personnel of the Oriental Medicine Clinical Trial Center at Kyung Hee University Korean Medicine Hospital, which has been entrusted with the task. During the visit, the monitoring personnel should basically check the original patient record, drug management record, and data storage (study file), and confirm that the blindfolding of the random number allocated for each subject's unique code is maintained. In addition, the monitoring personnel carefully examines the progress of the clinical trial and consults with the investigator if there is a problem.
- The appropriate time for these visits shall be allocated by consultation between the investigator and the monitoring personnel. The investigator shall also allow the monitoring personnel to view the original documents (such as hospital or individual charts, laboratory result records, appointment records, etc.) of the test subjects, as defined in the Korean Good Clinical Practice (KGCP).

17. References

- Masahiro UMINO, et al. Effect of Byakko-ka-Ninjin-to on Xerostomia in elderly patients -Analysis of the relationship between improvement of subjective symptom and Kampo diagnosis-. Kampo Medicine. 1994;45(1):107-113.)
- Shuji YAKUBO, et al. The Effects of Byakko-ka-ninjin-to on Patients in whom Thirst has been induced by Disopyramide Phosphate. Kampo Medicine. 1995;46(3):433-438.)
- Masaru Sakaguchi, et al. Effects of Byakko-ka-ninjin-to on salivary secretion and bladder function in rats. J Ethnopharmacol. 2005 Nov 14;102(2):164-9.
- A Niijima, et al. Effect of byakko-ka-ninjin-to on the efferent activity of the autonomic nerve fibers innervating the sublingual gland of the rat. J Auton Nerv Syst. 1997 Mar 19;63(1-2):46-50.

- Vivino FB, et al. Pilocarpine tablets for the treatment of dry mouth and dry eye symptoms in patients with Sjögren syndrome: a randomized, placebo-controlled, fixed-dose, multicenter trial. P92-01 Study Group. Arch Intern Med. 1999. PMID: 9927101 Clinical Trial.
