# Supplementary material for: Efficacy of combined administration of Baekhogainsam-Tang and low-dose pilocarpine on frequent intractable xerostomia: Study protocol for a randomized controlled trial
Source: PLoS One. 2024 Nov 11;19(11):e0307557. doi: 10.1371/journal.pone.0307557 (PMC11554038; doi:10.1371/journal.pone.0307557)
Supplement: Attachment — Submitted filename: Response to Reviewers.docx [file pone.0307557.s004.docx]

June 11, 2024

Dear Dr Hadi Ghasemi

Academic Editor

*PLOS ONE*

We wish to re-submit a revised manuscript titled “Efficacy of combined administration of Baekhogainsam-Tang and low-dose pilocarpine on frequent intractable xerostomia: Study protocol for a randomized controlled trial” by Su Il Kim et al., for consideration as a publication in the *PLOS ONE*. The manuscript number is PONE-D-24-16207.

We would like to thank editor and reviewers for their helpful comments and suggestions. We have revised our manuscript as per these comments. The following is a point-by-point response to the suggestions (bold) of the editor and reviewers (responses to the comments are provided in blue-colored text). The revised text in the manuscript has been highlighted in yellow. We also have revised our initial manuscript’s format according to the PLOS ONE’s style requirements. Additionally, figure files (fig. 1 and 2) were uploaded to PACE digital diagnostic tool and adjusted. (Resolution change to 300 PPI & TIFF file conversion to valid TIF file format).

Thank you very much for your consideration of our manuscript. We look forward to hearing from you.

Sincerely,

Young-Gyu Eun, MD, PhD

**Reviewers' comments:**

**Reviewer's Responses to Questions**

**Comments to the Author**

1. Does the manuscript provide a valid rationale for the proposed study, with clearly identified and justified research questions?

The research question outlined is expected to address a valid academic problem or topic and contribute to the base of knowledge in the field.

Reviewer #1: Yes

Reviewer #2: Yes

Reviewer #3: Yes

Reviewer #4: Yes

2. Is the protocol technically sound and planned in a manner that will lead to a meaningful outcome and allow testing the stated hypotheses?

The manuscript should describe the methods in sufficient detail to prevent undisclosed flexibility in the experimental procedure or analysis pipeline, including sufficient outcome-neutral conditions (e.g. necessary controls, absence of floor or ceiling effects) to test the proposed hypotheses and a statistical power analysis where applicable. As there may be aspects of the methodology and analysis which can only be refined once the work is undertaken, authors should outline potential assumptions and explicitly describe what aspects of the proposed analyses, if any, are exploratory.

Reviewer #1: Yes

Reviewer #2: Yes

Reviewer #3: Yes

Reviewer #4: Yes

3. Is the methodology feasible and described in sufficient detail to allow the work to be replicable?

Descriptions of methods and materials in the protocol should be reported in sufficient detail for another researcher to reproduce all experiments and analyses. The protocol should describe the appropriate controls, sample size calculations, and replication needed to ensure that the data are robust and reproducible.

Reviewer #1: Yes

Reviewer #2: Yes

Reviewer #3: Yes

Reviewer #4: Yes

4. Have the authors described where all data underlying the findings will be made available when the study is complete?

The [PLOS Data policy](https://journals.plos.org/plosone/s/materials-and-software-sharing) requires authors to make all data underlying the findings described in their manuscript fully available without restriction, with rare exception, at the time of publication. The data should be provided as part of the manuscript or its supporting information, or deposited to a public repository. For example, in addition to summary statistics, the data points behind means, medians and variance measures should be available. If there are restrictions on publicly sharing data—e.g. participant privacy or use of data from a third party—those must be specified.

Reviewer #1: Yes

Reviewer #2: Yes

Reviewer #3: Yes

**Reviewer #4: No**

[Response]

Thank you for your insightful comments.

The full protocol will be available as supplementary files. The data points behind means, medians and variance measures, anonymized dataset will be available after completion of patient recruitment and analysis.

The results of the clinical trial will be disseminated to the clinicians dealing with xerostomia. This process will be implemented on a society level (publication, public talks, news) and scientific level (peer-reviewed journals, presentations at international oral disease society).

5. Is the manuscript presented in an intelligible fashion and written in standard English?

PLOS ONE does not copyedit accepted manuscripts, so the language in submitted articles must be clear, correct, and unambiguous. Any typographical or grammatical errors should be corrected at revision, so please note any specific errors here.

Reviewer #1: Yes

Reviewer #2: Yes

Reviewer #3: Yes

Reviewer #4: Yes

6. Review Comments to the Author

Please use the space provided to explain your answers to the questions above and, if applicable, provide comments about issues authors must address before this protocol can be accepted for publication. You may also include additional comments for the author, including concerns about research or publication ethics.

You may also provide optional suggestions and comments to authors that they might find helpful in planning their study.

(Please upload your review as an attachment if it exceeds 20,000 characters)

**Reviewer #1: Appears to be a well-conceived protocol. Would the authors consider adding Cevimeline as an additional experimental group?**

[Response]

Thank you for your insightful comments and suggestions regarding our manuscript.

Although cevimeline is an FDA-approved drug for xerostomia, it is not well prescribed in Korea because it is not approved in Korea FDA. Thus, only pilocarpine was prescribed in the experimental group in this study.

**Reviewer #2: well written and much needed research on the topic. The methodology is described in detail and can be replicated easily. All relevant outcomes are measured.**

[Response]

Thank you for your insightful comments

**Reviewer #3: Dear Authors,**

**interesting study protocol for stubborn xerostomia.**

**In exclusion criteria you didn't mentioned use of antiepileptics and opioid analgesics such as tramadol which are strongly xerogenic drugs.**

**It is also very important to include only patients with healthy oral mucosa, without any evidence of infection such as oral candidiasis, which is very often in these patients and may compromise the effect of the applied therapy.**

[Response]

Thank you for your helpful comments and suggestions regarding our manuscript.

In this study, we only included (1) Patients who undergone radiation therapy to the head and neck, or (2) patients with primary Sjogren’s syndrome.

The diagnosis of secondary Sjogren’s syndrome may be subjective, thus patients taking medications causing xerostomia or with infectious or ulcerative oral diseases were excluded in this study.

We have revised related sentences in the ‘inclusion and exclusion criteria’ section of the Materials and methods.

[Previous Materials and methods]

*Inclusion and exclusion criteria*

The inclusion criteria are as follows: (1) Patients who undergone radiation therapy to the head and neck or those with Sjogren’s syndrome with positive anti-Ro/SS-A antibody reaction and unstimulated SFR ≤ 0.1 ml/min [11]; (2) unstimulated SFR ≤ 0.25 ml/min [12]; (3) VAS score for dry mouth over the past one month ≥ 4; (4) age >19 years; (5) compliance with all written informed consent.

Patients with the following past medical history are excluded: heart failure, medication-refractory hypertension (systolic blood pressure ≥ 160 mmHg or diastolic blood pressure ≥ 100 mmHg), bronchial asthma, arrhythmia accompanied by clinical symptoms or requiring treatments, coronary artery disease, chronic obstructive pulmonary diseases, chronic bronchitis, inborn errors of amino acid metabolism, hepatic encephalopathy, ophthalmic diseases (such as narrow angle glaucoma, peripheral retinopathy, and iritis). Patients taking following medications are also excluded: coumadin, heparin, warfarin, aspirin, anticholinergics, first-generation antihistamines, antidepressants with anticholinergic effects, monoamine oxidase inhibitors, diuretics, mineralocorticoids, β-adrenaline antagonist, or digitalis; patients taking low-dose aspirin or β-adrenaline antagonist can participate in the protocol based on the cardiologist’s opinion.

[Revised Material and methods (132~149 lines)]

**Inclusion and exclusion criteria**

The inclusion criteria are as follows: (1) Patients with healthy oral mucosa who undergone radiation therapy to the head and neck or those with primary Sjogren’s syndrome (positive anti-Ro/SS-A antibody reaction and/or labial salivary gland biopsy showing focal lymphocytic sialadenitis and focus score of ≥1 foci/4 mm^2^, and unstimulated SFR ≤ 0.1 ml/min) [12]; (2) unstimulated SFR ≤ 0.25 ml/min [13]; (3) VAS score for dry mouth over the past one month ≥ 4; (4) age >19 years; (5) compliance with all written informed consent.

Patients with the following past medical history are excluded [14, 15]: heart failure, medication-refractory hypertension (systolic blood pressure ≥ 160 mmHg or diastolic blood pressure ≥ 100 mmHg), bronchial asthma, arrhythmia accompanied by clinical symptoms or requiring treatments, coronary artery disease, chronic obstructive pulmonary diseases, chronic bronchitis, inborn errors of amino acid metabolism, hepatic encephalopathy, ophthalmic diseases (such as narrow angle glaucoma, peripheral retinopathy, and iritis). Patients taking following medications are also excluded. These are prohibited drug in combination with the trial drugs or drugs causing xerostomia: coumadin, heparin, warfarin, aspirin, anticholinergics, first-generation antihistamines, antidepressants with anticholinergic effects, monoamine oxidase inhibitors, diuretics, mineralocorticoids, antiepileptics, opioid analgesics, β-adrenaline antagonist, or digitalis; patients taking low-dose aspirin or β-adrenaline antagonist can participate in the protocol based on the cardiologist’s opinion.

[Added References]

14.	Kawakita T, Shimmura S, Tsubota K. Effect of oral pilocarpine in treating severe dry eye in patients with Sjögren syndrome. Asia Pac J Ophthalmol (Phila). 2015;4(2):101-5.

15.	Zimmerman TJ, Wheeler TM. Side effects and ways to avoid them. Ophthalmology. 1982;89(1):76-80.

**In outcomes section please explain more precisely how saliva will be collected and measured? Will it be with funnel and graduated test tube or with something else?**

[Response]

Thank you for your insightful comments and suggestions regarding our manuscript.

There is no funnel in our outpatient otolaryngologic clinic, and it is difficult for patients to spit directly into the graduated test tube. Thus, we used paper cup in this study.

In short, patients collected saliva in the paper cup, then the saliva collected in the paper cup was placed in the graduated test tube to measure the amount of saliva.

We have revised related sentences in the ‘primary outcome’ section of the Materials and methods.

[Previous Materials and methods]

*Primary outcome*

The primary outcome of this study is the unstimulated SFR after 12 weeks of treatment, which will be presented as mean ± standard deviation (SD) in each group, respectively. To determine the unstimulated SFR, the participants will abstain from food intake for at least one hour. The participants will need to rinse their mouth with water, held it open, and gather saliva for 5 min. SFR (ml/min) is calculated by dividing the measured amount of saliva (ml) by time (5 min).

[Revised Materials and methods (211~217 lines)]

**Primary outcome**

The primary outcome of this study is the unstimulated SFR after 12 weeks of treatment, which will be presented as mean ± standard deviation (SD) in each group, respectively. To determine the unstimulated SFR, the participants will abstain from food intake for at least one hour. The participants will need to rinse their mouth with water, held it open, and gather saliva in a paper cup for 5 min. Then, the saliva collected in the paper cup was placed in the graduated test tube to accurately measure the amount of saliva. SFR (ml/min) is calculated by dividing the measured amount of saliva (ml) by time (5 min).

**Discussion needs to be expanded.**

[Response]

Thank you for your suggestions regarding our manuscript.

We have added the interaction mechanisms between the BIT components, and the limitation of the study design to the Discussion.

[Previous Discussion]

**Discussion**

Intractable xerostomia causes persistent discomfort despite continuous treatment; however, a standardized treatment protocol has not been established yet. Pilocarpine, a sialagogue approved by the US FDA, is less effective when there are few residual salivary glands and is accompanied by many contraindications and side effects. Mild muscarinic side effects, including sweating, urinary frequency, flushing, nausea, and dizziness, seem to be common with therapeutic use and are dose-dependent [19]. Administration of low-dose pilocarpine (2.5 mg) can reduce side effects but also diminishes its effectiveness as a sialagogue [13]. BIT is an herbal medicine that has long been used to treat sore throat, thirst, and flushing; thus, it may be added as an adjuvant therapy for intractable xerostomia. However, limited research exists on its efficacy in xerostomia, especially in cases of intractable xerostomia derived from Sjögren’s syndrome or radiation therapy for head and neck cancer. Therefore, we aim to demonstrate the efficacy and safety of combined administration of BIT herbal medicine and low-dose pilocarpine compared with the administration of only high-dose pilocarpine for intractable xerostomia.

This clinical trial was designed in accordance with the SPIRIT statement [15]. This trial has been designed as a randomized, open-label, parallel-group, multi-center study. Although blinding is an important method in randomized clinical trials, clinicians and patients are aware of their group (open-label) assignments due to identifiable drugs. Open-label studies have a potential reporting bias for patient-reported outcomes such as VAS, OHIP-14, and EQ-5D-5L. However, objective outcomes, such as SFR and scintigraphy, are not affected by these factors. Additionally, to avoid selection bias in this open-label study setting, the patients will be randomly assigned across multiple centers.

In conclusion, this trial will provide essential evidence on the efficacy and safety of combined use of BIT and low-dose pilocarpine in treating intractable xerostomia. Based on the results of this clinical trial, a future larger-scale clinical trial evaluating the effectiveness and safety of BIT in treating intractable xerostomia derived from various causes will be feasible.

[Revised Discussion (367-413 lines)]

**Discussion**

Intractable xerostomia causes persistent discomfort despite continuous treatment; however, a standardized treatment protocol has not been established yet. Pilocarpine, a sialagogue approved by the US FDA, is less effective when there are few residual salivary glands and is accompanied by many contraindications and side effects. Mild muscarinic side effects, including sweating, urinary frequency, flushing, nausea, and dizziness, seem to be common with therapeutic use and are dose-dependent [24]. Administration of low-dose pilocarpine (2.5 mg) can reduce side effects but also diminishes its effectiveness as a sialagogue [17].

BIT is an herbal medicine that has long been used to treat sore throat, thirst, and flushing; thus, it may be added as an adjuvant therapy for intractable xerostomia. However, limited research exists on its efficacy in xerostomia, especially in cases of intractable xerostomia derived from Sjögren’s syndrome or radiation therapy for head and neck cancer. Therefore, we aim to demonstrate the efficacy and safety of combined administration of BIT herbal medicine and low-dose pilocarpine compared with the administration of only high-dose pilocarpine for intractable xerostomia.

In a previous study using mice, it was observed that *Anemarrhena Rhizome* and *Gypsum* (calcium sulfate) components of BIT interact to increase the salivary secretion of pilocarpine [25]. When timosaponin-AIII, the constituents of *Anemarrhena Rhizome,* was administered to mice, it showed about 175-fold more potent on salivary secretion than the only BIT extracts. Especially, the effect of timosaponin-AIII on salivary secretion was strengthened when *Gypsum* was added together, which is thought to be because the secretion of salivary gland is influenced by the intracellular calcium concentration in the acinar cells of the salivary gland.

This clinical trial was designed in accordance with the SPIRIT statement [20]. This trial has been designed as a randomized, open-label, parallel-group, multi-center study. Although blinding is an important method in randomized clinical trials, clinicians and patients are aware of their group (open-label) assignments due to identifiable drugs. Open-label studies have a potential reporting bias for patient-reported outcomes such as VAS, OHIP-14, and EQ-5D-5L. However, objective outcomes, such as SFR and scintigraphy, are not affected by these factors. Additionally, to avoid selection bias in this open-label study setting, the patients will be randomly assigned across multiple centers.

Our study has some limitations. First, there was no placebo group in this study. The aim of this study was to ascertain whether the experimental group (combined administration of BIT and low-dose pilocarpine) can demonstrate both non-inferior efficacy and fewer side effects than the control group (administration of only high-dose pilocarpine). Setting patients taking both placebo and low-dose pilocarpine as control group may present insufficient salivary stimulating effects, which can be ethically problematic. If patients taking both placebo and high-dose pilocarpine are set as control group, there is a difference in both BIT and low-dose pilocarpine, which are drugs belonging to the experimental group, thus comparative analysis may be difficult after completing patient recruitment. Therefore, the absence of placebo is to compare both aspects of efficacy and side effects between the two groups. Second, patient recruitment was not as smooth as expected, targeting only patients who underwent radiation therapy to the head and neck or with primary Sjogren’s syndrome among patients with xerostomia. Since the recruitment of patients with secondary Sjogren’s syndrome is likely to involve more subjective factors than primary Sjogren’s syndrome, we planned to recruit and analyze only patients with objective diagnostic criteria in this study. We plan to recruit and analyze comprehensive patients with xerostomia from more clinical centers in subsequent clinical trials based on the results of this study.

In conclusion, this trial will provide essential evidence on the efficacy and safety of combined use of BIT and low-dose pilocarpine in treating intractable xerostomia. Based on the results of this clinical trial, a future larger-scale clinical trial evaluating the effectiveness and safety of BIT in treating intractable xerostomia derived from various causes will be feasible.

[Added References]

25.	Kimura M, Kimura I, Chen F-J. Combined Potentiating Effects of Byako-ka-ninjin-to, Its Constituents, Rhizomes of Anemarrhena asphodeloides, Timosaponin A-III, and Calcium on Pilocarpine-Induced Salvia Secretion in Streptozocin-Diabetic Mice. Biol Pharm Bull. 1996;19(7):926-31.

**Reviewer #4: I appreciate the opportunity to review this interesting study protocol on the Efficacy of combined administration of Baekhogainsam-Tang and low-dose pilocarpine on frequent intractable xerostomia. I enjoyed reading the manuscript. I commend the authors for a number of strengths of their work, including:**

**1- Simultaneous quantitative (salivary amount) and qualitative investigations in the field of xerostomia in this study can achieve more reliable results.**

[Response]

Thank you for your insightful suggestions regarding our manuscript.

In this study, we are measuring both unstimulated and stimulated SFR to fine eligible patients who meet the inclusion criteria.

Currently, we are recording all salivary amounts, thus according to your suggestion, we plan to analyze quantitative changes in unstimulated and stimulated SFR together during the analysis process after patient recruitment is completed.

**2- A large sample size that will be taken from several medical center will strengthen the diversity of the sample.**

[Response]

Thank you for your insightful comments and suggestions regarding our manuscript.

This multi-center study was conducted in four clinical research centers in Korea.

It would be nice if it was implemented by more clinical research centers, but there has been no research on the use of both BIT herbal granules and low-dose pilocarpines. Thus, we planned to conduct this study in four centers (small groups), and then we will conduct follow-up studies in more clinical centers based on these results.

**3- The methodology is written in good details that the reproducibility of the study can be achieved.**

[Response]

Thank you for your helpful suggestions regarding our manuscript.

We wanted to help other researchers easily understand this study and plan similar studies by attaching a detailed trial study protocol translated into English.

**Considering these strengths, though, as I read the manuscript I found some areas in which I would have appreciated greater clarity. I believe the paper could be further strengthened by considering the follow items:**

**Abstract:
1- Please mention the clinical trial registration and add a summary of the statistical analysis method to the methods part. Also, please include conclusion in the abstract.**

[Response]

Thank you for your insightful comments and suggestions regarding our manuscript.

We have added clinical trial registration information and a summary of the statistical analysis methods in the abstract.

[Previous Abstract]

*Methods:* A randomized, open-label, parallel-group, multi-center trial will be conducted. A total of 120 patients with Sjogren’s syndrome having an unstimulated salivary flow rate (SFR) ≤0.1 mL/min or who have undergone radiotherapy to the head and neck with an unstimulated SFR ≤0.25 mL/min will be recruited competitively. They will be randomly allocated to either the experimental or control groups. The experimental group will receive BIT herbal granules three times and pilocarpine (2.5-mg) four times daily; meanwhile, the control group will receive only 5-mg pilocarpine four times daily for 12 weeks. The primary outcome is unstimulated SFR after 12 weeks of treatment. Secondary outcomes are stimulated SFR after 12 weeks of medication, as well as differences and mean percentage changes in unstimulated and stimulated SFR, visual analog scale, salivary scintigraphy, and questionnaires for both oral symptoms and quality of life during the clinical trial.

*Discussion:* This trial will be significant evidence on the efficacy of combined use of BIT and low-dose pilocarpine to treat intractable xerostomia.

*Clinical Trial Registration:* Clinical Research Information Service of the Republic of Korea (ISRCTN, KCT0005982). Registered 10 February 2021.

[Revised Abstract (42-57 lines)]

**Methods:** A randomized, open-label, parallel-group, multi-center trial will be conducted. A total of 120 patients with Sjogren’s syndrome having an unstimulated salivary flow rate (SFR) ≤0.1 mL/min or who have undergone radiotherapy to the head and neck with an unstimulated SFR ≤0.25 mL/min will be recruited competitively. They will be randomly allocated to either the experimental or control groups. The experimental group will receive BIT herbal granules three times and pilocarpine (2.5-mg) four times daily; meanwhile, the control group will receive only 5-mg pilocarpine four times daily for 12 weeks. The primary outcome is unstimulated SFR after 12 weeks of treatment. Secondary outcomes are stimulated SFR after 12 weeks of medication, as well as differences and mean percentage changes in unstimulated and stimulated SFR, visual analog scale, salivary scintigraphy, and questionnaires for both oral symptoms and quality of life during the clinical trial. An independent T test or Mann-Whitney U test will be performed to compare values between the two groups. The Paired T test or Wilcoxon signed-rank test will be performed to compare intragroup continuous values.

**Conclusion:** This trial will be significant evidence on the efficacy and safety of combined use of BIT and low-dose pilocarpine to treat intractable xerostomia.

**Clinical Trial Registration:** This trial has been registered with the Clinical Research Information Service of the Republic of Korea (ISRCTN, KCT0005982, Registered 10 February 2021).

**2- Since another muscarinic agent (cevimeline), have recently been approved for the treatment of dry mouth in Sjögren syndrome and ...), please revise the sentence of "Pilocarpine is a solely approved salivary sialagogue to treat intractable xerostomia" to (it seems pilocarpine is best performing sialogogue drug for treatment of intractable xerostomia)**

[Response]

Thank you for your insightful comments and suggestions regarding our manuscript.

Although cevimeline is an FDA-approved drug for xerostomia, it is not well prescribed in Korea because it is not approved in Korea FDA. We have revised related sentence in the abstract.

[Previous Abstract]

Pilocarpine is a solely approved salivary sialagogue to treat intractable xerostomia

[Revised Abstract (34-35 lines)]

Pilocarpine is an approved salivary sialagogue that is frequently prescribed for the treatment of intractable xerostomia

**3- Please write keywords in alphabetical order according to MESH.**

[Response]

Thank you for your insightful comments. We have revised keywords in alphabetical order.

[Previous Keywords]

Keywords: intractable xerostomia; dry mouth; Baekhogainsam-Tang; herbal medicine; pilocarpine

[Revised Keywords (60 line)]

**Keywords:** Baekhogainsam-Tang**;** dry mouth**;** herbal medicine; intractable xerostomia**;** pilocarpine

**Introduction:**
**1- In line 86 when you explain about BTT, please mention the side effects and toxic dose of BTT (if possible).**

[Response]

Thank you for your suggestions regarding our manuscript.

We have added the toxic dose of BIT from one previous study using rats to the Introduction.

[Added Introduction (86-89 lines)]

In a study that examined toxicity by administering BIT (2000 mg/kg/day in a laboratory setting) in rats for 13 weeks, no adverse reaction related to death or medication were observed in the administration of significant amount of BIT [10].

[Added References]

10.	Iijima O. A single oral dose toxicity study and a 13-week repeated dose study with a 4-week recovery period of TSUMURA Hochu-ekki-to (TJ-41) in rats. Jpn Pharmacol Ther. 1995;23(7):209-24.

**Material and methods:**

**1- In trial design section: The status and timeline of the study is not mentioned. (Please add how long will the overall process of the study take from the time of the initial screening of people until the results of the study are obtained?)**

[Response]

Thank you for your insightful suggestions regarding our manuscript.

The date of first participant enrolment was 5 August 2021. It is anticipated that recruitment and analysis of the trial data will be completed by December 2024. We have added related sentences in the Material and methods.

[Previous Materials and methods]

**Trial design**

This study is a prospective, randomized, open-label, parallel-group, multi-center, and controlled trial involving participants from four clinical research centers in Korea: Kyung Hee University Medical Center, Kyung Hee University Hospital in Gangdong, Inha University Hospital, and Myongji Hospital. This trial was registered with the Clinical Research Information Service of the Republic of Korea (ISRCTN, KCT0005982).

[Revised Materials and methods (109-116 lines)]

**Trial design**

This study is a prospective, randomized, open-label, parallel-group, multi-center, and controlled trial involving participants from four clinical research centers in Korea: Kyung Hee University Medical Center, Kyung Hee University Hospital in Gangdong, Inha University Hospital, and Myongji Hospital. This trial was registered with the Clinical Research Information Service of the Republic of Korea (ISRCTN, KCT0005982, Registered 10 February 2021). The current version of the protocol is version 2.6, dated 14, November 2023. First participant recruitment commenced on 5 August 2021. It is anticipated that recruitment and analysis of the trial data will be completed by December 2024.

**2- In inclusion and exclusion criteria section:**

**a. Since the users of a wide range of medicines have been mentioned as part of your exclusion criteria, and patients with secondary Sjogren's syn. require the use of a wide range of mentioned medicines due to the simultaneous suffering of other connective tissue diseases (such as SLE or RA), Can it be said that the primary Sjogren's syn. is your inclusion criteria? (Just Primary Sjogren instead of Sjogren). In that case, can minor salivary gland biopsy be added to this part (inclusion criteria) as the gold standard criteria for primary Sjogren's syn. diagnosis?**

[Response]

Thank you for your helpful comments and suggestions regarding our manuscript.

In this study, we included (1) patients with primary Sjogren’s syndrome or (2) those who undergone radiation therapy to the head and neck

We thought that the diagnosis of secondary Sjogren’s syndrome may be subject, thus we considered a plan to include only objective patients such as Sjogren’s syndrome or patients who undergone radiation therapy, and to include patients with secondary Sjogren’s syndrome in subsequent studies. Additionally, it is expected that it will be easier to recruit patients by adding information about minor salivary gland biopsy.

We have revised related sentences in the ‘inclusion and exclusion criteria’ section of the Materials and methods.

[Previous Materials and methods]

*Inclusion and exclusion criteria*

The inclusion criteria are as follows: (1) Patients who undergone radiation therapy to the head and neck or those with Sjogren’s syndrome with positive anti-Ro/SS-A antibody reaction and unstimulated SFR ≤ 0.1 ml/min [11]; (2) unstimulated SFR ≤ 0.25 ml/min [12]; (3) VAS score for dry mouth over the past one month ≥ 4; (4) age >19 years; (5) compliance with all written informed consent.

[Revised Materials and methods (132-138 lines)]

**Inclusion and exclusion criteria**

The inclusion criteria are as follows: (1) Patients with healthy oral mucosa who undergone radiation therapy to the head and neck or those with primary Sjogren’s syndrome (positive anti-Ro/SS-A antibody reaction and/or labial salivary gland biopsy showing focal lymphocytic sialadenitis and focus score of ≥1 foci/4 mm^2^, and unstimulated SFR ≤ 0.1 ml/min) [11]; (2) unstimulated SFR ≤ 0.25 ml/min [12]; (3) VAS score for dry mouth over the past one month ≥ 4; (4) age >19 years; (5) compliance with all written informed consent.

**b. Please add references to exclusion criteria.**

[Response]

Thank you for your suggestions regarding our manuscript.

We have added three references to exclusion criteria.

[Previous Materials and methods]

Patients with the following past medical history are excluded: heart failure, medication-refractory hypertension (systolic blood pressure ≥ 160 mmHg or diastolic blood pressure ≥ 100 mmHg), bronchial asthma, arrhythmia accompanied by clinical symptoms or requiring treatments, coronary artery disease, chronic obstructive pulmonary diseases, chronic bronchitis, inborn errors of amino acid metabolism, hepatic encephalopathy, ophthalmic diseases (such as narrow angle glaucoma, peripheral retinopathy, and iritis). Patients taking following medications are also excluded: coumadin, heparin, warfarin, aspirin, anticholinergics, first-generation antihistamines, antidepressants with anticholinergic effects, monoamine oxidase inhibitors, diuretics, mineralocorticoids, β-adrenaline antagonist, or digitalis; patients taking low-dose aspirin or β-adrenaline antagonist can participate in the protocol based on the cardiologist’s opinion.

Additionally, we exclude patients with clinical laboratory test results falling within the following ranges: hemoglobin < 8.0 g/dL, aspartate transaminase (AST) or alanine transaminase (ALT) > 3 times the upper limit of each hospital standard, total bilirubin > 2 times the upper limit of each hospital standard, creatinine >1.5 times the upper limit of each hospital standard, or creatinine clearance ≤ 60 mL/min. Other exclusion criteria are as follows

[Revised Materials and methods (139-154 lines)]

Patients with the following past medical history are excluded [14, 15]: heart failure, medication-refractory hypertension (systolic blood pressure ≥ 160 mmHg or diastolic blood pressure ≥ 100 mmHg), bronchial asthma, arrhythmia accompanied by clinical symptoms or requiring treatments, coronary artery disease, chronic obstructive pulmonary diseases, chronic bronchitis, inborn errors of amino acid metabolism, hepatic encephalopathy, ophthalmic diseases (such as narrow angle glaucoma, peripheral retinopathy, and iritis). Patients taking following medications are also excluded. These are prohibited drug in combination with the trial drugs or drugs causing xerostomia: coumadin, heparin, warfarin, aspirin, anticholinergics, first-generation antihistamines, antidepressants with anticholinergic effects, monoamine oxidase inhibitors, diuretics, mineralocorticoids, antiepileptics, opioid analgesics, β-adrenaline antagonist, or digitalis; patients taking low-dose aspirin or β-adrenaline antagonist can participate in the protocol based on the cardiologist’s opinion.

Additionally, we exclude patients with clinical laboratory test results falling within the following ranges: hemoglobin < 8.0 g/dL, aspartate transaminase (AST) or alanine transaminase (ALT) > 3 times the upper limit of each hospital standard, total bilirubin > 2 times the upper limit of each hospital standard, creatinine >1.5 times the upper limit of each hospital standard, or creatinine clearance ≤ 60 mL/min. Other exclusion criteria are as follows [16]

[Added References]

14.	Kawakita T, Shimmura S, Tsubota K. Effect of oral pilocarpine in treating severe dry eye in patients with Sjögren syndrome. Asia Pac J Ophthalmol (Phila). 2015;4(2):101-5.

15.	Zimmerman TJ, Wheeler TM. Side effects and ways to avoid them. Ophthalmology. 1982;89(1):76-80.

16.	Amado D, Cavalheiro E. Hormonal and gestational parameters in female rats submitted to the pilocarpine model of epilepsy. Epilepsy Res. 1998;32(1-2):266-74.

**3- In Interventions section:**

**a. Considering that you have mentioned in the introduction of the article that "no clinical trials have studied the efficacy of combined administration of BTT and low-dose pilocarpine for intractable xerostomia", is there any previous study on possible medical interactions or anti-synergism effects of BTT and pilocarpine (even as invitro studies)? (please mention)**

[Response]

Thank you for your insightful suggestions regarding our manuscript.

In previous studies, there have been experiments in mice on the combination effect of BIT and pilocarpine, but clinical trials have not been conducted on combination therapy in humans with xerostomia.

We have revised related sentences in the Introduction.

[Previous Introduction]

In two previous animal experiments, BIT increased salivary gland secretion [7] and improved the effect of anticholinergic agents on salivary secretion but had no influence on the bladder [9]. After the administration of BIT for xerostomia in elderly patients, 60% of 30 patients reported an improvement in symptoms [10]. Thus, it is thought that BIT can compensate for the insufficient effect of low-dose pilocarpine. However, to the best of our knowledge, no clinical trials have studied the efficacy of combined administration of BIT and low-dose pilocarpine for intractable xerostomia, such as Sjogren’s syndrome, or in those who received radiotherapy for head and neck cancer.

[Revised Introduction (84-96 lines)]

In two previous animal experiments, BIT increased salivary gland secretion [7] and improved the effect of anticholinergic agents on salivary secretion but had no influence on the bladder [9]. In a study that examined toxicity by administering BIT (2000 mg/kg/day in a laboratory setting) in rats for 13 weeks, no adverse reaction related to death or medication were observed in the administration of significant amount of BIT [10]. In other words, when pilocarpine and BIT were administered in combination in mice, BIT increased the salivary secretion of pilocarpine without significant side effects. In a previous study in human, only BIT was administered in 30 elderly patients with xerostomia, of which 60% reported an improvement in symptoms [11]. Thus, it is thought that BIT can compensate for the insufficient effect of low-dose pilocarpine. However, to the best of our knowledge, no clinical trials have studied the efficacy of combined administration of BIT and low-dose pilocarpine for human patients with intractable xerostomia, such as Sjogren’s syndrome, or in those who received radiotherapy for head and neck cancer.

[Added References]

10.	Iijima O. A single oral dose toxicity study and a 13-week repeated dose study with a 4-week recovery period of TSUMURA Hochu-ekki-to (TJ-41) in rats. Jpn Pharmacol Ther. 1995;23(7):209-24.

**b. Please include a reference for exact amounts of BTT components.**

[Response]

Thank you for your suggestions regarding our manuscript. We have added a reference for amounts of BIT components to the legend of Table 1.

[Previous Table 1 legend]

**Table 1.** Components of Baekhogainsam-Tang (BIT) granules (4 g / dose)

[Revised Table 1 legend (194 line)]

**Table 1.** Components of Baekhogainsam-Tang (BIT) granules (4 g / dose) [18]

[Added References]

18.	Lee S, Cho H. Comparison of shanghanlun. Seoul: KMediACs publishing house. 2015;24, 28, 203.

**Discussion:**
**1- Please add the limitations of the study design**

[Response]

Thank you for your suggestions regarding our manuscript.

We have added the limitations of the study design to the Discussion.

[Added Discussion (395-409 lines)]

Our study has some limitations. First, there was no placebo group in this study. The aim of this study was to ascertain whether the experimental group (combined administration of BIT and low-dose pilocarpine) can demonstrate both non-inferior efficacy and fewer side effects than the control group (administration of only high-dose pilocarpine). Setting patients taking both placebo and low-dose pilocarpine as control group may present insufficient salivary stimulating effects, which can be ethically problematic. If patients taking both placebo and high-dose pilocarpine are set as control group, there is a difference in both BIT and low-dose pilocarpine, which are drugs belonging to the experimental group, thus comparative analysis may be difficult after completing patient recruitment. Therefore, the absence of placebo is to compare both aspects of efficacy and side effects between the two groups. Second, patient recruitment was not as smooth as expected, targeting only patients who underwent radiation therapy to the head and neck or with primary Sjogren’s syndrome among patients with xerostomia. Since the recruitment of patients with secondary Sjogren’s syndrome is likely to involve more subjective factors than primary Sjogren’s syndrome, we planned to recruit and analyze only patients with objective diagnostic criteria in this study. We plan to recruit and analyze comprehensive patients with xerostomia from more clinical centers in subsequent clinical trials based on the results of this study.

**2- Please add the interaction mechanism between the BTT components and to explain the combined effects of that components with pilocarpine (for example: interaction between some Anemarrhena components and CaCl2 is a way to explain the combined effects of BTT with pilocarpine and ....)**

[Response]

Thank you for your helpful comments and suggestions regarding our manuscript.

Among the main components of the BIT granules*,* *Anemarrhena Rhizome* and *Gypsum* have been studied through mouse experiments as the main components related to salivary secretion.

We have added related contents and reference to the Discussion.

[Added Discussion (381-387 lines)]

In a previous study using mice, it was observed that *Anemarrhena Rhizome* and *Gypsum* (calcium sulfate) components of BIT interact to increase the salivary secretion of pilocarpine [20]. When timosaponin-AIII, the constituents of *Anemarrhena Rhizome,* was administered to mice, it showed about 175-fold more potent on salivary secretion than the only BIT extracts. Especially, the effect of timosaponin-AIII on salivary secretion was strengthened when *Gypsum* was added together, which is thought to be because the secretion of salivary gland is influenced by the intracellular calcium concentration in the acinar cells of salivary gland.

[Added References]

25.	Kimura M, Kimura I, Chen F-J. Combined Potentiating Effects of Byako-ka-ninjin-to, Its Constituents, Rhizomes of Anemarrhena asphodeloides, Timosaponin A-III, and Calcium on Pilocarpine-Induced Salvia Secretion in Streptozocin-Diabetic Mice. Biol Pharm Bull. 1996;19(7):926-31.

7. PLOS authors have the option to publish the peer review history of their article ([what does this mean?](https://journals.plos.org/plosone/s/editorial-and-peer-review-process#loc-peer-review-history)). If published, this will include your full peer review and any attached files.

If you choose “no”, your identity will remain anonymous but your review may still be made public.

**Do you want your identity to be public for this peer review?** For information about this choice, including consent withdrawal, please see our [Privacy Policy](https://www.plos.org/privacy-policy).

Reviewer #1: **Yes:** Elliot Abt

Reviewer #2: No

Reviewer #3: No

Reviewer #4: No
