# Supplementary material for: Efficacy of combined administration of Baekhogainsam-Tang and low-dose pilocarpine on frequent intractable xerostomia: Study protocol for a randomized controlled trial
Source: PLoS One. 2024 Nov 11;19(11):e0307557. doi: 10.1371/journal.pone.0307557 (PMC11554038; doi:10.1371/journal.pone.0307557)
Supplement: Attachment — Submitted filename: Response to Reviewers2.docx [file pone.0307557.s005.docx]

June 28, 2024

Dear Dr Hadi Ghasemi

Academic Editor

*PLOS ONE*

We wish to re-submit a revised manuscript titled “Efficacy of combined administration of Baekhogainsam-Tang and low-dose pilocarpine on frequent intractable xerostomia: Study protocol for a randomized controlled trial” by Su Il Kim et al., for consideration as a publication in the *PLOS ONE*. The manuscript number is PONE-D-24-16207R1.

We would like to thank editor and reviewers for their helpful comments and suggestions. We have revised our manuscript as per these comments. The following is a point-by-point response to the suggestions (bold) of the editor and reviewers (responses to the comments are provided in blue-colored text). The revised text in the manuscript has been highlighted in yellow.

Thank you very much for your consideration of our manuscript. We look forward to hearing from you.

Sincerely,

Young-Gyu Eun, MD, PhD

**Reviewers' comments:**

**Reviewer's Responses to Questions**

**Comments to the Author**

1. Does the manuscript provide a valid rationale for the proposed study, with clearly identified and justified research questions?

The research question outlined is expected to address a valid academic problem or topic and contribute to the base of knowledge in the field.

Reviewer #3: Yes

Reviewer #4: Yes

Reviewer #5: Yes

2. Is the protocol technically sound and planned in a manner that will lead to a meaningful outcome and allow testing the stated hypotheses

The manuscript should describe the methods in sufficient detail to prevent undisclosed flexibility in the experimental procedure or analysis pipeline, including sufficient outcome-neutral conditions (e.g. necessary controls, absence of floor or ceiling effects) to test the proposed hypotheses and a statistical power analysis where applicable. As there may be aspects of the methodology and analysis which can only be refined once the work is undertaken, authors should outline potential assumptions and explicitly describe what aspects of the proposed analyses, if any, are exploratory.

Reviewer #3: Yes

Reviewer #4: Yes

Reviewer #5: No

3. Is the methodology feasible and described in sufficient detail to allow the work to be replicable?

Descriptions of methods and materials in the protocol should be reported in sufficient detail for another researcher to reproduce all experiments and analyses. The protocol should describe the appropriate controls, sample size calculations, and replication needed to ensure that the data are robust and reproducible.

Reviewer #3: Yes

Reviewer #4: Yes

Reviewer #5: Yes

4. Have the authors described where all data underlying the findings will be made available when the study is complete?

The [PLOS Data policy](https://journals.plos.org/plosone/s/materials-and-software-sharing) requires authors to make all data underlying the findings described in their manuscript fully available without restriction, with rare exception, at the time of publication. The data should be provided as part of the manuscript or its supporting information, or deposited to a public repository. For example, in addition to summary statistics, the data points behind means, medians and variance measures should be available. If there are restrictions on publicly sharing data—e.g. participant privacy or use of data from a third party—those must be specified.

Reviewer #3: Yes

Reviewer #4: Yes

Reviewer #5: Yes

5. Is the manuscript presented in an intelligible fashion and written in standard English?

PLOS ONE does not copyedit accepted manuscripts, so the language in submitted articles must be clear, correct, and unambiguous. Any typographical or grammatical errors should be corrected at revision, so please note any specific errors here.

Reviewer #3: Yes

Reviewer #4: Yes

Reviewer #5: Yes

6. Review Comments to the Author

Please use the space provided to explain your answers to the questions above and, if applicable, provide comments about issues authors must address before this protocol can be accepted for publication. You may also include additional comments for the author, including concerns about research or publication ethics.

You may also provide optional suggestions and comments to authors that they might find helpful in planning their study.

(Please upload your review as an attachment if it exceeds 20,000 characters)

**Reviewer #3: Dear Authors,**

**no additional comments. All requested corrections have been entered in the text.**

**Thank you.**

**Best regards**

**Reviewer #4: Thanks to the precious authors for their great accuracy and interesting manuscript, many required items have been well added to the text of the manuscript.**

**Reviewer #5: This is an unblinded randomized noninferiority study of 5 mg pilocarpine vs. 2.5 mg pilocarpine plus herbal concoction. I have several statistical questions:**

**1. The randomization is stratified block but what are the stratification variables. A stratified randomization should be followed by a stratified analysis of the primary outcome, yet I see nothing on that.**

[Response]

Thank you for your helpful comments and suggestions regarding our manuscript.

When the patient recruitment is completed and the primary outcome is analyzed, ANCOVA analysis including each clinical trial center as a covariate will be performed. Additionally, sub-analysis will be performed on each of the patient groups recruited by each trial center.

**2. Blocks of size 4. In an unblinded study, the investigator can determine the deterministic assignments in the tail of the block. That is why we use random block sizes.**

[Response]

Thank you for your insightful suggestions regarding our manuscript.

The use of random block sizes was initially discussed when planning this study. However, to prevent the occurrence of bias in patient allocation while competitively recruiting patients from each institution, the block size was set to 4. In a follow-up study, we will consider modified random block sizes to prevent knowing the patient allocation in advance as much as possible.

**3. "Opaque envelopes" were used in the 1948 streptomycin trial, but today we use a handy app to perform on site randomization in real time.**

[Response]

Thank you for your helpful comments and suggestions regarding our manuscript.

This multi-center study was conducted in only four centers using “Opaque envelopes”. Based on the results of this study, we will plan a follow-up study in more clinical trial centers, and at this time we will introduce a handy app.

**4. There is no discussion of why the trial must be unblinded. Surely the patients can take tablets without knowing what they are. How about 2 2.5 mg pills versus a 2.5 mg pill and herbal supplement? Why introduce bias in a study?**

[Response]

Thank you for your helpful comments and suggestions regarding our manuscript.

At first, we planned a blinding trial and tried to make placebo or mimics for BIT and pilocarpine. However, considering the characteristics of BIT granules (flavor, taste, quantity, etc), it was very difficult to make it with the same formulation as pilocarpine. Additionally, apart from manufacturing BIT as a capsule, half dose pilocarpine should also be manufactured as a capsule due to the case of taking half dose pilocarpine in experimental group, which was also difficult to the nature of pilocarpine. In the follow-up study, we will discuss the production of placebo with more institutions for blinding trial.

We have added related contents to the Discussion.

[Previous Discussion]

Our study has some limitations. First, there was no placebo group in this study. The aim of this study was to ascertain whether the experimental group (combined administration of BIT and low-dose pilocarpine) can demonstrate both non-inferior efficacy and fewer side effects than the control group (administration of only high-dose pilocarpine). Setting patients taking both placebo and low-dose pilocarpine as control group may present insufficient salivary stimulating effects, which can be ethically problematic. If patients taking both placebo and high-dose pilocarpine are set as control group, there is a difference in both BIT and low-dose pilocarpine, which are drugs belonging to the experimental group, thus comparative analysis may be difficult after completing patient recruitment. Therefore, the absence of placebo is to compare both aspects of efficacy and side effects between the two groups.

[Revised Discussion (395-402 lines)]

Our study has some limitations. First, there was no placebo group in this study. The aim of this study was to ascertain whether the experimental group (combined administration of BIT and low-dose pilocarpine) can demonstrate both non-inferior efficacy and fewer side effects than the control group (administration of only high-dose pilocarpine). We planned a blinding trial and tried to make placebo or mimics for BIT and pilocarpine. However, considering the characteristics of BIT granules, it was very difficult to make it with the same formulation as pilocarpine. Additionally, half dose pilocarpine should also be manufactured as a capsule due to the case of taking half dose pilocarpine in experimental group, which was also difficult to the nature of pilocarpine. Thus, the trial was unblinded.

**5. The final paragraph is in the past tense as though the trial has already occurred.**

[Response]

Thank you for your suggestions regarding our manuscript.

We had revised related sentences in the abstract.

[Previous Introduction]

**Clinical Trial Registration:** This trial has been registered with the Clinical Research Information Service of the Republic of Korea (ISRCTN, KCT0005982, Registered 10 February 2021).

[Revised Introduction (84-96 lines)]

**Clinical Trial Registration:** The Clinical Research Information Service of the Republic of Korea (ISRCTN, KCT0005982). Registered on 10 February 2021.

7. PLOS authors have the option to publish the peer review history of their article ([what does this mean?](https://journals.plos.org/plosone/s/editorial-and-peer-review-process#loc-peer-review-history)). If published, this will include your full peer review and any attached files.

If you choose “no”, your identity will remain anonymous but your review may still be made public.

**Do you want your identity to be public for this peer review?** For information about this choice, including consent withdrawal, please see our [Privacy Policy](https://www.plos.org/privacy-policy).

Reviewer #3: No

Reviewer #4: **Yes:** Fereshteh Najar-karimi

Reviewer #5: No
